# Synthesis and evaluation of potent yaku'amide A analogs†

**DOI:** 10.1039/d1sc05992k

**Published:** 2022-01-03

**Authors:** Concordia C. L. Lo, Daniel Joaquin, Diego A. Moyá, Alexander Ramos, David W. Kastner, Stephen M. White, Blake L. Christensen, Joseph G. Naglich, William J. Degnen, Steven L. Castle

**Affiliations:** Department of Chemistry and Biochemistry, Brigham Young University Provo UT 84602 USA scastle@chem.byu.edu; Bristol Myers Squibb, Research & Early Development, Mechanistic Pharmacology-Leads Discovery & Optimization Rte 206 & Province Line Rd Princeton NJ 08543 USA; Spectrix Analytical Services USA

## Abstract

Two full-length analogs of the anticancer peptide yaku'amide A (1a) and four partial structures have been synthesized. These analogs were identified by computational studies in which the three *E*- and *Z*-ΔIle residues of the natural product were replaced by the more accessible dehydroamino acids ΔVal and ΔEnv. Of the eight possible analogs, modeling showed that the targeted structures 2a and 2b most closely resembled the three-dimensional structure of 1a. Synthesis of 2a and 2b followed a convergent route that was streamlined by the absence of ΔIle in the targets. Screening of the compounds against various cancer cell lines revealed that 2a and 2b mimic the potent anticancer activity of 1a, thereby validating the computational studies.

## Abstract 2

Simplified full-length analogs of yaku'amide A were designed with the aid of computations and then synthesized. The analog that was predicted to most closely resemble the structure of the natural product mimicked its anticancer activity.

## Graphical abstract

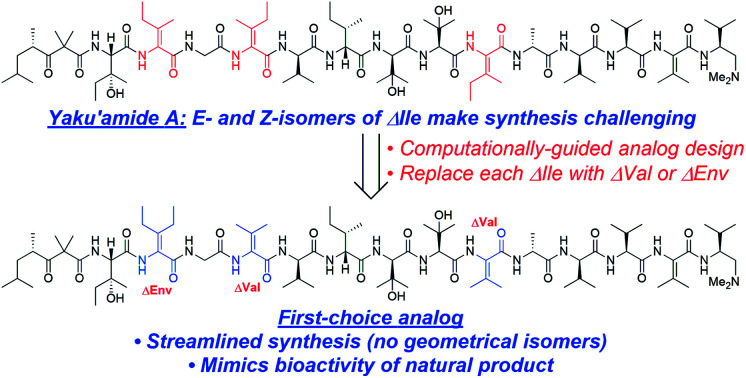

## Introduction

**Fig. 1:**
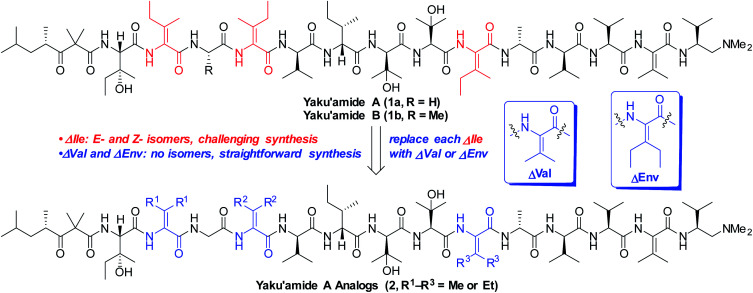
Yaku'amide A and analogs containing symmetrical bulky ΔAAs.

The linear peptide yaku'amide A (1a, Fig. 1) was isolated by Matsunaga and co-workers from the deep-sea sponge *Ceratopsion* sp.^1^ It contains several unusual β-*tert*-hydroxy amino acids (β-OHAAs) and tetrasubstituted dehydroamino acids (ΔAAs). It strongly inhibits the growth of P388 murine leukemia cells (IC_50_ = 14 ng mL^−1^) and has exhibited a unique activity profile when screened against the JFCR39 cancer cell line panel.^1^ These data suggest that 1a functions *via* a mode of action that is distinct from other anticancer agents.

Yaku'amide A and its close relative yaku'amide B (1b, Fig. 1) have attracted attention by virtue of their singular structural features, intriguing bioactivity, and scarcity in nature. In 2013, Inoue and co-workers synthesized the originally proposed structure of 1a and assigned the configuration of its *N*-terminal acyl subunit (NTA).^2*a*^ Then, while constructing 1b they determined that the structures of both yaku'amides had been misassigned. Their meticulous efforts resulted in the first total syntheses of the yaku'amides and established that the configurations of four residues (d- and l-β-OHVal, d- and l-Val) had been transposed.^2*b*^ Inoue and co-workers later discovered that 1b reduces cellular ATP levels by simultaneously inhibiting ATP synthesis and promoting ATP hydrolysis through binding to the complex mitochondrial enzyme F_0_F_1_-ATP synthase.^2*c*^ Recently, they have devised a solid-phase synthesis of 1b (ref. 3a) and demonstrated that the *E*/*Z* stereochemistry of the ΔIle residues modulates its anticancer activity.^3*b*^

Inoue's groundbreaking total syntheses of 1a and 1b illustrate the challenges inherent in constructing unsymmetrical tetrasubstituted ΔAAs such as *E*- and *Z*-ΔIle. These residues readily isomerize *via* azlactone intermediates when activated for peptide couplings.^4^ To prevent *E*/*Z* isomerization, Inoue and co-workers initially resorted to a lengthy sequence of reactions involving backbone amide protection that was required for each of the three ΔIle residues present in 1a and 1b.^2^ They subsequently streamlined this sequence^3*a*^ and eliminated the need for backbone protection,^3*b*^ but specialized and air-sensitive building blocks are necessary to execute the required Staudinger ligations. In our recent total synthesis of 1a, we devised a one-pot process involving *anti* dehydration, azide reduction, and O → N acyl transfer to forge the ΔIle residues and generate amide bonds at their *C*-termini without recourse to backbone amide protection or synthetically challenging building blocks.^5^ This strategy thwarted *E*/*Z* isomerization and enabled an efficient route to yaku'amide A, but our synthesis was still too lengthy to produce sufficient quantities of the natural product for in-depth studies of its mode of action.

We closely examined the structure of 1a in order to identify more accessible analogs that would retain its shape and presumably its bioactivity. While the three synthetically challenging ΔIle residues were strong candidates for removal, we postulated that they play a critical role in establishing the three-dimensional structure of 1a. Their low-energy conformations are likely limited in number by the high levels of A_1,3_-strain that are characteristic of tetrasubstituted alkenes. This phenomenon should confer a rigid and well-defined structure^6^ on yaku'amide A. Accordingly, we deemed it necessary to retain bulky ΔAAs in designed analogs of 1a.

In contrast to ΔIle, the symmetrical bulky ΔAAs dehydrovaline and dehydroethylnorvaline (ΔVal and ΔEnv, Fig. 1) are easier to construct due to their lack of geometrical isomers. Since they approximate the size of ΔIle, we reasoned that they could be surrogates for the three ΔIle residues present in yaku'amide A. The resulting analogs could be synthesized in significantly fewer steps than the natural product and should be useful tools for probing its mode of action by virtue of closely resembling its three-dimensional shape. Herein, we report the computationally-guided design and synthesis of two full-length yaku'amide A analogs containing ΔVal and ΔEnv in place of ΔIle along with the evaluation of their anticancer properties. We also investigated the bioactivity of key yaku'amide A partial structures.

## Results and discussion

We recognized that replacing each of the three ΔIle residues of 1a with either ΔVal or ΔEnv would result in a total of eight possible full-length analogs. We turned to computational chemistry to determine which of these compounds would most closely resemble the three-dimensional structure of the natural product. To approximate 1a and its analogs, we employed the ONIOM^7^ QM:MM method as implemented in Gaussian 09,^8^ which divides the system of interest into three layers: high, medium, and low. The high layer is treated with the most accurate method and the low layer is treated with a computationally cheaper method. Using ONIOM, we divided 1a and each potential analog into high and low layers, which were treated with quantum mechanics (QM) and molecular mechanics (MM), respectively. We employed the B3LYP 6-311g(d,p) basis set^9^ for the QM region comprised of the dehydroamino acids, and we treated all other amino acids using MM with the AMBER96 force field.^10^ The partial charges for all nonstandard residues in the MM region were assigned using R.E.D. tools,^11^ and the harmonic stretch, bond, and torsional angle parameters were generated with AMBER tools.^12^ We computed the final structures in a dielectric continuum treated with the IEF-PCM solvation model to approximate the effects of water.^13^

**Fig. 2:**
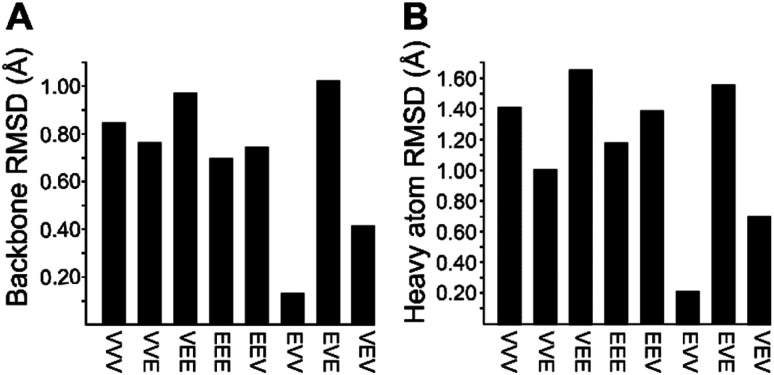
RMSD values generated by (A) superimposing the backbone atoms of 1a on each of its eight possible analogs, and by (B) accounting for the differences between ΔIle, ΔVal, and ΔEnv by superimposing all heavy atoms (backbone and side chain) common to 1a and each analog.

**Fig. 3:**
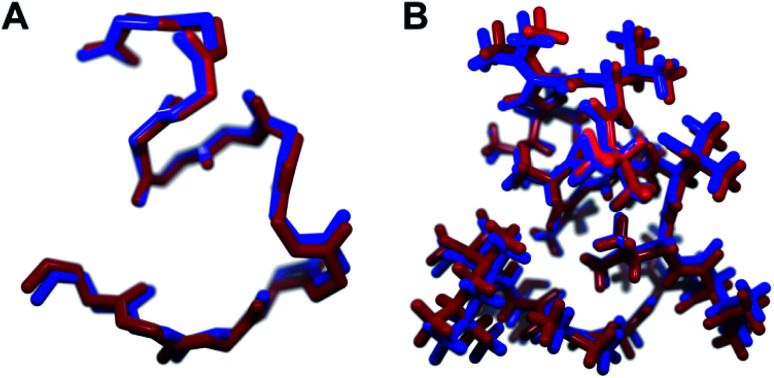
The minimized structures of 1a and 2a superimposed showing (A) only the backbone atoms, or (B) all atoms common to both compounds.

Each analog of 1a was assigned a three-letter abbreviation, where E represents ΔEnv and V represents ΔVal. Each letter signifies a residue replacing a ΔIle residue of 1a, with the substitutions listed in order from the *N*- to the *C*-terminus. We used the optimized structures of these eight compounds to calculate two separate root mean square deviation (RMSD) values *via* the VMD software^14^ by aligning the structure of 1a with that of each analog using either the backbone atoms (Fig. 2A) or all heavy atoms common to both molecules being compared (Fig. 2B). Performing two separate RMSD calculations was advantageous because the relative impact of the backbone orientation *versus* that of the side chains on the bioactivity of 1a is unknown. As expected, the RMSD calculations that included all common heavy atoms produced higher values due to the greater flexibility of the side chains relative to the backbone. Of the eight possible analogs, EVV (hereafter known as 2a) best conserved the three-dimensional structure of yaku'amide A (Fig. 2 and 3).^15^ Compound VEV (hereafter known as 2b) was selected as the second-best analog of 1a (Fig. 2).

**Scheme 1:**
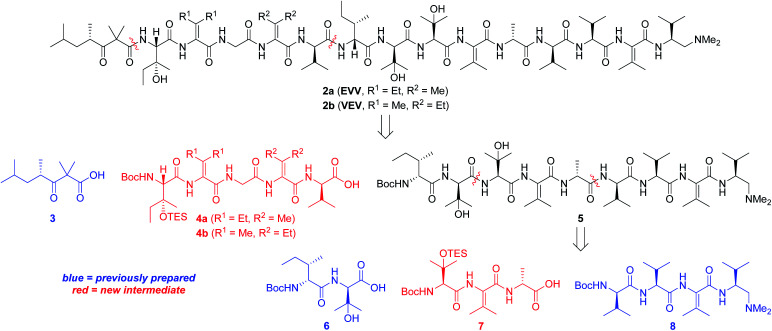
Retrosynthesis of yaku'amide A analogs.

Our strategy for synthesizing 1a (ref. 5) could be readily adapted to access the two targeted analogs, as shown in the retrosynthetic analysis that is summarized in Scheme 1. Disconnection of the target compounds at the indicated amide bonds revealed four subunits: an *N*-terminal acyl group (3) and a right-hand nonapeptide (5) common to 2a and 2b along with two different left-hand pentapeptides 4a and 4b. The nonapeptide was then dissected into dipeptide 6, tripeptide 7, and tetrapeptide 8. Intermediates 3, 6, and 8 were all employed in our total synthesis of 1a, so this plan only required three new subunits: 7, 4a, and 4b. These fragments would likely be much easier to construct than the corresponding intermediates used in our yaku'amide A total synthesis owing to the replacement of each native ΔIle residue with either ΔVal or ΔEnv.

**Scheme 2:**
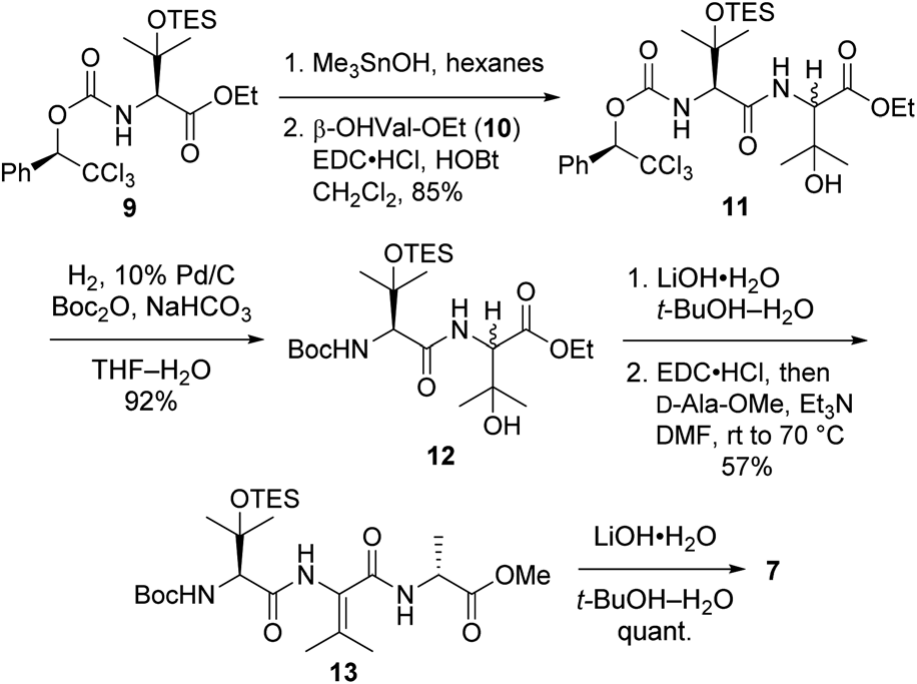
Synthesis of tripeptide 7.

Tripeptide 7, which is common to both 2a and 2b, was prepared as outlined in Scheme 2. Saponification of l-β-OHVal derivative 9 (ref. 5) required Me_3_SnOH^16^ due to the sensitivity of its chiral carbamate moiety to bases that are commonly used to hydrolyze esters. Coupling of the resulting acid to racemic β-OHVal-OEt (10)^5,17^ furnished dipeptide 11 as a mixture of diastereomers. Hydrogenolysis of 11 in the presence of Boc_2_O cleaved the trichloromethylated benzyl carbamate and replaced it with a Boc group. Concomitant scission of the TES ether was prevented by including NaHCO_3_ in the reaction mixture. The resulting dipeptide 12 was saponified under standard conditions, and the crude acid was subjected to the one-pot dehydration–amidation protocol that we devised for the synthesis of 1a.^5^ First, exposure of the acid to EDC·HCl served to dehydrate the tertiary alcohol and activate the carboxylate, triggering cyclization to form an azlactone. Then, addition of d-Ala-OMe and Et_3_N along with heating of the reaction mixture facilitated azlactone ring-opening and delivered ΔVal-containing tripeptide 13. Notably, the dehydration step caused both epimers of 12 to converge to a single alkene product. Finally, saponification of 13 afforded the key tripeptide acid 7.

**Scheme 3:**
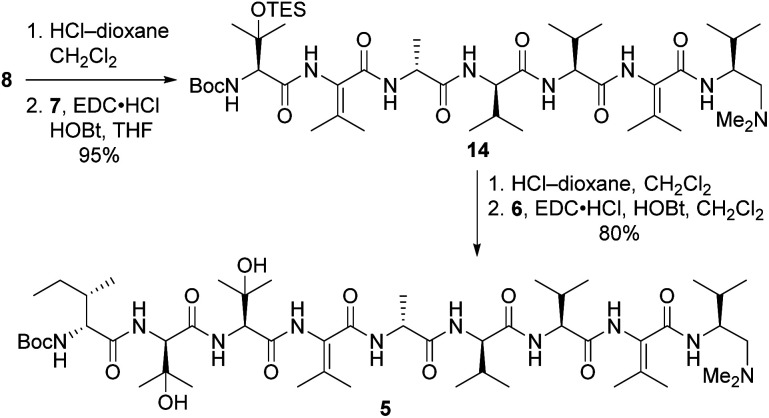
Synthesis of nonapeptide 5.

Tripeptide 7 was combined with the previously constructed subunits 6 and 8 (ref. 5) to furnish nonapeptide 5 as depicted in Scheme 3. Acidic removal of the Boc moiety from tetrapeptide 8 and subsequent coupling to 7 delivered heptapeptide 14 in excellent yield. Then, exposure of 14 to HCl simultaneously cleaved the Boc and TES groups from its *N*-terminal l-β-OHVal residue. Coupling of the resulting crude amine with dipeptide 6 afforded nonapeptide 5 in good yield. HPLC analysis of the heptapeptide and nonapeptide did not reveal any evidence of epimerization in the fragment couplings.

**Scheme 4:**
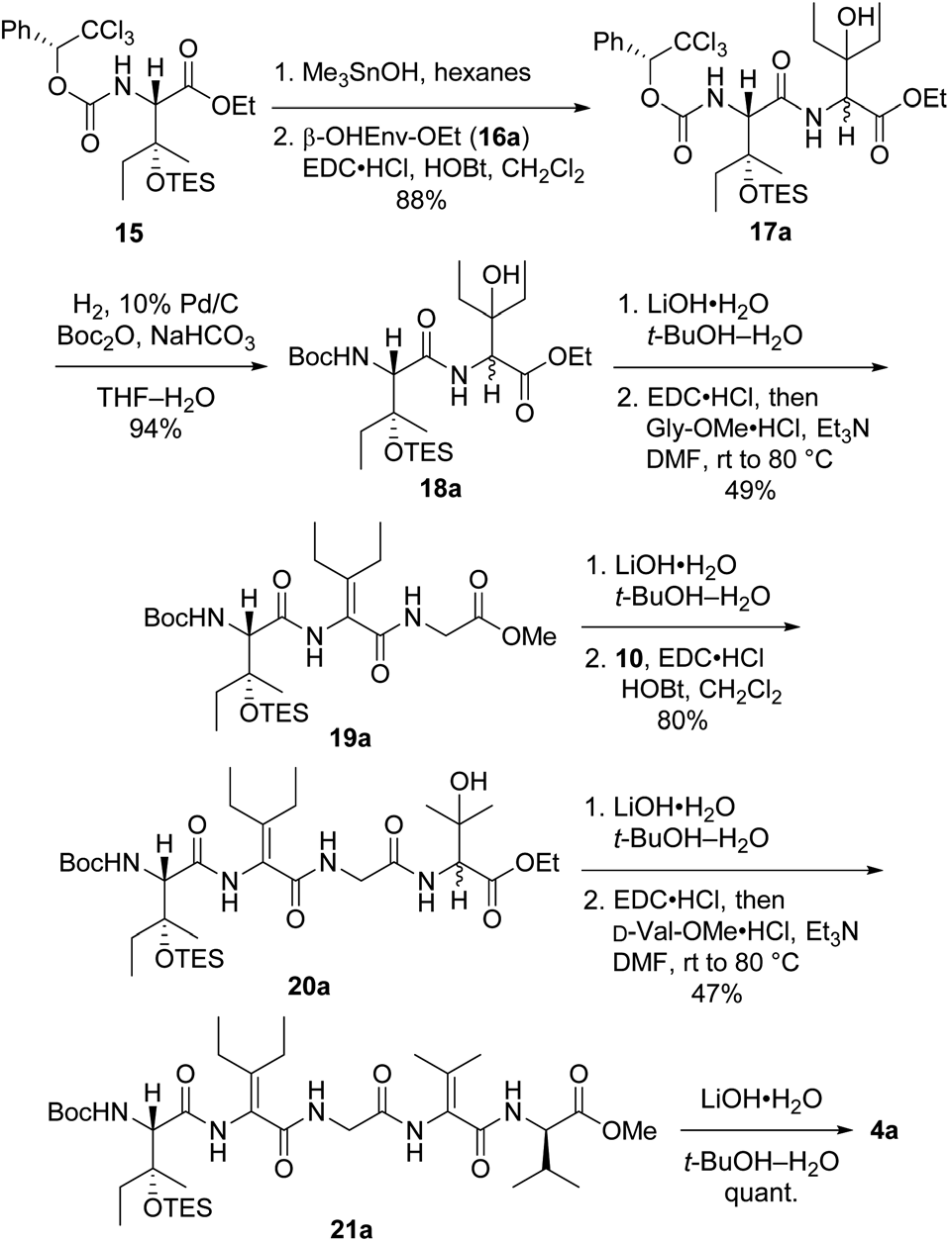
Synthesis of pentapeptide 4a.

Synthesis of first-choice analog 2a required pentapeptide 4a, which was constructed as shown in Scheme 4. Me_3_SnOH-mediated hydrolysis of β-OHIle derivative 15 (ref. 5) was followed by coupling of the crude carboxylic acid to racemic β-OHEnv-OEt (16a),^18^ delivering dipeptide 17a as a mixture of diastereomers. Swapping the chiral carbamate for a Boc group then furnished 18a in excellent yield. Saponification and subsequent dehydration–amidation with Gly-OMe were challenging due to the hindered *C*-terminal residue. Thus, a slightly lower yield was obtained relative to the dehydration–amidation of dipeptide 12 depicted in Scheme 2. The remaining amino acids ΔVal and d-Val were attached to 19a*via* a similar sequence involving coupling followed by one-pot dehydration–amidation. Saponification of the resulting pentapeptide 21a revealed carboxylic acid 4a.

**Scheme 5:**
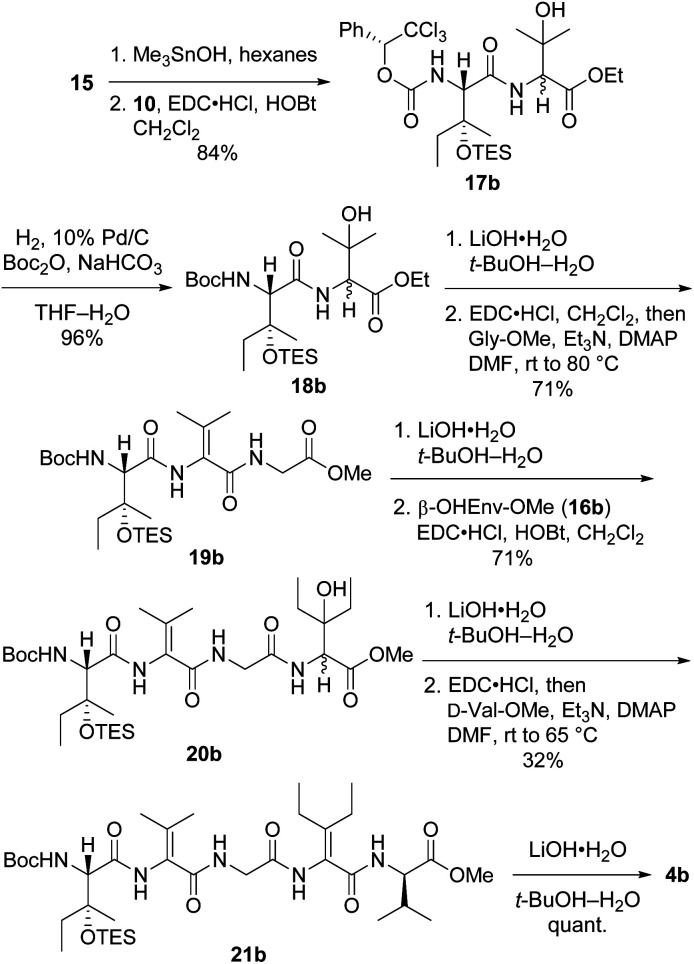
Synthesis of pentapeptide 4b.

Preparation of pentapeptide 4b required for the second-choice analog 2b resembled the construction of 4a shown above with a few key exceptions that are presented in Scheme 5. Conversion of dipeptide 18b into ΔVal-containing tripeptide 19b was most readily accomplished with DMAP as an additive to promote ring-opening amidation of the azlactone intermediate. The high yield of this process relative to analogous transformations in the syntheses of 7 (see Scheme 2) and 4a (see Scheme 4) is likely due to the use of less hindered coupling partners (ΔVal and Gly). Conversely, formation of pentapeptide 21b involved the lowest-yielding dehydration–amidation sequence owing to the highly hindered ΔEnv and d-Val coupling partners. This process was further complicated by retro aldol scission of the β-OHEnv residue during saponification of tetrapeptide 20b. Utilizing methyl ester 16b (ref. 19) instead of ethyl ester 16a that was employed in synthesizing 4a mitigated but did not eliminate this problem.

**Scheme 6:**
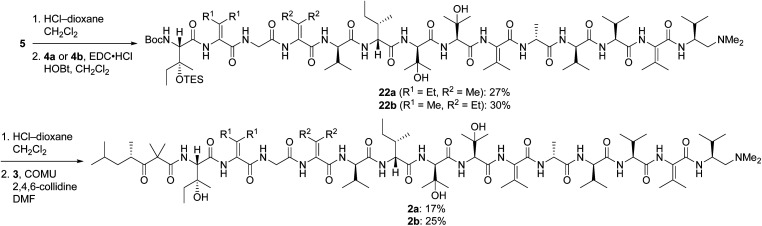
Completion of the syntheses of 2a and 2b.

Pentapeptides 4a and 4b were coupled with the free amine derived from nonapeptide 5 and elaborated into the targeted analogs 2a and 2b as illustrated in Scheme 6. Although the deprotection and coupling conditions were identical to those employed in the total synthesis of yaku'amide A,^2,5^ lower yields were obtained in the current reactions. Apparently, the subtle structural differences between the building blocks employed in the total synthesis and those used in the current study impacts the yields of these late-stage peptide couplings. Fortunately, ample quantities of 5, 4a, and 4b were available, allowing us to produce 2a and 2b in amounts sufficient for evaluation of their anticancer activity. Thus, optimization of the final two peptide couplings was deemed unnecessary.

**Scheme 7:**
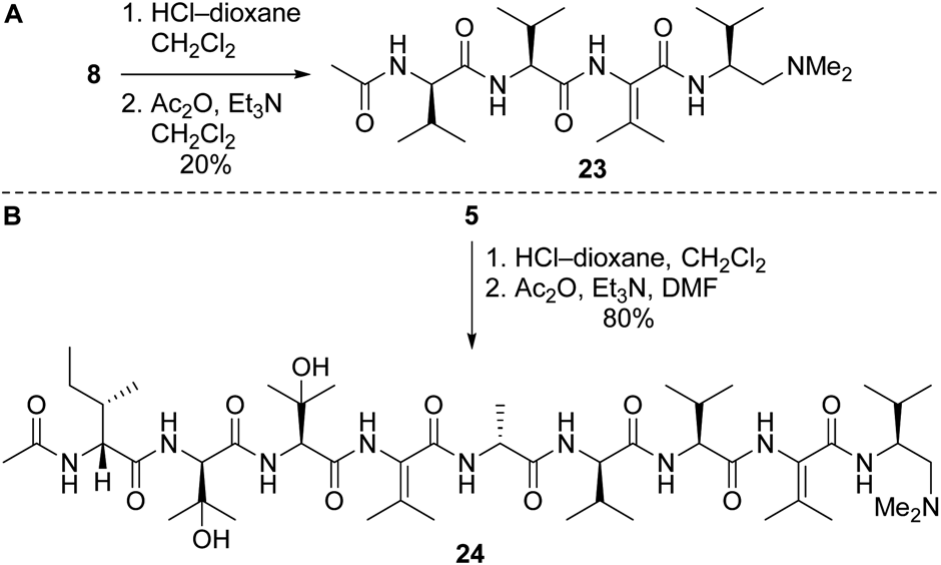
Syntheses of partial structures 23 (right) and 24 (central-right).

Our route to 2a and 2b provided access to partial structures that could be used to determine if the pharmacophore of the yaku'amides is localized to a single region of the molecules or if the full-length structures are required for bioactivity. Right-hand and central-right partial structures 23 and 24 were easily obtained by acetylation of the previously prepared tetrapeptide 8 and nonapeptide 5, respectively (Scheme 7). We elected not to optimize the acetylation of 8, as the abundance of this tetrapeptide enabled us to prepare the requisite amounts of 23 despite the low yield. We were pleased to discover that acetylation of 5 was high-yielding.

**Scheme 8:**
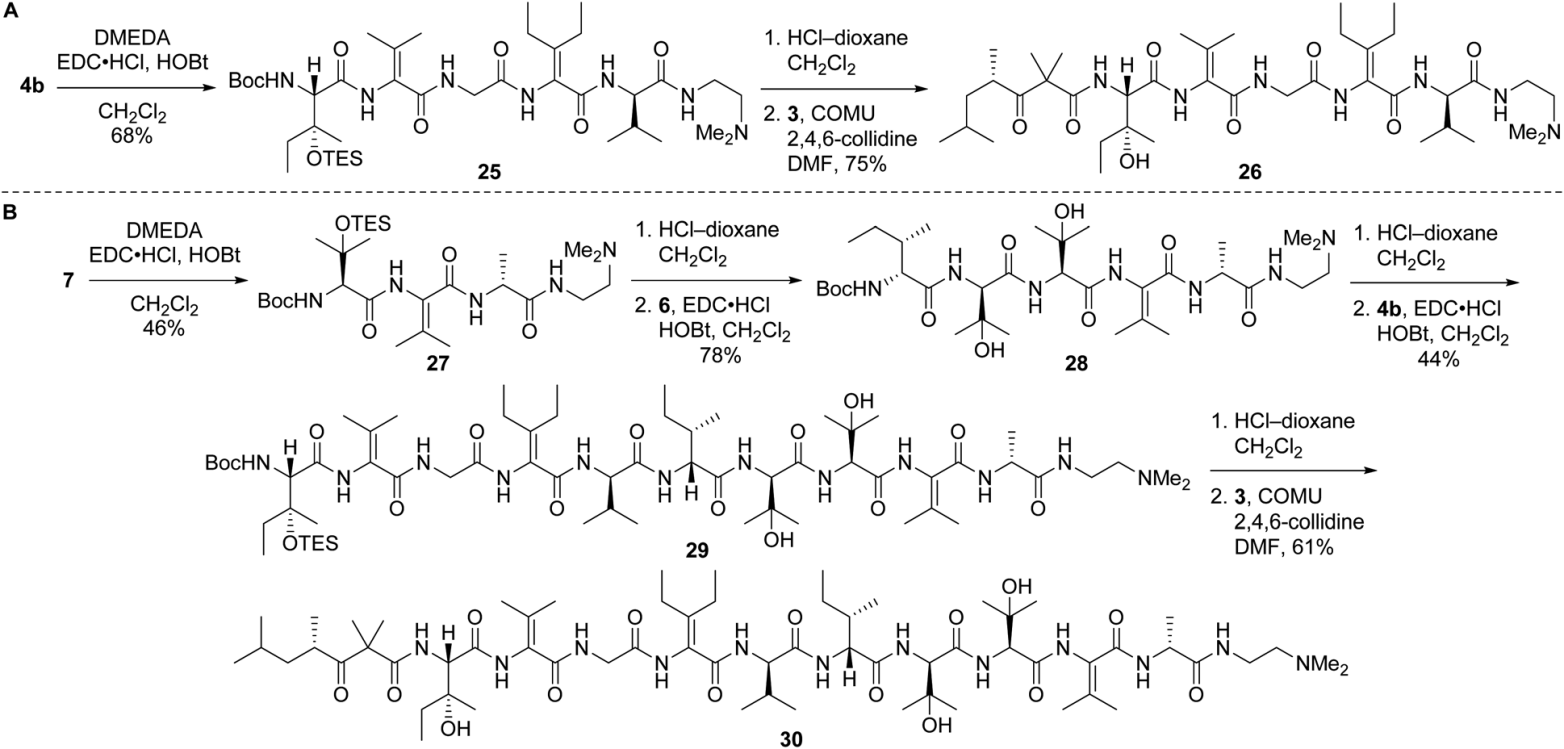
Syntheses of partial structures 26 (left) and 30 (left-central).

Left-hand and left-central partial structures 26 and 30 were constructed as outlined in Scheme 8. Capping of pentapeptide 4b with *N*,*N*-dimethylethylenediamine (DMEDA, a simplified version of the *C*-terminal amino moiety present in yaku'amide A and its full-length analogs) furnished 25 in good yield. Then, HCl-mediated cleavage of the Boc and TES groups followed by coupling of the resulting primary amine with acid 3 delivered left-hand partial structure 26. Preparation of the left-central partial structure commenced with the high-yielding union of DMEDA-capped tripeptide 27 and dipeptide 6. Boc deprotection of the resulting central fragment 28 and subsequent coupling with 4b afforded 29 in moderate yield. Acidic deprotection of this intermediate and coupling with 3 proceeded smoothly to produce the targeted partial structure 30.

Inoue and co-workers recently observed slow retro-aldol reactions of unnatural *E*/*Z* isomers of yaku'amides upon storage in the presence of dilute acetic acid.^20^ This cleavage occurs at the β-OH residues that are adjacent to the ΔAAs and is presumably the result of increased steric hindrance (*i.e.*, A_1,3_ strain) caused by the unnatural alkene isomers. We did not observe retro-aldol scission of any of our yaku'amide A full-length analogs or partial structures; however, we did not expose them to dilute acetic acid for prolonged periods of time. Thus, it is possible that our compounds would exhibit the same lability as Inoue's compounds under similar storage conditions.

**Fig. 4:**
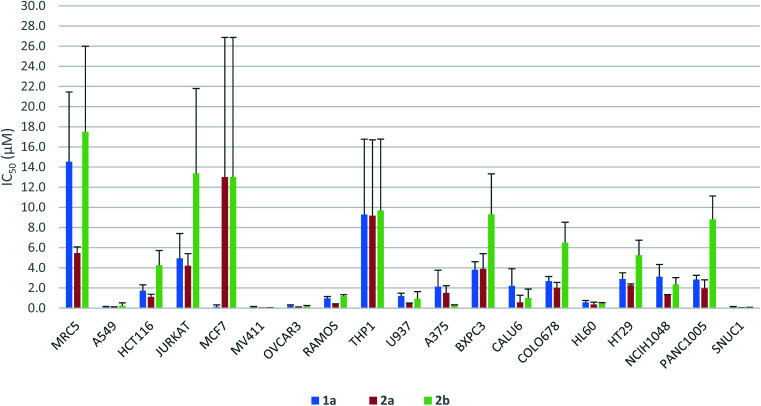
Antiproliferative activity profiles of 1a and its full-length analogs 2a and 2b.

The antiproliferative activity profiles of 1a, its full-length analogs 2a and 2b, and its partial structures (23, 24, 26, and 30) were determined by *in vitro* screening in 72 hours MTS cell proliferation assays against a panel of 18 different human cancer cell lines. The partial structures were essentially inactive (IC_50_ > 25 μM in almost all cases),^21^ suggesting that the entire sequence of yaku'amide A is required for its bioactivity. Apart from the MCF7 cell line in which only 1a was potent, both 2a and 2b exhibited a similar pattern of activity to yaku'amide A, demonstrating their suitability as mimics of the natural product (Fig. 4). All three peptides were potent inhibitors of the A549, MV411, OVCAR3, HL60, and SNUC1 cell lines (IC_50_ = 50–250 nM). Analog 2a displayed increased potency (*ca.* 2–4-fold) compared to 2b against most of the cell lines, and its activity profile was more similar to that of the natural product. These results are consistent with our original prediction that 2a would mimic the three-dimensional structure of 1a more closely than 2b. The three peptides were also evaluated for their antiproliferative activity against the lung fibroblast MRC5 cell line. The encouraging lack of potency against these noncancerous cells suggests that yaku'amide A and related analogs possess a satisfactory therapeutic window for use as anticancer agents.

## Conclusions

In an attempt to identify synthetically accessible full-length analogs of the potent anticancer peptide yaku'amide A, we performed computational studies in which its *E*- and *Z*-ΔIle residues were replaced by the symmetrical bulky ΔAAs ΔVal and ΔEnv. Of the eight candidate structures, EVV (2a) and VEV (2b) emerged as promising mimics of the natural product. We then synthesized these peptides *via* a convergent route modelled on our total synthesis of 1a. Replacement of the somewhat cumbersome chemistry required to install *E*- and *Z*-ΔIle by the straightforward one-pot dehydration–amidation suitable for generating ΔVal and ΔEnv streamlined the syntheses of key intermediates. Although the final peptide couplings were low-yielding, the abundance of key intermediates 5, 4a, and 4b due to their efficient construction allowed us to overcome this obstacle and prepare sufficient 2a and 2b to perform anticancer assays. Our syntheses of these full-length yaku'amide A analogs also provided access to four partial structures of the natural product.

Bioassays revealed that both 2a (EVV) and 2b (VEV) mimic the anticancer activity profile of 1a. Thus, these accessible analogs should be useful probes of the intriguing mode of action of the yaku'amides.^2*c*^ The data shown in Fig. 4 demonstrate that 2a more closely imitated the anticancer activity of 1a than did 2b. This result is consistent with our computationally derived hypothesis that 2a would serve as the best mimic of the three-dimensional structure of 1a. Importantly, this result provides some validation for our computational methods and suggests that they could be employed to design potent analogs of other complex bioactive peptides.

## Data availability

All experimental procedures, spectral data, bioassay data, and computational data are available in the ESI.†

## Author contributions

S. L. C. devised the project, with C. C. L. L. and D. W. K. providing critical input. D. W. K. performed the computational studies. C. C. L. L., D. J., D. A. M., A. R., S. M. W., and B. L. C. synthesized the analogs and partial structures. J. G. N. and W. J. D. performed the anticancer assays. S. L. C. wrote the manuscript with contributions from all authors.

## Conflicts of interest

There are no conflicts to declare.

## Supplementary Material

SC-013-D1SC05992K-s001

## References

[cit1] Ueoka R., Ise Y., Ohtsuka S., Okada S., Yamori T., Matsunaga S. (2010). J. Am. Chem. Soc..

[cit2] Kuranaga T., Sesoko Y., Sakata K., Maeda N., Hayata A., Inoue M. (2013). J. Am. Chem. Soc..

[cit3] Itoh H., Miura K., Kamiya K., Yamashita T., Inoue M. (2020). Angew. Chem., Int. Ed..

[cit4] Stohlmeyer M. M., Tanaka H., Wandless T. J. (1999). J. Am. Chem. Soc..

[cit5] Cai Y., Ma Z., Jiang J., Lo C. C. L., Luo S., Jalan A., Cardon J. M., Ramos A., Moyá D. A., Joaquin D., Castle S. L. (2021). Angew. Chem., Int. Ed..

[cit6] Jalan A., Kastner D. W., Webber K. G. I., Smith M. S., Price J. L., Castle S. L. (2017). Org. Lett..

[cit7] Dapprich S., Komiromi I., Byun K. S., Morokuma K., Frisch M. J. (1999). J. Mol. Struct.: THEOCHEM.

[cit8] Frisch M. J. , et al., Gaussian 09, Gaussian, Inc., Wallingford, CT, 2010

[cit9] Plumley J. A., Dannenberg J. J. (2011). J. Comput. Chem..

[cit10] Cornell W. D., Cieplak P., Bayly C. I., Gould I. R., Merz Jr K. M., Ferguson D. M., Spellmeyer D. C., Fox T., Caldwell J. W., Kollman P. A. (1995). J. Am. Chem. Soc..

[cit11] Dupradeau F.-Y., Pigache A., Zaffran T., Savineau C., Lelong R., Grivel N., Lelong D., Rosanski W., Cieplak P. (2010). Phys. Chem. Chem. Phys..

[cit12] Case D. A. , et al., AMBER 2018, University of California, San Francisco, 2018

[cit13] Cossi M., Barone V., Cammi R., Tomasi J. (1996). Chem. Phys. Lett..

[cit14] (b) http://www.ks.uiuc.edu/Research/vmd/

[cit15] Durrant J. A. (2019). Bioinformatics.

[cit16] Nicolaou K. C., Nevalainen M., Zak M., Bulat S., Bella M., Safina B. S. (2003). Angew. Chem., Int. Ed..

[cit17] Ma Z., Naylor B. C., Loertscher B. M., Hafen D. D., Li J. M., Castle S. L. (2012). J. Org. Chem..

[cit18] Jiang J., Luo S., Castle S. L. (2015). Tetrahedron Lett..

[cit19] Compound 16b was prepared by the same procedure used to synthesize known compound 16a (see ref. 18 and ESI†)

[cit20] Kamiya K., Itoh H., Inoue M. (2021). J. Nat. Prod..

[cit21] Please see ESI† for the complete screening data set

