# Supplementary material for: Synthesis and evaluation of potent yaku'amide A analogs
Source: Chem Sci. 2022 Jan 3;13(7):1899–905. doi: 10.1039/d1sc05992k (PMC8848768; doi:10.1039/d1sc05992k)
Supplement: SC-013-D1SC05992K-s001 [file SC-013-D1SC05992K-s001.pdf]

Electronic Supplementary Material (ESI) for Chemical Science.
This journal is © The Royal Society of Chemistry 2022

Supporting Information

Synthesis and Evaluation of Potent Yaku'amide A Analogs

Concordia C. L. Lo,[a] Daniel Joaquin,[a] Diego A. Moyá,[a] Alexander Ramos,[a] David W. Kastner,[a] Stephen M. White,[a] Blake L. Christensen,[a] Joseph G. Naglich,[b] William J. Degnen[c] and Steven L. Castle*[a]

[a]*Department of Chemistry and Biochemistry*
Brigham Young University, Provo, Utah, 84602

[b]*Bristol Myers Squibb, Research & Early Development, Mechanistic Pharmacology-Leads Discovery & Optimization*
Route 206 and Province Line Road, Princeton, NJ 08543

[c]*Spectrix Analytical Services*

Table of Contents

General Experimental Details	S2
Experimental Procedures and Spectral Data	S2–S38
Description of Anticancer Assays and Data	S38–S41
Computational Data	S42–S54
^{1}H and ^{13}C NMR Spectra	S55–S104

General Experimental Details

Dichloromethane, *N*,*N*-dimethylformamide, methanol, and tetrahydrofuran were dried by passage through a solvent drying system containing cylinders of activated alumina.[1] Chloroform was dried by storage over a mixture of activated 4Å MS and anhydrous K_2CO_3. Other solvents and reagents were purchased from commercial vendors and used without purification. Flash chromatography was carried out using 60–230 mesh silica gel. ^{1}H NMR spectra were acquired on a 500 MHz spectrometer with chloroform (7.27 ppm) or dimethyl sulfoxide (2.50 ppm) as internal references. Signals are reported as follows: s (singlet), d (doublet), t (triplet), q (quartet), dd (doublet of doublets), br s (broad singlet), m (multiplet). Coupling constants are reported in hertz (Hz). ^{13}C NMR spectra were acquired on a spectrometer operating at 125 MHz with chloroform (77.23 ppm) or dimethyl sulfoxide (39.51 ppm) as internal references. Infrared spectra were obtained on an FT-IR spectrometer. Mass spectral data were obtained using ESI techniques.

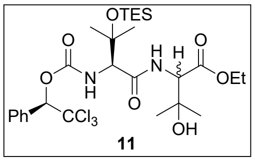

Ethyl 3-Hydroxy-3-methyl-2-((*S*)-3-methyl-2-((((*R*)-2,2,2-trichloro-1-phenylethoxy)carbonyl)amino)-3-((triethylsilyl)oxy)butanamido)butanoate (11). A suspension of **9**[2] (175.0 mg, 0.332 mmol) and Me_3SnOH (288.0 mg, 1.592 mmol, 4.8 equiv) in hexanes (10 mL, pretreated with Na_2SO_4 for 12 h) under Ar was stirred at 60 °C for 48 h. The solvent was concentrated *in vacuo*, and the residue was treated with Et_2O (12 mL). The mixture was filtered through Celite, (washed with 50 mL of Et_2O), and the filtrate was concentrated *in*

[1] A. B. Pangborn, M. A. Giardello, R. H. Grubbs, R. K. Rosen and F. J. Timmers, *Organometallics*, 1996, **15**, 1518.

[2] Y. Cai, Z. Ma, J. Jiang, C. C. L. Lo, S. Luo, A. Jalan, J. M. Cardon, A. Ramos, D. A. Moyá, D. Joaquin and S. L. Castle, *Angew. Chem. Int. Ed.*, 2021, **60**, 5162.

vacuo to afford the crude carboxylic acid as a pale yellow oil that was used directly in the next step without further purification.

A solution of the crude carboxylic acid in anhydrous CH_2Cl_2 (3.0 mL) at 0 °C under Ar was treated with amine **10**[2,3] (109 mg, 0.676 mmol, 2.0 equiv), HOBt (ca. 20% H_2O content, 114 mg, 0.675 mmol, 2.0 equiv), and EDC•HCl (130 mg, 0.678 mmol, 2.0 equiv). The resulting mixture was stirred at 0 °C under Ar for 4 h. The reaction was quenched by the addition of sat aq $NaHCO_3$ (3 mL) and H_2O (2 mL), and the layers were separated. The aqueous layer was extracted with CH_2Cl_2 (4 × 5 mL), and the combined organic layers were dried (Na_2SO_4) and concentrated *in vacuo*. Flash chromatography (10 mL of SiO_2, 0–1.5% MeOH in CH_2Cl_2 gradient elution) afforded **11** (181 mg, 0.282 mmol, 85%) as a colorless oil that was a 1:1 mixture of diastereomers: ^1H NMR ($CDCl_3$, 500 MHz, minor rotamers present, data for major rotamer of each diastereomer) δ 7.61 (d, J = 7.6 Hz, 2H), 7.44–7.34 (m, 3H), 7.14 (d, J = 8.3 Hz, 1H), 6.32 (s, 1H), 6.10 and 6.04 (2d, J = 7.6 and 6.7 Hz, 1H), 4.49 and 4.40 (2d, J = 8.7 and 8.1 Hz, 1H), 4.28–4.17 (m, 2H), 4.16 (d, J = 7.7 Hz, 1H), 2.64 and 2.62 (2s, 1H), 1.42 and 1.41 (2s, 3H), 1.32–1.24 (m, 6H), 1.21 and 1.20 (2s, 3H), 1.19 (s, 3H), 1.03–0.94 (m, 9H), 0.74–0.64 (m, 6H); ^{13}C NMR ($CDCl_3$, 125 MHz) δ 171.4 and 170.9, 170.0 and 169.6, 154.7 and 154.5, 133.0, 129.7 (2C), 129.5, 127.9 (2C), 99.7 and 99.6, 83.6 and 83.5, 72.0 and 71.4, 63.7, 62.9, 61.6 and 61.5, 60.3, 27.5 and 27.3, 27.1 and 26.7, 26.4 and 26.1, 25.0, 14.2 and 14.1, 7.0 (3C), 6.4 (3C); IR (film) ν_{max} 3349, 2956, 2876, 1738, 1667, 1505, 1375, 1202, 1059 cm^{-1}; HRMS (ESI) *m/z* 641.1984 (MH^+, $C_{27}H_{43}Cl_3N_2O_7SiH^+$ requires 641.1978).

[3] Z. Ma, B. C. Naylor, B. M. Loertscher, D. D. Hafen, J. M. Li and S. L. Castle, *J. Org. Chem.*, 2012, **77**, 1208.

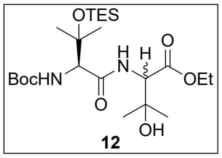

Ethyl 2-((*S*)-2-((*tert*-Butoxycarbonyl)amino)-3-methyl-3-((triethylsilyl)oxy)butanamido)-3-hydroxy-3-methylbutanoate (12). A suspension of **11** (80.0 mg, 0.125 mmol) in a mixture of THF (2.0 mL) and sat aq $NaHCO_3$ (1.0 mL) was treated with 10% Pd/C (30.2 mg, 0.38 wt equiv) and Boc_2O (28.1 mg, 0.129 mmol, 1.03 equiv) at rt under Ar. The resulting mixture was stirred at rt under H_2 (200 psi) for 24 h, then diluted with H_2O (4 mL), and extracted with EtOAc (4 × 6 mL). The combined organic layers were dried (Na_2SO_4) and concentrated *in vacuo*. Flash chromatography (11 mL of SiO_2, 0–1.5% MeOH in CH_2Cl_2 gradient elution) afforded **12** (56.1 mg, 0.114 mmol, 92%) as a colorless oil that was a 1:1 mixture of diastereomers: ^{1}H NMR ($CDCl_3$, 500 MHz) δ 7.33 and 7.14 (2 br s, 1H), 5.48 and 5.45 (2 br s, 1H), 4.57 and 4.48 (2d, *J* = 8.3 and 8.4 Hz, 1H), 4.28–4.19 (m, 2H), 4.15 and 4.04 (2 br s, 1H), 2.86 and 2.78 (2 br s, 1H), 1.45 and 1.44 (2s, 9H), 1.38 and 1.35 (2s, 3H), 1.32–1.25 (m, 12H), 0.98 (t, *J* = 7.9 Hz, 9H), 0.71–0.65 (m, 6H); ^{13}C NMR ($CDCl_3$, 125 MHz) δ 171.0, 170.9, 156.1, 79.7, 76.4, 75.4, 72.1 and 71.7, 61.4, 60.2 and 60.1, 28.2 (3C), 27.5, 27.2, 26.8, 26.6 and 26.5, 14.1, 7.0 (3C), 6.4 (3C); IR (film) ν_{max} 3257, 2954, 2876, 1747, 1646, 1513, 1366, 1165, 1052 cm^{-1}; HRMS (ESI) *m/z* 491.3152 (MH^+, $C_{23}H_{46}N_2O_7SiH^+$ requires 491.3147).

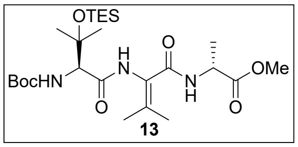

Methyl (2-((*S*)-2-((*tert*-Butoxycarbonyl)amino)-3-methyl-3-((triethylsilyl)oxy)butanamido)-3-methylbut-2-enoyl)-D-alaninate (13). A solution of **12** (40.1 mg, 0.0817 mmol) in *t*-BuOH (800 μL) was treated with LiOH•H_2O (17.4 mg, 0.415 mmol, 5.1 equiv) and H_2O (300 μL), then stirred at rt for 6 h. The resulting mixture was acidified to pH 4~5 by the addition of 1 N HCl, diluted with H_2O (2 mL), and extracted with EtOAc (3 × 5 mL). The

combined organic layers were dried (Na_2SO_4) and concentrated *in vacuo*. The crude carboxylic acid was used directly without further purification.

A solution of the crude carboxylic acid in anhydrous DMF (2 mL) was treated with EDC•HCl (156.5 mg, 0.816 mmol, 10.0 equiv) and stirred at rt under Ar for 24 h. The resulting mixture was treated with D-Ala-OMe•HCl (113.5 mg, 0.813 mmol, 10.0 equiv), Et_3N (180 μL, 131 mg, 1.29 mmol, 15.9 equiv), and additional DMF (1 mL), then stirred at 70 °C under Ar for 48 h. The mixture was diluted with H_2O (8 mL) and extracted with EtOAc (3 × 10 mL). The combined organic layers were dried (Na_2SO_4) and concentrated *in vacuo*. Flash chromatography (12 mL of SiO_2, 0–1.5% MeOH in CH_2Cl_2 gradient elution) afforded **13** (24.6 mg, 0.0464 mmol, 57% from **12**) as a yellow film: $[\alpha]^{25}_{D}$ +15.1 (*c* 0.6, $CHCl_3$); ^1H NMR ($CDCl_3$, 500 MHz, mixture of rotamers) δ 7.64 and 7.59 (2 br s, 1H), 6.91 and 6.83 (br s and d, *J* = 6.2 Hz, 1H), 5.47 (br s, 1H), 4.60 (q, *J* = 7.1 Hz, 1H), 4.10 and 4.04 (2d, *J* = 5.9 and 5.9 Hz, 1H), 3.74 and 3.73 (2s, 3H), 2.07 and 2.04 (2s, 3H), 1.78 (s, 3H), 1.45 and 1.44 (2s, 9H), 1.38 (d, *J* = 6.9 Hz, 3H), 1.34 (s, 3H), 1.29 and 1.26 (2s, 3H), 0.98 (t, *J* = 7.9 Hz, 9H), 0.67 (q, *J* = 7.8 Hz, 6H); ^{13}C NMR ($CDCl_3$, 125 MHz, mixture of rotamers) δ 173.33 and 173.27, 169.7 and 169.5, 165.6 and 165.3, 139.5 and 138.6, 129.2 and 127.6, 124.0 and 123.6, 80.3, 75.5, 63.9, 52.3 and 52.2, 48.22 and 48.17, 34.4, 28.2 (3C), 27.3 and 27.2, 26.7, 20.6 and 20.5, 17.9 and 17.8, 6.9 (3C), 6.5 (3C); IR (film) ν_{max} 3257, 2954, 2876, 1697, 1513, 1366, 1165, 1052 cm^{-1}; HRMS (ESI) *m/z* 530.3249 (MH^+, $C_{25}H_{47}N_3O_7SiH^+$ requires 530.3256).

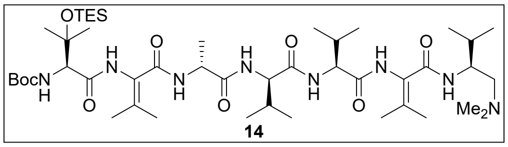

14

***tert*-Butyl ((4*S*,10*S*,13*R*,16*R*,22*S*)-25,25-Diethyl-4,10,13-triisopropyl-2,16,23,23-tetramethyl-6,9,12,15,18,21-hexaoxo-7,19-di(propan-2-ylidene)-24-oxa-2,5,8,11,14,17,20-heptaaza-25-silaheptacosan-22-yl)carbamate (14).** A

solution of tetrapeptide **8**[2] (6.7 mg, 0.013 mmol) in anhydrous CH_2Cl_2 (750 μL) at 0 °C under Ar was treated with HCl (4.0 M in dioxane, 60 μL, 0.24 mmol, 19 equiv). The resulting mixture was stirred at rt for 1.5 h, then treated with Et_3N (150 μL, 109 mg, 1.08 mmol, 84 equiv) and concentrated *in vacuo*. The crude amine was used directly in the coupling with **7** without further purification.

A solution of tripeptide **13** (9.0 mg, 0.017 mmol) in *t*-BuOH (750 μL) and H_2O (250 μL) was treated with LiOH•H_2O (4.1 mg, 0.098 mmol, 5.8 equiv), then stirred at rt for 5 h. The resulting mixture was acidified to pH 4~5 by the addition of 1 N HCl, diluted with H_2O (2 mL), and extracted with CH_2Cl_2 (3 × 5 mL). The combined organic layers were dried (Na_2SO_4) and concentrated *in vacuo*. The crude carboxylic acid **7** was used directly without further purification.

A solution of crude carboxylic acid **7** (ca. 0.017 mmol. 1.3 equiv) in anhydrous THF (500 μL) at 0 °C under Ar was treated with HOBt (ca. 20% H_2O content, 4.8 mg, 0.028 mmol, 2.2 equiv) and EDC•HCl (5.6 mg, 0.029 mmol, 2.3 equiv). The resulting mixture was stirred at 0 °C under Ar for 20 min, then treated with a solution of the crude tetrapeptide amine (ca. 0.013 mmol) in anhydrous THF (500 μL) and stirred at rt for 15 h. The reaction was quenched by the addition of sat aq $NaHCO_3$ (1 mL) and extracted with $CHCl_3$ (5 × 3 mL). The combined organic layers were dried (Na_2SO_4) and concentrated *in vacuo*. Flash chromatography (15 mL of SiO_2, 0–3% MeOH in $CHCl_3$ with 1% Et_3N gradient elution) afforded heptapeptide **14** (11.2 mg, 0.0121 mmol, 95%) as a white film: $[\alpha]^{25}_D$ +88 (*c* 0.98, $CHCl_3$); ^1H NMR ($CDCl_3$, 500 MHz, data for major rotamer) δ 9.87 (br s, 2H), 8.77 (br s, 2H), 8.02 (br s, 2H), 5.59 (br s, 1H), 4.22–4.18 (m, 2H), 4.05 (br s, 1H), 3.62–3.52 (m, 1H), 3.49–3.39 (m, 1H), 3.03 (s, 3H), 2.95 (s, 3H), 2.41–2.30 (m, 5H), 1.95 (s, 3H), 1.91 (s, 3H), 1.75 (s, 3H), 1.73 (s, 3H), 1.35 (s, 9H), 1.30 (s, 3H), 1.26–1.21 (m, 3H), 1.18 (s,

3H), 1.01 (s, 3H), 0.99 (s, 3H), 0.95–0.85 (m, 15H), 0.82 (s, 3H), 0.80 (s, 3H), 0.59 (q, J = 7.6 Hz, 6H); ^{13}C NMR ($CDCl_3$, 125 MHz, data for major rotamer) δ 176.1, 175.4, 174.1, 173.4, 173.1, 166.8, 157.2, 129.1, 128.2, 124.1, 115.5, 79.8, 73.4, 65.0, 61.0, 59.3, 49.3, 46.4, 42.8 (2C), 31.1, 30.9, 29.5, 29.2, 28.4 (3C), 27.3, 26.9, 21.1, 20.9, 20.7, 20.3, 19.6, 19.5, 19.4, 19.13, 19.09, 18.9, 17.1, 7.0 (3C), 6.5 (3C); IR (film) ν_{max} 3304, 2964, 2936, 2876, 1660, 1653, 1526, 1390, 1369, 1309, 1240, 1169, 1053, 1008 cm^{-1}; HRMS (ESI) *m/z* 923.6363 (MH$^+$, $C_{46}H_{86}N_8O_9SiH^+$ requires 923.6360).

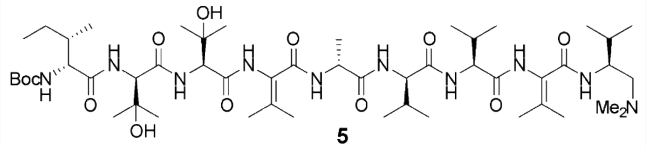

5

***tert*-Butyl ((4*S*,10*S*,13*R*,16*R*,22*S*,25*R*,28*R*,29*S*)-22,25-bis(2-hydroxypropan-2-yl)-4,10,13-triisopropyl-2,16,29-trimethyl-6,9,12,15,18,21,24,27-octaoxo-7,19-di(propan-2-ylidene)-2,5,8,11,14,17,20,23,26-nonaazahentriacontan-28-yl)carbamate (5)**. A solution of ethyl (*R*)-2-((2*R*,3*S*)-2-((*tert*-butoxycarbonyl)amino)-3-methylpentanamido)-3-hydroxy-3-methylbutanoate[2] (7.9 mg, 0.021 mmol) in *t*-BuOH (750 μL) and H_2O (250 μL) at 0 °C was treated with LiOH•H_2O (4.4 mg, 0.10 mmol, 5.0 equiv), then stirred at rt for 4 h. The resulting mixture was acidified to pH 4~5 by the addition of 1 N HCl, diluted with H_2O (2 mL), and extracted with $CHCl_3$ (5 × 5 mL). The combined organic layers were dried (Na_2SO_4) and concentrated *in vacuo*. The crude carboxylic acid **6** was used directly without further purification.

A solution of heptapeptide **14** (18.0 mg, 0.0195 mmol) in anhydrous CH_2Cl_2 (1 mL) at 0 °C under Ar was treated with HCl (4.0 M in dioxane, 160 μL, 0.64 mmol, 33 equiv). The resulting mixture was stirred at rt for 4 h, then concentrated *in vacuo*. The crude amine was used directly in the coupling with **6** without further purification.

A solution of crude carboxylic acid **6** (ca. 0.021 mmol. 1.1 equiv) in anhydrous CH_2Cl_2 (1 mL) at 0 °C under Ar was treated with HOBt (ca. 20% H_2O content, 7.2 mg, 0.043 mmol, 2.2 equiv) and EDC•HCl (11.3 mg, 0.059 mmol, 3.0 equiv). The resulting mixture was stirred at 0 °C under Ar for 20 min, then treated with a solution of the crude heptapeptide amine (ca. 0.0195 mmol) in anhydrous CH_2Cl_2 (1 mL) and stirred at rt for 15 h. The reaction was quenched by the addition of sat aq $NaHCO_3$ (2.0 mL) and extracted with $CHCl_3$ (5 × 5 mL). The combined organic layers were dried (Na_2SO_4) and concentrated *in vacuo*. Flash chromatography (15 mL of SiO_2, 0–7% MeOH in $CHCl_3$ with 1% Et_3N gradient elution) afforded nonapeptide **5** (16.2 mg, 0.0156 mmol, 80% from heptapeptide **14**) as a white film: $[\alpha]^{25}_D$ +67 (*c* 1.1, $CHCl_3$); ^{1}H NMR (DMSO-d_6, 500 MHz, data for major rotamer) δ 9.70 (br s, 1H), 9.21 (s, 1H), 8.07 (d, *J* = 7.0 Hz, 1H), 7.87–7.77 (m, 4H), 7.70 (d, *J* = 9.0 Hz, 1H), 6.92 (d, *J* = 9.2 Hz, 1H), 5.06 (s, 1H), 4.93 (s, 1H), 4.43 (d, *J* = 9.1 Hz, 1H), 4.37 (d, *J* = 8.0 Hz, 1H), 4.25–4.15 (m, 2H), 4.13 (t, *J* = 7.2 Hz, 1H), 4.05–3.95 (m, 2H), 3.27–3.14 (m, 2H), 2.84 (br s, 3H), 2.72 (br s, 3H), 2.08–2.00 (m, 2H), 1.98 (s, 3H), 1.92 (s, 3H), 1.80–1.68 (m, 4H), 1.67 (s, 3H), 1.66 (s, 3H), 1.36 (s, 9H), 1.21 (s, 3H), 1.17 (d, *J* = 7.0 Hz, 3H), 1.14 (s, 3H), 1.11 (s, 3H), 1.06 (s, 3H), 0.91–0.87 (m, 9H), 0.85–0.79 (m, 12H), 0.74 (d, *J* = 6.8 Hz, 3H); ^{13}C NMR (DMSO-d_6, 125 MHz, data for major rotamer) δ 172.7, 172.3, 171.9, 171.8, 170.1, 169.8, 166.4, 165.1, 156.0, 138.7, 134.8, 125.2, 124.3, 79.7, 78.6, 71.9, 60.7, 60.2, 59.4, 58.7, 58.6, 49.9, 49.2, 45.2 (2C), 42.0, 36.9, 31.8, 31.0, 30.1, 29.5, 28.6 (3C), 28.4, 28.3, 26.3, 25.7, 21.9, 21.3, 20.7, 19.75, 19.72, 19.67, 19.63, 19.0, 18.6, 18.3, 17.6, 15.0, 12.0; IR (film) ν_{max} 3294, 2966, 2932, 2876, 2255, 2126, 1660, 1530, 1467, 1307, 1238, 1171, 1027, 1008 cm^{-1}; HRMS (ESI) *m/z* 1037.6970 (MH^+, $C_{51}H_{92}N_{10}O_{12}H^+$ requires 1037.6969).

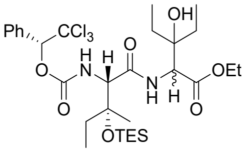

17a

Ethyl 3-Ethyl-3-hydroxy-2-((2*S*,3*R*)-3-methyl-2-((((*R*)-2,2,2-trichloro-1-phenylethoxy)carbonyl)amino)-3-((triethylsilyl)oxy)pentanamido)pentanoate (17a). A suspension of **15**[2] (127.5 mg, 0.2357 mmol) and Me_3SnOH (171.1 mg, 0.9462 mmol, 4.0 equiv) in hexanes (8 mL, pretreated with Na_2SO_4 for 6 h) was stirred at 60 °C under Ar for 72 h. The mixture was concentrated *in vacuo*, and the residue was treated with Et_2O (3 mL). The mixture was filtered through Celite, (washed with 10 mL of Et_2O), and the filtrate was concentrated *in vacuo* to afford the crude acid as a colorless oil that was used directly in the next step without further purification.

The crude acid prepared above was dissolved in anhydrous CH_2Cl_2 (2 mL), cooled to 0 °C under Ar, then treated with amine **16a**[4] (67.2 mg, 0.355 mmol, 1.5 equiv), HOBt (ca. 20% H_2O content, 60.0 mg, 0.355 mmol, 1.5 equiv), and EDC•HCl (67.7 mg, 0.353 mmol, 1.5 equiv). The resulting mixture was stirred at 0 °C to rt under Ar for 18 h. The reaction was quenched by the addition of sat aq $NaHCO_3$ (1 mL), the layers were separated, and the aqueous layer was extracted with CH_2Cl_2 (3 × 4 mL). The combined organic layers were dried (Na_2SO_4) and concentrated *in vacuo*. Flash chromatography (44 mL of SiO_2, 0–1.5% MeOH in CH_2Cl_2 gradient elution) afforded **17a** (142.4 mg, 0.2081 mmol, 88%) as a white film that was a 1:1 mixture of diastereomers: ^{1}H NMR ($CDCl_3$, 500 MHz, minor rotamers present, data for major rotamer of each diastereomer) δ 7.61 (d, *J* = 6.8 Hz, 2H), 7.44–7.35 (m, 3H), 7.25 and 7.09 (2d, *J* = 8.6 and 8.7 Hz, 1H), 6.28 and 6.26 (2s, 1H), 6.01 and 5.92 (2d, *J* = 8.0 and 7.5 Hz, 1H), 4.63 and 4.56 (2d, *J* = 8.8 and 8.7 Hz 1H), 4.28–4.15 (m, 3H), 2.45 and 2.40 (2 br s, 1H), 1.63–1.45 (m, 6H), 1.31 (t, *J* = 7.2 Hz, 3H),

[4] J. Jiang, S. Luo and S. L. Castle, *Tetrahedron Lett.*, 2015, **56**, 3311.

1.25 and 1.23 (2s, 3H), 1.02–0.92 (m, 9H), 0.90–0.79 (m, 9H), 0.75–0.63 (m, 6H); ^{13}C NMR ($CDCl_3$, 125 MHz) δ 172.04 and 171.98, 170.1 and 169.8, 154.7 and 154.5, 133.52 and 133.45, 130.2, 129.8 (2C), 128.0 (2C), 99.7, 83.71 and 83.67, 78.7, 78.2, 76.4 and 76.1, 62.1 and 61.7, 57.4 and 57.1, 32.8 and 31.8, 28.74 and 28.69, 26.8 and 26.6, 24.11 and 24.08, 14.3, 8.9 and 8.7, 7.84 and 7.79, 7.7, 7.3 (3C), 6.8 (3C); IR (film) ν_{max} 3353, 3286, 2964, 2878, 2359, 1732, 1661, 1505, 1377, 1200, 1067 cm^{-1}; HRMS (ESI) *m/z* 683.2445 (MH^+, $C_{30}H_{49}Cl_3N_2O_7SiH^+$ requires 683.2447).

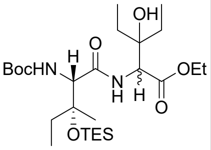

18a

Ethyl 2-((2*S*,3*R*)-2-((*tert*-Butoxycarbonyl)amino)-3-methyl-3-((triethylsilyl)oxy)pentanamido)-3-ethyl-3-hydroxypentanoate (18a). A suspension of carbamate **17a** (142.4 mg, 0.2081 mmol) in THF–sat aq $NaHCO_3$ (2:1, 2.3 mL) was treated sequentially with 10% Pd/C (22.1 mg, 0.16 wt equiv) and Boc_2O (49.9 mg, 0.229 mmol, 1.1 equiv). The resulting mixture was stirred at rt under H_2 (200 psi) for 24 h, diluted with H_2O (3 mL), and extracted with EtOAc (3 × 7 mL). The combined organic layers were dried (Na_2SO_4) and concentrated *in vacuo*. Flash chromatography (44 mL of SiO_2, 0–1.5% MeOH in CH_2Cl_2 gradient elution) afforded **18a** (104.0 mg, 0.1952 mmol, 94%) as a white film that was a 1:1 mixture of diastereomers: ^1H NMR ($CDCl_3$, 500 MHz) δ 7.39 and 7.34 (2 br s, 1H), 7.02 (br s, 1H), 5.39 and 5.31 (d and br s, *J* = 7.2 Hz, 1H), 4.60 and 4.53 (2d, *J* = 8.7 Hz and 8.8 Hz, 1H), 4.21–4.11 (m, 3H), 2.50 and 2.48 (2 br s, 1H), 1.55–1.41 (m, 6H), 1.40 and 1.39 (2s, 9H), 1.29–1.22 (m, 5H), 0.96–0.80 (m, 18H), 0.69–0.59 (m, 6H); ^{13}C NMR ($CDCl_3$, 125 MHz) δ 172.1, 171.0 and 170.7, 156.2 and 155.9, 80.0 and 79.8, 78.9 and 78.2, 76.5 and 76.1, 74.3, 61.5, 57.2 and 56.8, 33.0 and 31.9, 28.6, 28.4 (3C), 26.7, 24.2, 14.3, 8.9 and 8.7, 7.8 and 7.7, 7.74, 7.3 (3C), 6.9 and 6.8 (3C);

IR (film) ν_{max} 3359, 2968, 2878, 2360, 2342, 1722, 1662, 1505, 1367, 1166, 1023 cm^{-1}; HRMS (ESI) *m/z* 533.3614 (MH^+, $C_{26}H_{52}N_2O_7SiH^+$ requires 533.3617).

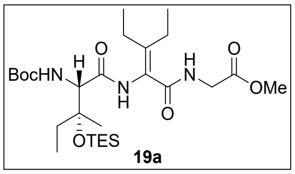

Methyl (2-((2*S*,3*R*)-2-((*tert*-Butoxycarbonyl)amino)-3-methyl-3-((triethylsilyl)oxy)pentanamido)-3-ethylpent-2-enoyl)glycinate (19a). A solution of ester **18a** (45.0 mg, 0.0845 mmol) in *t*-BuOH–H_2O (3:1, 3 mL) was treated with LiOH•H_2O (17.7 mg, 0.422 mmol, 5.0 equiv) at 0 °C, then stirred at rt for 5 h. The resulting mixture was acidified to pH 4~5 by the addition of 1 N HCl, diluted with H_2O (2 mL), and extracted with CH_2Cl_2 (3 × 5 mL). The combined organic layers were dried (Na_2SO_4) and concentrated *in vacuo*. The crude carboxylic acid was used directly without further purification.

A solution of the crude carboxylic acid in anhydrous DMF (3 mL) was treated with EDC•HCl (162.0 mg, 0.8451 mmol, 10 equiv) under Ar and stirred at rt for 24 h. The resulting mixture was treated with Gly-OMe•HCl (106.1 mg, 0.8451 mmol, 10 equiv), Et_3N (190 μL, 138 mg, 1.36 mmol, 16 equiv), and additional anhydrous DMF (1 mL), then stirred under Ar at 80 °C for 48 h. The solution was cooled to rt, diluted with EtOAc (10 mL), and washed with brine (10 × 10 mL) to remove the DMF. The combined organic layers were dried (Na_2SO_4) and concentrated *in vacuo*. Flash chromatography (20 mL of SiO_2, 0–1.5% MeOH in CH_2Cl_2 gradient elution) afforded **19a** (23.0 mg, 0.0412 mmol, 49%) as a pale yellow oil: $[\alpha]^{25}_D$ –3.3 (*c* 1.7, $CHCl_3$); ^1H NMR ($CDCl_3$, 500 MHz) δ 7.41 (br s, 1H), 7.01 (br s, 1H), 5.31 (br s, 1H), 4.05–3.98 (m, 2H), 3.91 (dd, *J* = 12.5, 5.5 Hz, 1H), 3.66 (s, 3H), 2.41–2.32 (m, 2H), 2.06 (q, *J* = 7.6 Hz, 2H), 1.64–1.58 (m, 2H), 1.37 (s, 9H), 1.29 (s, 3H), 1.03 (t, *J* = 7.4 Hz, 3H), 0.97–0.87 (m, 12H), 0.85–0.78 (m, 3H), 0.60 (q, *J* = 7.9 Hz, 6H); ^{13}C NMR ($CDCl_3$, 125 MHz) δ 170.5, 170.2, 166.3, 156.4, 150.2, 123.0, 80.3, 78.1,

61.6, 52.2, 41.3, 32.7, 28.3 (3C), 28.0, 24.23, 24.18, 13.2, 12.1, 8.8, 7.1 (3C), 6.7 (3C); IR (film) ν_{max} 3335, 2962, 2877, 2359, 1682, 1505, 1367, 1169, 1066, 1008 cm^{-1}; HRMS (ESI) *m/z* 538.3570 (MH^+, $C_{27}H_{51}N_3O_7SiH^+$ requires 538.3569).

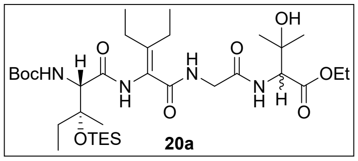

20a

Ethyl (5*R*,6*S*)-6-((*tert*-Butoxycarbonyl)amino)-3,3,5-triethyl-15-(2-hydroxypropan-2-yl)-5-methyl-7,10,13-trioxo-9-(pentan-3-ylidene)-4-oxa-8,11,14-triaza-3-silahexadecan-16-oate (20a). A solution of tripeptide **19a** (10.6 mg, 0.0190 mmol) in *t*-BuOH (600 µL) was treated with LiOH•H_2O (4.3 mg, 0.10 mmol, 5.4 equiv) and H_2O (200 µL), then stirred at rt for 3 h. The resulting mixture was acidified to pH 4~5 by the addition of 1 N HCl, diluted with H_2O (2 mL), and extracted with EtOAc (2 × 3 mL). The combined organic layers were dried (Na_2SO_4) and concentrated *in vacuo*. The crude carboxylic acid was used directly without further purification.

The crude acid was dissolved in anhydrous CH_2Cl_2 (2.0 mL), cooled to 0 °C under Ar, then treated with amine **10** (4.9 mg, 0.030 mmol, 1.6 equiv), HOBt (ca. 20% H_2O content, 4.8 mg, 0.028 mmol, 1.5 equiv) and EDC•HCl (5.8 mg, 0.030 mmol, 1.6 equiv). The resulting mixture was stirred at rt under Ar for 15 h. The reaction was quenched by the addition of sat aq $NaHCO_3$ (1 mL), the layers were separated, and the aqueous layer was extracted with CH_2Cl_2 (4 × 4 mL). The combined organic layers were dried (Na_2SO_4) and concentrated *in vacuo*. Flash chromatography 23 mL of SiO_2, 0–4% MeOH in CH_2Cl_2 gradient elution) afforded **20a** (10.5 mg, 0.0153 mmol, 80%) as a colorless oil that was a 1:1 mixture of diastereomers: ^{1}H NMR ($CDCl_3$, 500 MHz) δ 7.64–7.50 (m, 2H), 7.34–7.26 (m, 1H), 7.01 and 6.94 (2 br s, 1H), 5.48 (br s, 1H), 4.54 and 4.51 (2d, *J* = 9.4 and 9.2 Hz, 1H), 4.20–4.05 (m, 3H), 4.02–3.98 and 3.94–3.90 (2m, 1H), 3.73–3.64

(m, 1H), 2.44–2.28 (m, 2H), 2.12–2.05 (m, 2H), 1.62–1.54 (m, 2H), 1.38 and 1.37 (2s, 9H), 1.30–1.26 (m, 3H), 1.23–1.17 (m, 9H), 1.04–1.00 (m, 3H), 0.98 (t, $J = 7.6$ Hz, 3H), 0.92 (t, $J = 7.9$ Hz, 9H), 0.86–0.78 (m, 3H), 0.63–0.57 (m, 6H); ^{13}C NMR ($CDCl_3$, 125 MHz) δ 171.3, 170.5, 170.4, 159.8, 156.7, 150.9, 130.0, 81.0, 77.8, 72.0, 62.2 and 62.0, 61.2 and 61.0, 60.5, 43.2, 32.8, 28.3 (3C), 27.3, 26.8, 24.4, 24.3, 24.2, 14.2 and 14.1, 13.2, 12.1, 8.8, 7.1 (3C), 6.7 (3C); IR (film) ν_{max} 3371, 2911, 2868, 2214, 1750, 1631, 1590, 1389, 1311, 1298, 1170 cm^{-1}; HRMS (ESI) *m/z* 687.4361 (MH^+, $C_{33}H_{62}N_4O_9SiH^+$ requires 687.4359).

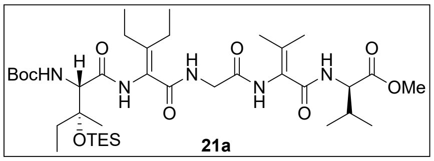

Methyl (2-(2-(2-((2*S*,3*R*)-2-((*tert*-Butoxycarbonyl)amino)-3-methyl-3-((triethylsilyl)oxy)pentanamido)-3-ethylpent-2-enamido)acetamido)-3-methylbut-2-enoyl)-D-valinate (21a). A solution of ester **20a** (13.8 mg, 0.0201 mmol) in *t*-BuOH-H_2O (3:1, 1 mL) was treated with LiOH•H_2O (4.3 mg, 0.10 mmol, 5.1 equiv), then stirred at rt for 5 h. The resulting mixture was acidified to pH 4~5 by the addition of 1 N HCl, diluted with H_2O (2 mL), and extracted with CH_2Cl_2 (3 × 4 mL). The combined organic layers were dried (Na_2SO_4) and concentrated *in vacuo*. The crude carboxylic acid was used directly without further purification.

A solution of the crude carboxylic acid in anhydrous DMF (1 mL) was treated with EDC•HCl (39.0 mg, 0.203 mmol, 10.1 equiv) and stirred at rt under Ar for 24 h, at which point the azlactone intermediate was formed according to MS. The resulting mixture was treated with D-Val-OMe•HCl (34.1 mg, 0.203 mmol, 10.1 equiv), Et_3N (46 μL, 33 mg, 0.33 mmol, 16 equiv), and additional anhydrous DMF (1 mL), then stirred at 80 °C under Ar for 48 h. The solution was cooled to rt, diluted with EtOAc (5 mL), and washed with brine (10 × 8 mL) to remove the DMF. The combined organic layers were dried (Na_2SO_4) and concentrated *in vacuo*. Flash chromatography

(10 mL of SiO_2, 0–10 % MeOH in CH_2Cl_2 gradient elution) afforded **21a** (7.1 mg, 0.0094 mmol, 47%) as a yellow oil: $[\alpha]^{25}_{D}$ –10.9 (*c* 1.1, $CHCl_3$); ^1H NMR ($CDCl_3$, 500 MHz) δ 8.48 (br s, 1H), 7.68 (br s, 1H), 6.94 (d, *J* = 8.0 Hz, 1H), 5.44 (br s, 1H), 5.35 (br s, 1H), 4.47 (dd, *J* = 8.0, 5.8 Hz, 1H), 4.19 (dd, *J* = 16.9, 6.4 Hz, 1H), 4.00 (d, *J* = 5.1 Hz, 1H), 3.81–3.73 (m, 1H), 3.71 (s, 3H), 2.53–2.35 (m, 2H), 2.25–2.12 (m, 2H), 2.12 (s, 3H), 2.09–2.00 (m, 1H), 1.81 (s, 3H), 1.70–1.62 (m, 2H), 1.45 (s, 9H), 1.33 (s, 3H), 1.10 (t, *J* = 7.5 Hz, 3H), 1.04 (t, *J* = 7.6 Hz, 3H), 1.01–0.96 (m, 12H), 0.94–0.82 (m, 6H), 0.68 (q, *J* = 7.9 Hz, 6H); ^{13}C NMR ($CDCl_3$, 125 MHz) δ 173.2, 171.1, 169.0, 166.2, 165.9, 154.5, 148.0, 141.8, 130.2, 130.0, 81.2, 77.2 (obscured by solvent), 62.1, 58.0, 52.1, 44.1, 32.1, 29.5, 28.5 (3C), 27.4, 25.7, 24.6, 22.9, 21.0, 19.3, 18.5, 13.4, 12.4, 8.9, 7.3 (3C), 6.9 (3C); IR (film) ν_{max} 3312, 2965, 2983, 2855, 1698, 1623, 1510, 1359, 1168 cm^{-1}; HRMS (ESI) *m/z* 754.4783 (MH^+, $C_{37}H_{67}N_5O_9SiH^+$ requires 754.4781).

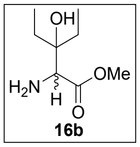

16b

Methyl 2-Amino-3-ethyl-3-hydroxypentanoate (16b). A solution of benzyl ((methylsulfonyl)oxy)carbamate[5] (1.80 g, 7.34 mmol, 1.5 equiv) in CH_3CN (50 mL) at rt was treated with OsO_4 (4 wt % solution in H_2O, 3.1 mL, 0.49 mmol, 0.1 equiv), stirred for 15 min, then treated with methyl 3-ethylpent-2-enoate[6] (685.8 mg, 4.823 mmol) and H_2O (5 mL). The resulting mixture was stirred at 47 °C for 72 h, then quenched by the addition of sat aq $K_2S_2O_5$ (15 mL), diluted with H_2O (15 mL), and extracted with EtOAc (3 × 35 mL). The combined organic layers were washed with sat aq $NaHCO_3$ (2 × 35 mL) and brine (35 mL), dried (Na_2SO_4), and concentrated *in vacuo*. Flash chromatography (85 mL of SiO_2, 0–25% EtOAc in hexanes gradient

[5] L. Qin, Z. Zhou, J. Wei, T. Yan and H. Wen, *Synth. Commun.*, 2010, **40**, 642.

[6] (a) R. Ciabatti, S. Maffioli, A. Checchia, G. Romano', G. Candiani and G. Panzone, WO 2003076460 A1, 2003. (b) V. Rawat, P. V. Chouthaiwale, V. B. Chavan, G. Suryavanshi, and A. Sudalai, *Tetrahedron Lett.*, 2010, **51**, 6565.

elution) afforded methyl 2-(((benzyloxy)carbonyl)amino)-3-ethyl-3-hydroxypentanoate (537.2 mg, 1.736 mmol, 36%) as a light yellow oil: ^{1}H NMR ($CDCl_3$, 500 MHz) δ 7.39–7.31 (m, 5H), 5.66 (d, J = 9.2 Hz, 1H), 5.12 (s, 2H), 4.38 (d, J = 9.4 Hz, 1H), 3.78 (s, 3H), 2.27 (br s, 1H), 1.62–1.40 (m, 4H), 0.93 (t, J = 7.4 Hz, 3H), 0.87 (t, J = 7.5 Hz, 3H); ^{13}C NMR ($CDCl_3$, 125 MHz) δ 173.0, 156.4, 136.3, 128.7 (2C), 128.4, 128.3 (2C), 76.3, 67.4, 58.6, 52.5, 28.6, 26.6, 7.8, 7.7; IR (film) ν_{max} 3432, 3033, 2970, 2950, 2883, 1723, 1514, 1455, 1378, 1209, 1051 cm^{-1}; HRMS (ESI) *m/z* 310.1651 (MH^+, $C_{16}H_{23}NO_5H^+$ requires 310.1649).

A solution of methyl 2-(((benzyloxy)carbonyl)amino)-3-ethyl-3-hydroxypentanoate (537.2 mg, 1.736 mmol) in MeOH (10 mL) at rt under Ar was treated with 10% Pd/C (53.3 mg, 0.10 wt equiv). The resulting suspension was stirred at rt under H_2 (550 psi) for 72 h, then filtered through Celite. The Celite pad was rinsed with MeOH, and the filtrate was concentrated *in vacuo* to afford **16b** (154 mg, 0.879 mmol, 51%) as a light yellow oil. The crude amine was used directly without further purification.

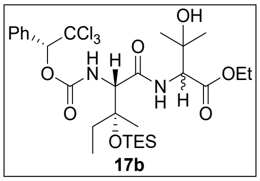

17b

Ethyl 3-Hydroxy-3-methyl-2-((2*S*,3*R*)-3-methyl-2-((((*R*)-2,2,2-trichloro-1-phenylethoxy)carbonyl)amino)-3-((triethylsilyl)oxy)pentanamido)butanoate (17b). A suspension of **15**[4] (200 mg, 0.370 mmol) and Me_3SnOH (401 mg, 2.22 mmol, 6.0 equiv) in hexane (25 mL, pretreated with Na_2SO_4 for 6 h) was stirred at 70 °C under Ar for 72 h. The mixture was concentrated *in vacuo*, and the residue was treated with Et_2O (10 mL). The mixture was filtered through Celite, (washed with 60 mL of Et_2O), and the filtrate was concentrated *in vacuo* to afford the crude acid as a colorless oil that was used directly in the next step without further purification.

The crude acid prepared above (ca. 0.370 mmol) was dissolved in anhydrous CH_2Cl_2 (2 mL), cooled to 0 °C under Ar, then treated with amine **10**[2,3] (77.6 mg, 0.481 mmol, 1.3 equiv), HOBt (ca. 20% H_2O content, 100 mg, 0.592 mmol, 1.6 equiv), and EDC•HCl (107 mg, 0.558 mmol, 1.5 equiv). The resulting mixture was stirred at 0 °C under Ar for 3 h. The reaction was quenched by the addition of sat aq $NaHCO_3$ (1 mL), the layers were separated, and the aqueous layer was extracted with CH_2Cl_2 (6 × 4 mL). The combined organic layers were dried (Na_2SO_4) and concentrated *in vacuo*. Flash chromatography (88 mL of SiO_2, 0–1.5% MeOH in CH_2Cl_2 gradient elution) afforded **17b** (203 mg, 0.309 mmol, 84%) as a colorless oil that was a 1:1 mixture of diastereomers: ^{1}H NMR ($CDCl_3$, 500 MHz, minor rotamers present, data for major rotamer of each diastereomer) δ 7.61 (d, *J* = 6.4 Hz, 2H), 7.45–7.35 (m, 3H), 7.28 and 7.13 (2d (one is partially obscured by solvent), *J* = 8.5 Hz, 1H), 6.28 and 6.26 (2s, 1H), 6.01 and 5.93 (2d, *J* = 8.1 and 7.3 Hz, 1H), 4.55 and 4.48 (2d, *J* = 8.6 and 8.6 Hz, 1H), 4.29–4.17 (m, 3H), 2.69 (br s, 1H), 1.66–1.43 (m, 2H), 1.31 (t, *J* = 7.2 Hz, 3H), 1.30–1.22 (m, 9H), 0.99 (t, *J* = 7.9 Hz, 9H), 0.90–0.80 (m, 3H), 0.68 (q, *J* = 7.5 Hz, 6H); ^{13}C NMR ($CDCl_3$, 125 MHz) δ 171.5 and 171.3, 170.4 and 170.1, 154.7 and 154.6, 133.5 and 133.4, 130.2, 129.8 (2C), 128.1 (2C), 99.6, 83.7, 78.7 and 78.2, 72.4 and 71.8, 62.2 and 62.1, 61.8, 60.4 and 60.3, 32.8 and 31.9, 27.2 and 26.94, 26.87 and 26.7, 24.1, 14.4, 8.9 and 8.7, 7.3 (3C), 6.8 (3C); IR (film) ν_{max} 3352, 2923, 2361, 1734, 1668, 1506, 1377, 1065 cm^{-1}; HRMS (ESI) *m/z* 655.2139 (MH^+, $C_{28}H_{45}Cl_3N_2O_7SiH^+$ requires 655.2135.

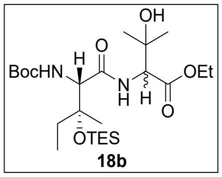

18b

Ethyl 2-((2*S*,3*R*)-2-((*tert*-Butoxycarbonyl)amino)-3-methyl-3-((triethylsilyl)oxy)pentanamido)-3-hydroxy-3-methylbutanoate (18b). A suspension of carbamate **17b** (87.0 mg, 0.133 mmol) in THF–sat aq $NaHCO_3$ (2:1, 10 mL) was treated

sequentially with 10% Pd/C (43.5 mg, 0.50 wt equiv) and Boc_2O (116 mg, 0.532 mmol, 4.0 equiv). The resulting mixture was stirred at rt under H_2 (600 psi) for 15 h, diluted with H_2O (1 mL) and sat aq $NaHCO_3$ (1 mL), and extracted with EtOAc (5 × 5 mL). The combined organic layers were dried (Na_2SO_4) and concentrated *in vacuo*. Flash chromatography (98 mL of SiO_2, 0–1.5% MeOH in CH_2Cl_2 gradient elution) afforded **18b** (64.0 mg, 0.127 mmol, 96%) as a colorless oil that was a 1:1 mixture of diastereomers: ^{1}H NMR ($CDCl_3$, 500 MHz) δ 7.39 and 7.08 (2 br s, 1H), 5.42 and 5.36 (d and br s, J = 7.2 Hz, 1H), 4.56 and 4.49 (2d, J = 8.8 Hz and 8.6 Hz, 1H), 4.28–4.17 (m, 2.5H), 4.15–4.09 (m, 0.5H), 2.84 (br s, 1H), 1.69–1.60 and 1.58–1.46 (2m, 2H), 1.45 (s, 9H), 1.35–1.27 (m, 6H), 1.26 (s, 3H), 1.25 (s, 3H), 1.01–0.94 (m, 9H), 0.92–0.84 (m, 3H), 0.74–0.62 (m, 6H); ^{13}C NMR ($CDCl_3$, 125 MHz) δ 172.7, 171.0 and 170.9, 156.4 and 156.2, 80.6 and 80.5, 74.0 and 73.9, 72.4 and 72.2, 61.9 and 61.7, 60.2 and 60.0, 59.1 and 58.6, 30.72 and 30.66, 28.5 (3C), 27.0, 26.8 and 26.7, 24.2 and 23.8, 14.3, 8.0, 7.3 and 7.0 (3C), 6.8 and 6.6 (3C); IR (film) ν_{max} 3371, 2923, 2359, 1718, 1652, 1506, 1457, 1368, 1164 cm^{-1}; HRMS (ESI) *m/z* 505.3299 (MH^+, $C_{24}H_{48}N_2O_7SiH^+$ requires 505.3309).

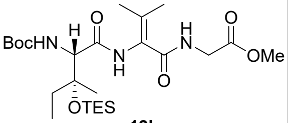

19b

Methyl (2-((2*S*,3*R*)-2-((*tert*-Butoxycarbonyl)amino)-3-methyl-3-((triethylsilyl)oxy)pentanamido)-3-methylbut-2-enoyl)glycinate (19b). A solution of ester **18b** (116.0 mg, 0.2298 mmol) in *t*-BuOH (800 μL) was treated with LiOH•H_2O (48.0 mg, 1.14 mmol, 5.0 equiv) and H_2O (200 μL) at 0 °C, then stirred at 0 °C to rt for 4 h. The resulting mixture was acidified to pH 4~5 by the addition of 1 N HCl, diluted with H_2O (2 mL), and extracted with EtOAc (3 × 5 mL). The combined organic layers were dried (Na_2SO_4) and concentrated *in vacuo*. The crude carboxylic acid was used directly in the next step without further purification.

A solution of the crude carboxylic acid in anhydrous CH_2Cl_2 (2 mL) was treated with EDC•HCl (441.0 mg, 2.300 mmol, 10.0 equiv) and stirred at rt under Ar for 24 h. The resulting mixture was treated with brine (4 mL) and extracted with CH_2Cl_2 (3 × 5 mL). The combined organic layers were dried (Na_2SO_4) and concentrated *in vacuo*. The crude azlactone was used directly in the next step without further purification.

A solution of the crude azlactone in anhydrous DMF (2 mL) was treated with DMAP (60.0 mg, 0.491 mmol, 2.1 equiv, Gly-OMe•HCl (290.0 mg, 2.310 mmol, 10.1 equiv), and Et_3N (480 μL, 348 mg, 3.44 mmol, 15.0 equiv). The resulting solution was stirred at 80 °C under Ar for 72 h. The solution was diluted with H_2O (8 mL) and extracted with EtOAc (3 × 10 mL). The combined organic layers were washed with brine (2 × 5 mL), dried (Na_2SO_4), and concentrated *in vacuo*. Flash chromatography (50 mL of SiO_2, 0–3% MeOH in CH_2Cl_2 gradient elution) afforded **19b** (87.0 mg, 0.164 mmol, 71%) as a white solid: $[\alpha]^{25}_{D}$ –3.6 (*c* 0.84, $CHCl_3$); ^{1}H NMR ($CDCl_3$, 500 MHz) δ 7.49 (s, 1H), 7.03 (br s, 1H), 5.39 (br s, 1H), 4.13–4.05 (m, 2H), 4.02 (dd, *J* = 18.0, 5.5 Hz, 1H), 3.74 (s, 3H), 2.10 (s, 3H), 1.79 (s, 3H), 1.67–1.56 (m, 2H), 1.44 (s, 9H), 1.37 (s, 3H), 0.99 (t, *J* = 7.9 Hz, 9H), 0.92 (t, *J* = 7.6 Hz, 3H), 0.68 (q, *J* = 7.9 Hz, 6H); ^{13}C NMR ($CDCl_3$, 125 MHz) δ 170.4, 170.2, 166.3, 156.7, 142.7, 123.5, 80.6, 78.3, 62.0, 52.4, 41.5, 32.9, 28.5 (3C), 24.5, 21.5, 20.9, 8.9, 7.3 (3C), 6.9 (3C); IR (film) ν_{max} 3328, 2955, 2877, 1667, 1526, 1369, 1212, 1008 cm^{-1}; HRMS (ESI) *m/z* 530.3239 (MH$^+$, $C_{25}H_{47}N_3O_7SiH^+$ requires 530.3250).

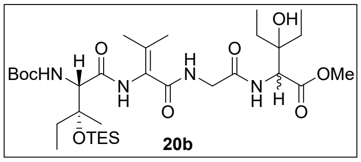

Methyl (5*R*,6*S*)-6-((*tert*-Butoxycarbonyl)amino)-3,3,5-triethyl-15-(3-hydroxypentan-3-yl)-5-methyl-7,10,13-trioxo-9-(propan-2-ylidene)-4-oxa-8,11,14-triaza-3-silahexadecan-16-oate (20b). A solution of tripeptide **19b** (20.0 mg, 0.0378 mmol) in *t*-

BuOH (800 μL) was treated with LiOH•H_2O (7.9 mg, 0.19 mmol, 5.0 equiv) and H_2O (200 μL), then stirred at rt for 4 h. The resulting mixture was neutralized to pH ~7 by the addition of 1 N HCl, diluted with H_2O (2 mL), and extracted with EtOAc (3 × 5 mL). The combined organic layers were dried (Na_2SO_4) and concentrated *in vacuo*. The crude carboxylic acid was used directly without further purification.

The crude acid was dissolved in anhydrous CH_2Cl_2 (4.0 mL), cooled to 0 °C under Ar, then treated with amine **16b** (9.9 mg, 0.056 mmol, 1.5 equiv), HOBt (ca. 20% H_2O content, 10.2 mg, 0.060 mmol, 1.6 equiv), and EDC•HCl (10.9 mg, 0.0569 mmol, 1.5 equiv). The resulting mixture was stirred at rt under Ar for 5 h. The reaction was quenched by the addition of sat aq $NaHCO_3$ (1 mL), the layers were separated, and the aqueous layer was extracted with CH_2Cl_2 (6 × 5 mL). The combined organic layers were dried (Na_2SO_4) and concentrated *in vacuo*. Flash chromatography (30 mL of SiO_2, 0–4% MeOH in CH_2Cl_2 gradient elution) afforded **20b** (18.0 mg, 0.0267 mmol, 71%) as a white film that was a 1:1 mixture of diastereomers: ^1H NMR ($CDCl_3$, 500 MHz, minor rotamers present, data for major rotamer of each diastereomer) δ 7.81 and 7.63 (2 br s, 1H), 7.63 and 7.47 (br s and d, *J* = 6.9 Hz, 1H), 7.01 and 6.96 (2 br s, 1H), 5.56 and 5.50 (2 br s, 1H), 4.75 and 4.73 (2s, 1H), 4.29–4.12 (m, 1H), 4.08 (d, *J* = 4.6 Hz, 1H), 3.95–3.81 (m, 1H), 3.73 and 3.71 (2s, 3H), 2.10 and 2.05 (2s, 3H), 1.83 and 1.81 (2s, 3H), 1.74–1.50 (m, 7H), 1.45 (s, 9H), 1.36 (s, 3H), 0.99 (t, *J* = 7.9 Hz, 9H), 0.93 and 0.92 (2t, *J* = 7.4 and 7.4 Hz, 6H), 0.84 and 0.83 (2t, *J* = 7.4 and 7.4 Hz, 3H), 0.72–0.65 (m, 6H); ^{13}C NMR ($CDCl_3$, 125 MHz) δ 172.1, 171.6 and 171.5, 171.3, 165.3 and 165.0, 157.1 and 157.0, 139.8 and 139.6, 123.8 and 123.5, 81.2 and 81.0, 78.3 and 77.9, 76.5, 62.4 and 62.2, 57.6, 52.3 and 52.1, 43.3, 33.0 and 32.7, 28.5 (3C), 27.6, 27.3 and 27.2, 24.6, 21.0, 20.8, 9.0 and 8.9, 8.1, 8.0, 7.3 (3C), 6.9 (3C); IR (film) ν_{max} 3358, 2927, 2877, 2358, 1738,

1651, 1511, 1463, 1368, 1300, 1242, 1170 cm^{-1}; HRMS (ESI) *m/z* 673.4229 (MH^+, $C_{32}H_{60}N_4O_9SiH^+$ requires 673.4208).

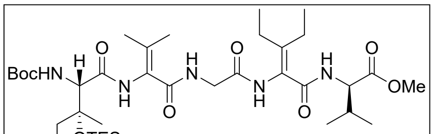

21b

Methyl (2-(2-(2-((2*S*,3*R*)-2-((*tert*-Butoxycarbonyl)amino)-3-methyl-3-((triethylsilyl)oxy)pentanamido)-3-methylbut-2-enamido)acetamido)-3-ethylpent-2-enoyl)-*D*-valinate (21b). A solution of ester **20b** (14.0 mg, 0.0208 mmol) in *t*-BuOH (800 μL) was treated with LiOH•H_2O (8.7 mg, 0.21 mmol, 10 equiv) and H_2O (200 μL), then stirred at rt for 3 h. The resulting mixture was acidified to pH 7 by the addition of 1 N HCl, diluted with H_2O (2 mL), and extracted with EtOAc (3 × 5 mL). The combined organic layers were dried (Na_2SO_4) and concentrated *in vacuo*. The crude carboxylic acid was used directly without further purification.

A solution of the crude carboxylic acid in anhydrous CH_2Cl_2 (2 mL) was treated with EDC•HCl (40.0 mg, 0.209 mmol, 10.0 equiv) and stirred at rt under Ar for 24 h. The resulting mixture was treated with brine (4 mL) and extracted with CH_2Cl_2 (3 × 5 mL). The combined organic layers were dried (Na_2SO_4) and concentrated *in vacuo*. The crude azlactone was used directly in the next step without further purification.

A solution of the crude azlactone in anhydrous DMF (2 mL) was treated with DMAP (5.1 mg, 0.042 mmol, 2.0 equiv), D-Val-OMe•HCl (35.0 mg, 0.209 mmol, 10.0 equiv), and Et_3N (43.5 μL, 31.6 mg, 0.312 mmol, 15.0 equiv). The resulting mixture was stirred at 65 °C under Ar for 72 h, then diluted with H_2O (4 mL) and extracted with EtOAc (3 × 5 mL). The combined organic layers were washed with brine (2 × 5 mL), dried (Na_2SO_4) and concentrated *in vacuo*. Flash

chromatography (30 mL of SiO_2, 0–4% MeOH in CH_2Cl_2 gradient elution) afforded **21b** (5.0 mg, 0.0066 mmol, 32%) as a white film: $[\alpha]^{25}_{D}$ –4.6 (*c* 0.57, $CHCl_3$); ^1H NMR ($CDCl_3$, 500 MHz, data for major rotamer) δ 8.39 (br s, 1H), 7.63 (br s, 1H), 7.03 (d, *J* = 7.4 Hz, 1H), 5.46 (br s, 1H), 5.40 (br s, 1H), 4.50 (dd, *J* = 8.2, 5.4 Hz, 1H), 4.24–4.17 (m, 1H), 4.11–4.04 (m, 1H), 4.03 (dd, *J* = 15.3, 5.2 Hz, 1H), 3.71 (s, 3H), 2.25–2.12 (m, 4H), 2.09 (s, 3H), 1.82 (s, 3H), 1.76–1.53 (m, 3H), 1.45 (s, 9H), 1.26 (s, 3H), 1.10 (t, *J* = 7.4 Hz, 3H), 1.04 (t, *J* = 7.6 Hz, 3H), 1.02–0.95 (m, 12H), 0.93–0.80 (m, 6H), 0.68 (q, *J* = 7.9 Hz, 6H); ^{13}C NMR ($CDCl_3$, 125 MHz, data for major rotamer) δ 172.9, 170.8, 169.5, 166.1, 165.8, 157.2, 138.0, 136.7, 123.3, 119.6, 81.2, 77.2 (overlap with solvent), 62.1, 57.9, 52.0, 44.0, 32.1, 31.0, 28.5 (3C), 24.6, 22.9, 21.0, 19.3, 18.6, 18.3, 14.4, 13.6, 12.3, 8.9, 7.3 (3C), 6.9 (3C); IR (film) ν_{max} 3303, 2957, 2924, 2853, 1665, 1515, 1367, 1166, 1068, 1007 cm^{-1}; HRMS (ESI) *m/z* 754.4792 (MH^+, $C_{37}H_{67}N_5O_9SiH^+$ requires 754.4786).

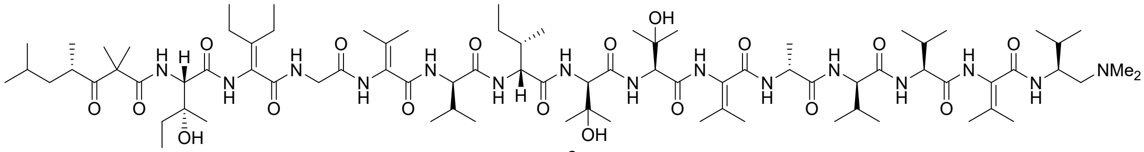

2a

EVV (2a). A solution of ester **21a** (11.6 mg, 0.0154 mmol) in *t*-BuOH (750 μL) and H_2O (250 μL) at 0 °C was treated with LiOH•H_2O (3.2 mg, 0.076 mmol, 5.0 equiv), then stirred at rt for 4 h. The resulting mixture was acidified to pH 4~5 by the addition of 1N HCl, diluted with H_2O (2 mL), and extracted with $CHCl_3$ (5 × 5 mL). The combined organic layers were dried (Na_2SO_4) and concentrated *in vacuo*. The crude carboxylic acid **4a** was used directly in the coupling with the amine derived from **5** without further purification.

A solution of nonapeptide **5** (16.0 mg, 0.0154 mmol) in anhydrous CH_2Cl_2 (1 mL) at 0 °C under Ar was treated with HCl (4.0 M in dioxane, 170 μL, 0.68 mmol, 44 equiv). The resulting

mixture was stirred at rt for 5 h, then concentrated *in vacuo*. The crude nonapeptide amine was purified using reverse-phase HPLC (COSMOSIL π-nap, 10 × 250 mm, 10–40% *n*-PrOH in H_2O with 1% AcOH gradient over 50 min, 2.5 mL/min flow rate, UV detection at 226 nm, t_R = 16.3 min) to afford the free amine derived from **5**.

A solution of crude carboxylic acid **4a** (ca. 0.0154 mmol) in anhydrous CH_2Cl_2 (1 mL) at 0 °C under Ar was treated with HOBt (ca. 20% H_2O content, 8.3 mg, 0.049 mmol, 3.2 equiv) and EDC•HCl (11.8 mg, 0.0616 mmol, 4.0 equiv). The resulting mixture was stirred at 0 °C under Ar for 20 min, then treated with a solution of the nonapeptide amine (ca. 0.0154 mmol, 1.0 equiv) in anhydrous CH_2Cl_2 (1 mL) and stirred at rt for 15 h. The reaction was quenched by the addition of sat aq $NaHCO_3$ (2.0 mL) and extracted with $CHCl_3$ (5 × 4 mL). The combined organic layers were dried (Na_2SO_4) and concentrated *in vacuo*. The residue was purified via flash chromatography (15 mL of SiO_2, 0–10% MeOH in $CHCl_3$ gradient elution) to afford tetradecapeptide **22a** (7.0 mg, 0.0042 mmol, 27%) as a white film. The purity of **22a** was deemed suitable for proceeding to the next step based on TLC analysis. Accordingly, the purification by HPLC that would have been required for rigorous spectral characterization was not pursued.

A solution of tetradecapeptide **22a** (7.0 mg, 0.0042 mmol) in anhydrous CH_2Cl_2 (1 mL) at 0 °C under Ar was treated with HCl (4.0 M in dioxane, 50 μL, 0.20 mmol, 47 equiv). The resulting mixture was stirred at rt for 4 h, then concentrated *in vacuo*. The crude amine was used directly in the coupling with **3** without further purification.

A solution of carboxylic acid **3**[2] (10.0 mg, 0.0499 mmol, 11.8 equiv) in anhydrous DMF (500 μL) at 0 °C under Ar was treated with COMU (21.5 mg, 0.0502 mmol, 11.9 equiv) and 2,4,6-collidine (10 μL, 9.2 mg, 0.076 mmol, 18 equiv). The resulting mixture was allowed to warm to rt

and was stirred under Ar for 30 min, then treated with a solution of the crude tetradecapeptide amine (ca. 0.0042 mmol) in anhydrous DMF (500 μL) at 0 °C. The resulting mixture was stirred at rt under Ar for 15 h, then cooled to 0 °C. The reaction was quenched by the addition of sat aq $NaHCO_3$ (1.5 mL) at 0 °C, diluted with EtOAc (3 mL), and washed with brine (10 × 4 mL). The combined organic layers were dried (Na_2SO_4) and concentrated *in vacuo*. The residue was purified via flash chromatography (15 mL of SiO_2, 0–7% MeOH in $CHCl_3$ with gradient elution) to afford semi-pure **2a** (5.7 mg). Further purification using reverse-phase HPLC (COSMOSIL π-nap, 10 × 250 mm, 80–85% *n*-PrOH in H_2O with 1% AcOH gradient over 70 min, 2.5 mL/min flow rate, UV detection at 226 nm, t_R = 11.8 min) afforded **2a** (1.2 mg, 0.00074 mmol, 17%) as a white solid: $[\alpha]^{25}_D$ +5.1 (*c* 0.53, MeOH); ^1H NMR (DMSO-d_6, 500 MHz) δ 10.05 (br s, 1H), 9.34 (br s, 1H), 9.10 (s, 1H), 8.81 (s, 1H), 8.53 (d, *J* = 7.5 Hz, 1H), 8.24 (d, *J* = 6.3 Hz, 1H), 8.03 (br s, 1H), 7.91 (br s, 1H), 7.84 (t, *J* = 9.6 Hz, 1H), 7.73–7.67 (m, 1H), 7.39 (d, *J* = 7.4 Hz, 1H), 7.23 (br s, 1H), 7.12 (d, *J* = 9.1 Hz, 1H), 6.69 (br s, 1H), 4.41–4.34 (m, 2H), 4.31 (d, *J* = 8.6 Hz, 1H), 4.23–4.17 (m, 2H), 4.15–4.09 (m, 2H), 4.06 (t, *J* = 7.4 Hz, 1H), 3.82–3.76 (m, 2H), 3.70–3.66 (m, 2H), 2.85–2.78 (m, 1H), 2.40–2.36 (m, 2H), 2.24–2.18 (m, 3H), 2.10 (s, 6H), 2.04 (s, 3H), 2.02–1.97 (m, 3H), 1.92 (s, 3H), 1.89 (s, 3H), 1.78–1.73 (m, 2H), 1.69 (s, 6H), 1.66 (s, 3H), 1.65 (s, 3H), 1.63 (s, 3H), 1.54–1.44 (m, 3H), 1.37 (s, 3H), 1.33–1.30 (m, 1H), 1.28 (s, 3H), 1.20 (s, 3H), 1.20–1.18 (m, 3H), 1.16 (s, 3H), 1.14 (s, 3H), 1.14–1.11 (m, 1H), 1.11 (s, 3H), 1.07 (s, 3H), 1.07–1.04 (m, 2H), 0.99 (t, *J* = 7.4 Hz, 3H), 0.95–0.91 (m, 6H), 0.90–0.76 (m, 33H); ^{13}C NMR (DMSO-d_6, 125 MHz) δ 213.5, 175.2, 174.7, 173.1, 172.6, 171.8, 171.3, 170.8, 170.3, 169.7, 169.1, 167.5, 165.7, 165.5, 165.1, 134.8, 132.1, 130.1, 129.1, 126.1, 124.7, 123.8, 119.1, 73.7, 72.0, 71.7, 67.9, 61.1, 60.9, 58.9, 58.6, 56.8, 56.7, 56.6, 51.6, 49.3, 45.9 (2C), 43.3, 38.5, 35.6, 31.7, 30.3, 29.6, 29.3, 29.2, 29.1, 29.0, 28.8, 28.4, 28.0, 27.0, 26.1, 25.6, 25.2, 25.0, 24.0, 23.9, 23.7, 22.9, 22.6, 22.3, 22.2,

21.8, 21.7, 21.3, 21.2, 20.8, 20.7, 19.9, 19.8, 18.8, 18.5, 17.9, 17.7, 15.1, 14.4, 13.5, 12.3, 12.0, 11.3, 8.6; IR (film) ν_{max} 3310, 2924, 2854, 2362, 1763, 1662, 1653, 1553, 1457, 1392, 1245, 1180, 1076 cm^{-1}; HRMS (ESI) *m/z* 1627.0812 (MH^+, $C_{82}H_{143}N_{15}O_{18}H^+$ requires 1627.0808).

HPLC Chromatogram of purified **2a** (COSMOSIL π-nap, 4.6 × 250 mm, 80–82% *n*-PrOH in H_2O with 1% AcOH gradient over 20 min, 0.6 mL/min flow rate, UV detection at 214 nm)

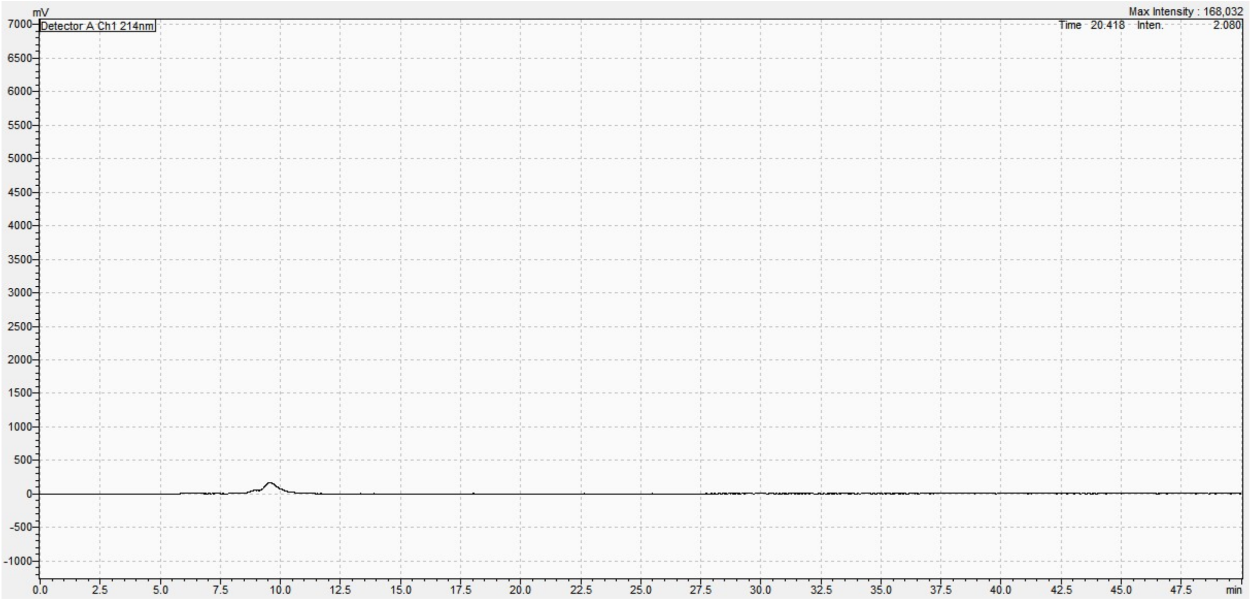

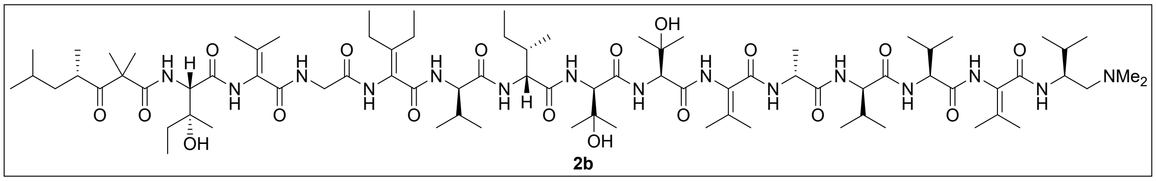

VEV (2b). A solution of ester **21b** (3.6 mg, 0.0048 mmol) in *t*-BuOH (750 μL) and H_2O (250 μL) at 0 °C was treated with LiOH•H_2O (1.0 mg, 0.024 mmol, 5.0 equiv), then stirred at rt for 4 h. The resulting mixture was acidified to pH 4~5 by the addition of 1N HCl, diluted with H_2O (2 mL), and extracted with $CHCl_3$ (5 × 5 mL). The combined organic layers were dried (Na_2SO_4) and concentrated *in vacuo*. The crude carboxylic acid **4b** was used directly in the coupling with the amine derived from **5** without further purification.

A solution of nonapeptide **5** (7.0 mg, 0.0067 mmol) in anhydrous CH_2Cl_2 (1 mL) at 0 °C under Ar was treated with HCl (4.0 M in dioxane, 70 μL, 0.28 mmol, 41 equiv). The resulting mixture was stirred at rt for 5 h, then concentrated *in vacuo*. The crude nonapeptide amine was purified using reverse-phase HPLC (COSMOSIL π-nap, 10 × 250 mm, 10–40% *n*-PrOH in H_2O with 1% AcOH gradient over 50 min, 2.5 mL/min flow rate, UV detection at 226 nm, t_R = 16.3 min) to afford the free amine derived from **5**.

A solution of crude carboxylic acid **4b** (ca. 0.0048 mmol) in anhydrous CH_2Cl_2 (1 mL) at 0 °C under Ar was treated with HOBt (ca. 20% H_2O content, 2.6 mg, 0.015 mmol, 3.2 equiv) and EDC•HCl (3.7 mg, 0.019 mmol, 4.0 equiv). The resulting mixture was stirred at 0 °C under Ar for 20 min, then treated with a solution of the nonapeptide amine (ca. 0.0067 mmol, 1.4 equiv) in anhydrous CH_2Cl_2 (500 μL) and stirred at rt for 15 h. The reaction was quenched by the addition of sat aq $NaHCO_3$ (2 mL) and extracted with $CHCl_3$ (5 × 4 mL). The combined organic layers were dried (Na_2SO_4) and concentrated *in vacuo*. The residue was purified via flash chromatography (15 mL of SiO_2, 0–15% MeOH in $CHCl_3$ gradient elution) to afford tetradecapeptide **22b** (2.4 mg, 0.0014 mmol, 30%) as a white film. The purity of **22b** was deemed suitable for proceeding to the next step based on TLC analysis. Accordingly, the purification by HPLC that would have been required for rigorous spectral characterization was not pursued.

A solution of tetradecapeptide **22b** (2.4 mg, 0.0014 mmol) in anhydrous CH_2Cl_2 (1 mL) at 0 °C under Ar was treated with HCl (4.0 M in dioxane, 15.5 μL, 0.062 mmol, 43 equiv). The resulting mixture was stirred at rt for 4 h, then concentrated *in vacuo*. The crude amine was used directly in the coupling with **3** without further purification.

A solution of carboxylic acid **3**[2] (3.1 mg, 0.015 mmol, 11 equiv) in anhydrous DMF (100 μL) at 0 °C under Ar was treated with COMU (6.2 mg, 0.014 mmol, 10 equiv) and 2,4,6-collidine (3.8 μL, 3.5 mg, 0.029 mmol, 20 equiv). The resulting mixture was allowed to warm to rt and was stirred under Ar for 30 min, then treated with a solution of the crude tetradecapeptide amine (ca. 0.0014 mmol) in anhydrous DMF (200 μL) at 0 °C. The resulting mixture was stirred under Ar at rt for 15 h, then cooled to 0 °C. The reaction was quenched by the addition of sat aq $NaHCO_3$ (1 mL) at 0 °C, diluted with EtOAc (2 mL), and washed with brine (10 × 3 mL). The combined organic layers were dried (Na_2SO_4) and concentrated *in vacuo*. The residue was purified via flash chromatography (15 mL of SiO_2, 0–7% MeOH in $CHCl_3$ with gradient elution) to afford semi-pure **2a** (1.3 mg). Further purification using reverse-phase HPLC (COSMOSIL π-nap, 10 × 250 mm, 80–85% *n*-PrOH in H_2O with 1% AcOH gradient over 70 min, 2.5 mL/min flow rate, UV detection at 226 nm, t_R = 11.1 min) afforded **2b** (0.6 mg, 0.0004 mmol, 25%) as a white solid: $[\alpha]^{25}_D$ +6.7 (*c* 0.24, MeOH); ^{1}H NMR (DMSO-d_6, 500 MHz) δ 10.00 (br s, 1H), 9.31 (br s, 1H), 9.10 (s, 1H), 8.81 (s, 1H), 8.51 (br s, 1H), 8.21 (br s, 1H), 7.98 (br s, 1H), 7.92 (br s, 1H), 7.83 (d, *J* = 8.3 Hz, 1H), 7.73–7.66 (m, 1H), 7.37 (br s, 1H), 7.22 (br s, 1H), 7.11 (d, *J* = 8.9 Hz, 1H), 6.67 (br s, 1H), 4.40–4.35 (m, 2H), 4.28 (d, *J* = 8.5 Hz, 1H), 4.22–4.16 (m, 2H), 4.14–4.10 (m, 2H), 4.06 (t, *J* = 7.6 Hz, 1H), 3.80–3.75 (m, 2H), 3.70-3.65 (m, 2H), 2.84–2.78 (m, 1H), 2.37–2.35 (m, 2H), 2.23–2.18 (m, 3H), 2.09 (s, 6H), 2.03 (s, 3H), 2.00–1.95 (m, 3H), 1.91 (s, 6H), 1.77–1.72 (m, 2H), 1.67 (s, 3H), 1.65 (s, 9H), 1.62 (s, 3H), 1.47–1.43 (m, 3H), 1.35 (s, 3H), 1.34–1.32 (m, 1H), 1.29 (s, 3H), 1.27 (s, 3H), 1.25 (s, 3H), 1.19 (s , 3H), 1.14 (s, 3H), 1.13 (s, 3H), 1.11 (s, 3H), 1.09–1.07 (m, 1H), 1.06 (s, 3H), 1.05–1.03 (m, 2H), 1.02 (s, 3H), 0.94–0.90 (m, 6H), 0.87–0.77 (m, 33H); ^{13}C NMR (DMSO-d_6, 125 MHz) δ 213.5, 174.8, 173.0, 172.6, 171.8, 171.3, 170.8, 169.7, 169.5, 166.3, 165.8, 165.5, 165.0, 158.3, 158.0, 132.1, 130.1, 129.1, 128.2, 126.1, 124.5, 119.1,

116.7, 73.6, 72.0, 71.7, 67.9, 67.5, 61.1 58.9, 58.5, 56.6, 56.1, 55.4, 51.6, 49.3, 45.9 (2C), 43.3, 38.5, 35.6, 31.7, 30.3, 29.6, 29.5, 29.3, 29.2, 29.0, 28.8, 28.4, 28.1, 27.1, 27.0, 26.1, 26.0, 25.6, 25.2, 23.9, 23.7, 22.9, 22.6, 22.3, 22.2, 21.8, 21.2, 20.8, 20.7, 19.9, 19.8, 18.9, 18.7, 18.5, 18.0, 17.9, 17.7, 15.0, 14.4, 13.5, 12.3, 12.0, 11.3, 8.7; IR (film) ν_{max} 3312, 3312, 2925, 2891, 2314, 1656, 1648, 1595, 1534, 1411, 1366, 1205, 1137, cm^{-1}; HRMS (ESI) *m/z* 1627.0810 (MH^+, $C_{82}H_{143}N_{15}O_{18}H^+$ requires 1627.0808).

HPLC Chromatogram of purified **2b** (COSMOSIL π-nap, 4.6 × 250 mm, 80–82% *n*-PrOH in H_2O with 1% AcOH gradient over 20 min, 0.6 mL/min flow rate, UV detection at 214 nm)

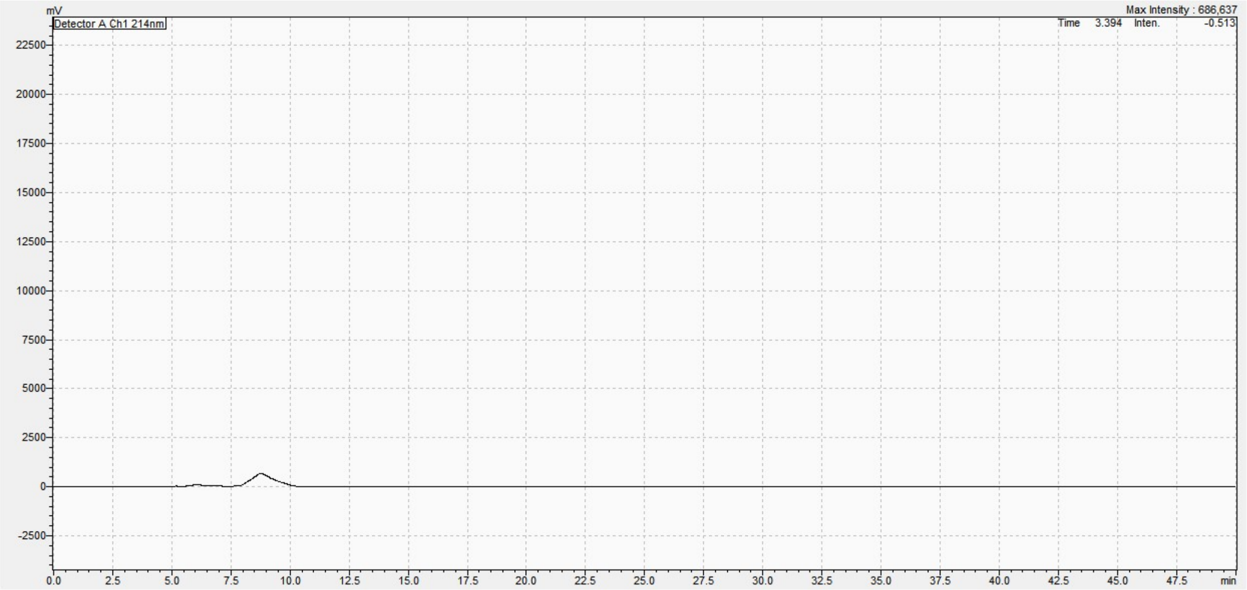

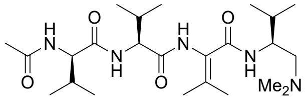

23

2-((*S*)-2-((*R*)-2-Acetamido-3-methylbutanamido)-3-methylbutanamido)-*N*-((*S*)-1-(dimethylamino)-3-methylbutan-2-yl)-3-methylbut-2-enamide (23). A solution of tetrapeptide **8** (77.5 mg, 0.147 mmol) in anhydrous CH_2Cl_2 (1 mL) at 0 °C under Ar was treated with HCl (4.0 M in dioxane, 700 µL, 2.8 mmol, 19 equiv). The resulting

mixture was stirred at rt for 2 h and concentrated *in vacuo*. The crude amine was used directly in the following coupling without further purification.

A solution of the crude amine derived from **8** (ca. 0.147 mmol) in anhydrous CH_2Cl_2 (1 mL) under Ar was treated with Ac_2O (70 µL, 76 mg, 0.74 mmol, 5.0 equiv) and Et_3N (100 µL, 72.6 mg, 0.717 mmol, 4.9 equiv). The resulting mixture was stirred at rt for 2 h, then treated with sat aq $NaHCO_3$ (2 mL) and extracted with $CHCl_3$ (3 × 5 mL). The combined organic layers were washed with brine (5 mL), dried (Na_2SO_4), and concentrated *in vacuo.* Flash chromatography (30 mL of SiO_2, 0.5–5% MeOH in $CHCl_3$ with 1% Et_3N gradient elution) afforded **23** (14.0 mg, 0.0299 mmol, 20%) as a pale brown solid: $[\alpha]^{25}_D$ +32 (*c* 0.31, $CHCl_3$); ^{1}H NMR ($CDCl_3$, 500 MHz) δ 10.34 (br s, 1H), 8.90 (br s, 1H), 8.12 (br s, 1H), 6.46 (br s, 1H), 4.51 (dd, *J* = 9.2, 4.0 Hz, 1H), 4.25–4.18 (m, 1H), 3.63–3.56 (m, 1H), 3.52 (t, *J* = 12.2 Hz, 1H), 2.94 (d, *J* = 4.9 Hz, 3H), 2.88 (d, *J* = 4.9 Hz, 3H), 3.00–2.96 (m, 1H), 2.48–2.41 (m, 1H), 2.38–2.31 (m, 1H), 2.13 (s, 3H), 2.03 (s, 3H), 1.90 (s, 3H), 1.67–1.59 (m, 1H), 1.00 (d, *J* = 6.6 Hz, 3H), 0.93 (d, *J* = 6.6 Hz, 6H), 0.89 (d, *J* = 6.8 Hz, 6H), 0.85 (d, *J* = 6.8 Hz, 3H); ^{13}C NMR ($CDCl_3$, 125 MHz) δ 175.8, 174.3, 171.5, 165.6, 143.4, 122.3, 63.7, 60.5, 58.0, 49.4, 46.3, 42.5, 31.5, 29.7, 29.5, 23.3, 22.6, 21.1, 20.4, 19.9, 19.5, 19.2, 18.9, 18.6; IR (film) ν_{max} 3270, 2963, 2926, 2846, 1654, 1534, 1466, 1373, 1307, 1262, 1222, 1172, 1111 cm^{-1}; HRMS (ESI) *m/z* 468.3540 (MH^+, $C_{24}H_{45}N_5O_4H^+$ requires 468.3544).

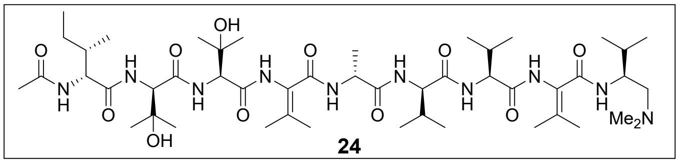

(2*R*,3*S*)-2-Acetamido-*N*-((4*S*,10*S*,13*R*,16*R*,22*S*,25*R*)-26-hydroxy-22-(2-hydroxypropan-2-yl)-4,10,13-triisopropyl-2,16,26-trimethyl-6,9,12,15,18,21,24-heptaoxo-7,19-di(propan-2-ylidene)-2,5,8,11,14,17,20,23-octaazaheptacosan-25-yl)-3-methylpentanamide (24). A solution of

nonapeptide **5** (4.1 mg, 0.0040 mmol) in anhydrous CH_2Cl_2 (500 μL) at 0 °C under Ar was treated with HCl (4.0 M in dioxane, 40 μL, 0.16 mmol, 40 equiv). The resulting mixture was stirred at rt for 5 h, concentrated *in vacuo*, and purified using reverse-phase HPLC (COSMOSIL π-nap, 10 × 250 mm, 10–40% *n*-PrOH in H_2O with 1% AcOH gradient over 50 min, 2.5 mL/min flow rate, UV detection at 226 nm, t_R = 16.3 min) to afford the free amine derived from **5**.

A solution of nonapeptide amine (ca. 0.0040 mmol) in anhydrous DMF (500 μL) at rt under Ar was treated with Ac_2O (3.6 μL, 3.9 mg, 0.038 mmol, 9.7 equiv) and Et_3N (5.3 μL, 3.8 mg, 0.0381 mmol, 9.6 equiv). The resulting mixture was stirred at rt for 2 h, at which point the reaction was complete according to MS analysis. The mixture was treated with sat aq $NaHCO_3$ (1 mL) and extracted with EtOAc (2 mL). The organic layer was washed with brine (10 × 4 mL), dried (Na_2SO_4), and concentrated *in vacuo.* Acylated nonapeptide **24** was obtained as a white solid (3.1 mg, 0.0032 mmol, 80%)): $[\alpha]^{25}_D$ –19.7 (*c* 3.3, MeOH); ^{1}H NMR ($CDCl_3$, 500 MHz, data for major rotamer) δ 10.10 (br s, 1H), 8.63 (br s, 1H), 8.28 (d, *J* = 6.6 Hz, 1H), 8.14 (d, *J* = 9.6 Hz, 1H), 8.04 (br s, 1H), 7.92 (br s, 1H), 7.75–7.70 (m, 1H), 7.58–7.52 (m, 1H), 7.36 (s, 1H), 5.49 (br s, 1H), 5.36 (br s, 1H), 4.83 (d, *J* = 10.0 Hz, 1H), 4.38–4.35 (m, 1H), 4.33–4.28 (m, 1H), 4.25–4.20 (m, 2H), 4.12–4.10 (m, 1H), 3.92 (d, *J* = 5.6 Hz, 1H), 3.86–3.84 (m, 1H), 3.66 (s, 3H), 3.48 (dd, *J* = 10.6, 4.5 Hz, 1H), 3.11 (d, *J* = 4.9 Hz, 3H), 3.05 (d, *J* = 4.9 Hz, 3H), 2.48–2.43 (m, 1H), 2.39 (s, 3H), 2.36 (s, 3H), 2.33–2.29 (m, 2H), 2.25–2.22 (m, 1H), 2.00 (s, 3H), 1.82 (s, 3H), 1.62 (s, 3H), 1.60 (s, 3H), 1.59 (s, 3H), 1.58 (s, 3H), 1.47 (s, 3H), 1.44–1.41 (m, 3H), 1.04–0.99 (m, 2H), 0.96 (t, *J* = 7.0 Hz, 3H), 0.92–0.85 (m, 18H); ^{13}C NMR (DMSO-d_6, 125 MHz, data for major rotamer) δ 175.0, 172.8, 172.2, 171.7, 170.3, 169.8, 166.5, 165.2, 138.3, 129.2, 125.5, 124.4, 79.7, 77.8, 71.8, 70.2, 60.4, 59.0, 58.6, 56.5, 50.0, 49.1, 46.1 (2C), 45.3, 42.3, 36.9, 34.1, 31.8, 30.8, 29.2, 28.2, 26.3, 25.9, 25.0, 23.0, 22.6, 20.7, 19.8, 19.7, 19.0, 18.74, 18.65, 17.7, 15.0, 14.4, 12.1,

9.1; IR (film) ν_{max} 3421, 3303, 2922, 2815, 2348, 2090, 1653, 1647, 1559, 1540, 1507, 1457, 1374, 1122, cm^{-1}; HRMS (ESI) *m/z* 979.6545 (MH^+, $C_{48}H_{86}N_{10}O_{11}H^+$ requires 979.6550).

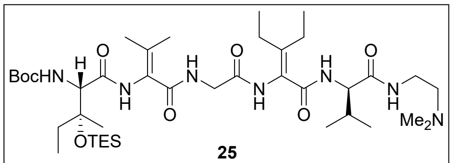

25

***tert*-Butyl ((7*R*,19*S*,20*R*)-20,22,22-Triethyl-7-isopropyl-2,20-dimethyl-6,9,12,15,18-pentaoxo-10-(pentan-3-ylidene)-16-(propan-2-ylidene)-21-oxa-2,5,8,11,14,17-hexaaza-22-silatetracosan-19-yl)carbamate (25).** A solution of pentapeptide **21b** (5.5 mg, 0.0073 mmol) in *t*-BuOH (750 μL) and H_2O (250 μL) was treated with LiOH•H_2O (1.8 mg, 0.043 mmol, 5.9 equiv) and stirred at rt for 5 h. The resulting mixture was acidified to pH 4~5 by the addition of 1 N HCl, diluted with H_2O (2 mL), and extracted with CH_2Cl_2 (3 × 5 mL). The combined organic layers were dried (Na_2SO_4) and concentrated *in vacuo*. The crude carboxylic acid **4b** was used directly without further purification.

A solution of crude carboxylic acid **4b** (ca. 0.0073 mmol. 1.0 equiv) in anhydrous CH_2Cl_2 (500 μL) at 0 °C under Ar was treated with HOBt (ca. 20% H_2O content, 2.7 mg, 0.016 mmol, 2.2 equiv) and EDC•HCl (3.8 mg, 0.020 mmol, 2.7 equiv). The resulting mixture was stirred at 0 °C under Ar for 20 min, then treated with a solution of *N*,*N*-dimethylethylenediamine (2.1 μL, 1.7 mg, 0.019 mmol, 2.6 equiv) in anhydrous CH_2Cl_2 (500 μL) and stirred at rt for 15 h. The reaction was quenched by the addition of sat aq $NaHCO_3$ (1 mL) and extracted with $CHCl_3$ (5 × 3 mL). The combined organic layers were dried (Na_2SO_4) and concentrated *in vacuo*. Flash chromatography (15 mL of SiO_2, 0–15% MeOH in $CHCl_3$ gradient elution) afforded **25** (4.0 mg, 0.0049 mmol, 68%) as a colorless oil: $[\alpha]^{25}_D$ –10.3 (*c* 0.35, $CHCl_3$); ^1H NMR ($CDCl_3$, 500 MHz, data for major rotamer) δ 8.83 (1 br s, 1H), 8.21 (br s, 1H), 7.99 (br s, 1H), 7.67 (d, *J* = 6.4 Hz,

1H), 7.42 (br s, 1H), 5.38 (br s, 1H), 4.26–4.20 (m, 1H), 4.10 (d, J = 4.7 Hz, 1H), 4.04 (d, J = 5.4 Hz, 1H), 3.76–3.69 (m, 1H), 3.12–3.00 (m, 2H), 2.79 (s, 3H), 2.67 (s, 3H), 2.41–2.33 (m, 2H), 2.32–2.24 (m, 2H), 2.11–2.04 (m, 2H), 1.99 (s, 3H), 1.72 (s, 3H), 1.64–1.50 (m, 3H), 1.37 (s, 9H), 1.29 (s, 3H), 1.00 (t, J = 7.4 Hz, 3H), 0.96 (t, J = 7.4 Hz, 3H), 0.93–0.87 (m, 15H), 0.85 (t, J = 7.2 Hz, 3H); 0.65–0.55 (m, 6H); ^{13}C NMR ($CDCl_3$, 125 MHz, data for major rotamer) δ 172.9, 170.5, 169.8, 167.6, 166.7, 156.8, 151.0, 150.1, 124.6, 122.7, 80.3, 78.1, 62.2, 61.9, 60.6, 59.7, 57.5, 44.6, 43.9, 34.4, 32.6, 28.3 (3C), 24.9, 24.4, 24.3, 21.5, 20.8, 19.4, 18.5, 13.5, 12.2, 8.8, 7.1 (3C), 6.7 (3C); IR (film) ν_{max} 3352, 3296 2962, 2921, 2887, 1660, 1543, 1362, 1167, 1023, 1006 cm^{-1}; HRMS (ESI) *m/z* 810.5524 (MH^+, $C_{40}H_{75}N_7O_8SiH^+$ requires 810.5519).

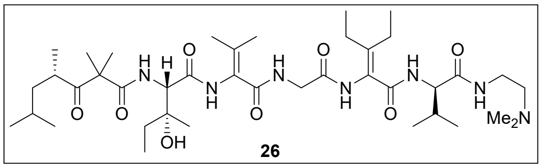

(*S*)-*N*-((7*R*,19*S*,20*R*)-20-Hydroxy-7-isopropyl-2,20-dimethyl-6,9,12,15,18-pentaoxo-10-(pentan-3-ylidene)-16-(propan-2-ylidene)-2,5,8,11,14,17-hexaazadocosan-19-yl)-2,2,4,6-tetramethyl-3-oxoheptanamide (26). A solution of pentapeptide **25** (4.0 mg, 0.0049 mmol) in anhydrous CH_2Cl_2 (700 μL) at 0 °C under Ar was treated with HCl (4.0 M in dioxane, 53 μL, 0.21 mmol, 43 equiv). The resulting mixture was stirred at rt for 5 h, then concentrated *in vacuo*. The crude pentapeptide amine was used directly without further purification.

A solution of carboxylic acid **3**[2] (8.2 mg, 0.041 mmol, 8.3 equiv) in anhydrous DMF (500 μL) at 0 °C under Ar was treated with COMU (19.0 mg, 0.0444 mmol, 9.0 equiv) and 2,4,6-collidine (12 μL, 11 mg, 0.091 mmol, 18 equiv). The resulting mixture was allowed to warm to rt and was stirred under Ar for 30 min, then treated with a solution of the crude pentapeptide amine (ca.

0.0049 mmol) in anhydrous DMF (500 μL) at 0 °C. The resulting mixture was stirred under Ar at rt for 15 h, then cooled to 0 °C. The reaction was quenched by the addition of sat aq $NaHCO_3$ (1 mL) at 0 °C, diluted with EtOAc (2 mL), and washed with brine (10 × 3 mL). The organic layer was dried (Na_2SO_4) and concentrated *in vacuo*. The residue was purified via flash chromatography (15 mL of SiO_2, 0–15% MeOH in $CHCl_3$ with gradient elution) to afford **26** (2.9 mg, 0.0037 mmol, 75%) as a colorless oil that was a mixture of rotamers: $[\alpha]^{25}_{D}$ –18.8 (*c* 0.16, $CHCl_3$); ^1H NMR ($CDCl_3$, 500 MHz, data for major rotamer) δ 9.47 (s, 1H), 9.36 (s, 1H), 9.11 (br s, 1H), 8.58 (s, 1H), 8.17 (br s, 1H), 7.97 (br s, 1H), 4.75 (d, *J* = 8.9 Hz, 1H), 4.35–4.25 (m, 2H), 3.88–3.79 (m, 1H), 3.77–3.73 (m, 1H), 3.69 (d, *J* = 14.6 Hz, 1H), 3.25 (d, *J* = 12.6 Hz, 1H), 3.02 (s, 6H), 2.92–2.87 (m, 1H), 2.59–2.52 (m, 2H), 2.48–2.42 (m, 2H), 2.35 (t, *J* = 7.3 Hz, 2H), 2.15 (d, *J* = 7.6 Hz, 1H), 1.99 (s, 3H), 1.89–1.82 (m, 2H), 1.76 (s, 3H), 1.71 (s, 3H), 1.67–1.61 (m, 3H), 1.56 (s, 3H), 1.44 (s, 3H), 1.05 (d, *J* = 6.2 Hz, 3H), 0.97 (t, *J* = 6.2 Hz, 3H), 0.92–0.84 (m, 18H); ^{13}C NMR ($CDCl_3$, 125 MHz, data for major rotamer) δ 217.4, 173.8, 173.3, 172.9, 172.1, 170.6, 168.4, 149.6, 141.3, 129.5, 123.3, 76.5, 64.0, 59.6, 58.1, 57.2, 45.7, 45.3, 43.2, 42.8, 38.4, 33.8, 33.3, 32.0, 29.4, 28.4, 25.5, 24.9, 24.2, 23.4, 22.7, 21.9, 21.3, 20.1, 19.1, 17.6, 14.2, 13.4, 12.4, 8.2; IR (film) ν_{max} 3792, 3287, 2957, 2924, 2852, 1710, 1658, 1529, 1462, 1376, 1261, 1163, 1024 cm^{-1}; HRMS (ESI) *m/z* 778.5442 (MH^+, $C_{40}H_{71}N_7O_8H^+$ requires 778.5437).

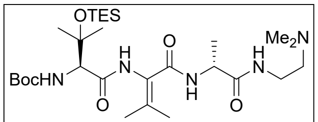

27

***tert*-Butyl ((7*R*,13*S*)-16,16-Diethyl-2,7,14,14-tetramethyl-6,9,12-trioxo-10-(propan-2-ylidene)-15-oxa-2,5,8,11-tetraaza-16-silaoctadecan-13-yl)carbamate (27).** A solution of tripeptide **13** (7.7 mg, 0.015 mmol) in *t*-BuOH (750 μL) and H_2O (250 μL) was treated with $LiOH•H_2O$ (3.1 mg, 0.074 mmol, 5.1 equiv) and stirred at rt for 5 h. The resulting

mixture was acidified to pH 4~5 by the addition of 1 N HCl, diluted with H_2O (2 mL), and extracted with CH_2Cl_2 (3 × 5 mL). The combined organic layers were dried (Na_2SO_4) and concentrated *in vacuo*. The crude carboxylic acid **7** was used directly without further purification.

A solution of crude carboxylic acid **7** (ca. 0.015 mmol, 1.0 equiv) in anhydrous CH_2Cl_2 (500 μL) at 0 °C under Ar was treated with HOBt (ca. 20% H_2O content, 5.9 mg, 0.035 mmol, 2.4 equiv) and EDC•HCl (8.4 mg, 0.044 mmol, 3.0 equiv). The resulting mixture was stirred at 0 °C under Ar for 20 min, then treated with a solution of DMEDA (5.0 μL, 4.0 mg, 0.046 mmol, 3.1 equiv) in anhydrous CH_2Cl_2 (500 μL) and stirred at rt for 15 h. The reaction was quenched by the addition of sat aq $NaHCO_3$ (1.5 mL) and extracted with CH_2Cl_2 (3 × 3 mL). The combined organic layers were dried (Na_2SO_4) and concentrated *in vacuo*. Flash chromatography (15 mL of SiO_2, 0–10% MeOH in $CHCl_3$ gradient elution) afforded **27** (3.9 mg, 0.0067 mmol, 46%) as a colorless oil: $[\alpha]^{25}_{D}$ +31 (*c* 0.87, $CHCl_3$); ^1H NMR ($CDCl_3$, 500 MHz, mixture of rotamers) δ 8.04 and 7.90 (2 br s, 1H), 7.60 (br s, 1H), 7.30 and 7.10 (2 br s, 1H), 5.41 and 5.37 (2 br s, 1H), 4.53–4.47 and 4.47–4.40 (2m, 1H), 4.05 and 3.87 (2d, *J* = 6.2 and 4.7 Hz, 1H), 3.60–3.45 (m, 1H), 3.42–3.32 and 3.30–3.18 (2m, 1H), 2.80–2.61 (m, 2H), 2.44 (s, 3H), 2.41 (s, 3H), 2.00 and 1.92 (2s, 3H), 1.72 (s, 3H), 1.41 (d, *J* = 7.0 Hz, 3H), 1.37 and 1.36 (2s, 9H), 1.33 and 1.31 (2s, 3H), 1.23 and 1.18 (2s, 3H), 0.91 (t, *J* = 7.9 Hz, 9H), 0.60 (q, *J* = 7.9 Hz, 6H); ^{13}C NMR ($CDCl_3$, 125 MHz, mixture of rotamers) δ 173.1 and 172.7, 169.7 and 169.6, 165.3 and 165.2, 156.8 and 156.5, 134.3, 124.9 and 123.7, 80.7, 80.1, 76.2 and 74.5, 65.1 and 63.2, 57.9 and 57.7, 49.6 and 49.0, 44.6 (2C), 36.1, 28.34 and 28.28 (3C), 27.9 and 27.4, 25.7, 20.7 and 20.5, 17.3 and 17.2, 7.0 (3C), 6.5 (3C); IR (film) ν_{max} 3439, 3318, 2954, 2937, 2876, 2243, 1700, 1504, 1367, 1167 cm^{-1}; HRMS (ESI) *m/z* 586.3991 (MH^+, $C_{28}H_{55}N_5O_6SiH^+$ requires 586.3994).

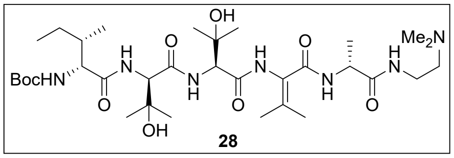

***tert*-Butyl ((7*R*,13*S*,16*R*,19*R*,20*S*)-13,16-Bis(2-hydroxypropan-2-yl)-2,7,20-trimethyl-6,9,12,15,18-pentaoxo-10-(propan-2-ylidene)-2,5,8,11,14,17-hexaazadocosan-19-yl)carbamate (28).** A solution of ethyl (*R*)-2-((2*R*,3*S*)-2-((*tert*-butoxycarbonyl)amino)-3-methylpentanamido)-3-hydroxy-3-methylbutanoate[2] (5.1 mg, 0.014 mmol) in *t*-BuOH (750 μL) and H_2O (250 μL) at 0 °C was treated with LiOH•H_2O (2.9 mg, 0.069 mmol, 5.1 equiv), then stirred at rt for 4.5 h. The resulting mixture was acidified to pH 4~5 by the addition of 1 N HCl, diluted with H_2O (2 mL), and extracted with CH_2Cl_2 (3 × 5 mL). The combined organic layers were dried (Na_2SO_4) and concentrated *in vacuo*. The crude carboxylic acid **6** was used directly without further purification.

A solution of **27** (6.0 mg, 0.010 mmol) in anhydrous CH_2Cl_2 (750 μL) at 0 °C under Ar was treated with HCl (4.0 M in dioxane, 80 μL, 0.32 mmol, 31 equiv). The resulting mixture was stirred at rt for 4 h, then concentrated *in vacuo*. The crude amine was used directly in the coupling with **6** without further purification.

A solution of crude carboxylic acid **6** (ca. 0.014 mmol. 1.3 equiv) in anhydrous CH_2Cl_2 (500 μL) at 0 °C under Ar was treated with HOBt (ca. 20% H_2O content, 3.7 mg, 0.022 mmol, 2.1 equiv) and EDC•HCl (5.2 mg, 0.027 mmol, 2.6 equiv). The resulting mixture was stirred at 0 °C under Ar for 20 min, then treated with a solution of the crude tripeptide (ca. 0.010 mmol) in anhydrous CH_2Cl_2 (500 μL) and stirred at rt for 15 h. The reaction was quenched by the addition of sat aq $NaHCO_3$ (2.0 mL) and extracted with $CHCl_3$ (5 × 5 mL). The combined organic layers were dried (Na_2SO_4) and concentrated *in vacuo*. Flash chromatography (15 mL of SiO_2, 0–5% MeOH in $CHCl_3$ with 1% Et_3N gradient elution) afforded **28** (5.6 mg, 0.0080 mmol, 78%) as a

white film: $[\alpha]^{25}_{D}$ –9.5 (*c* 0.21, $CHCl_3$); ^{1}H NMR ($CDCl_3$, 500 MHz, data for major rotamer) δ 8.67 (br s, 1H), 7.96 (br s, 1H), 7.64 (br s, 1H), 7.38 (br s, 1H), 7.11 (br s, 1H), 5.03 (br s, 1H), 4.49 (d, *J* = 8.2 Hz, 1H), 4.44–4.40 (m, 1H), 4.35–4.30 (m, 1H), 4.28–4.23 (m, 1H), 4.16–4.11 (m, 1H), 4.04–4.00 (m, 1H), 3.47–3.39 (m, 2H), 3.26–3.21 (m, 1H), 2.76 (d, *J* = 7.0 Hz, 3H), 2.53 (br s, 3H), 2.05–2.00 (m, 2H), 1.91 (s, 3H), 1.69 (s, 3H), 1.36 (s, 9H), 1.35–1.33 (m, 2H), 1.27 (s, 3H), 1.22 (d, *J* = 6.9 Hz, 3H), 1.18 (br s, 6H), 1.14 (t, *J* = 7.2 Hz, 3H), 0.88–0.83 (m, 3H), 0.81–0.76 (m, 3H); ^{13}C NMR ($CDCl_3$, 125 MHz, data for major rotamer) δ 172.3, 172.2, 171.3, 170.0, 155.2, 152.0, 123.0, 122.2, 79.6, 71.6, 71.2, 70.2, 60.6, 59.4, 57.9, 57.0, 44.8 (2C), 43.7, 36.3, 28.7, 27.2 (3C), 26.0, 25.5, 20.5, 20.0, 19.6, 19.4, 13.2, 10.7, 9.0; IR (film) ν_{max} 3305, 2971, 2927, 2875, 2855, 1657, 1525, 1462, 1366, 1240, 1166, 1042 cm^{-1}; HRMS (ESI) *m/z* 700.4606 (MH^+, $C_{33}H_{61}N_7O_9H^+$ requires 700.4604).

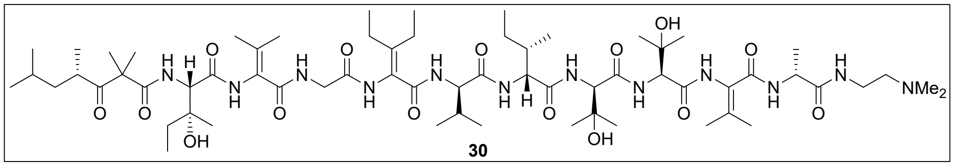

30

(*S*)-*N*-((7*R*,13*S*,16*R*,19*R*,22*R*,34*S*,35*R*)-19-((*S*)-*sec*-Butyl)-35-hydroxy-13,16-bis(2-hydroxypropan-2-yl)-22-isopropyl-2,7,35-trimethyl-6,9,12,15,18,21,24,27,30,33-decaoxo-25-(pentan-3-ylidene)-10,31-di(propan-2-ylidene)-2,5,8,11,14,17,20,23,26,29,32-undecaazaheptatriacontan-34-yl)-2,2,4,6-tetramethyl-3-oxoheptanamide (30). A solution of pentapeptide **21b** (5.5 mg, 0.0073 mmol) in *t*-BuOH (750 μL) and H_2O (250 μL) was treated with LiOH•H_2O (1.8 mg, 0.043 mmol, 5.9 equiv) and stirred at rt for 5 h. The resulting mixture was acidified to pH 4~5 by the addition of 1 N HCl, diluted with H_2O (2 mL), and extracted with CH_2Cl_2 (3 × 5 mL). The combined organic layers were dried (Na_2SO_4) and concentrated *in vacuo*. The crude carboxylic acid **4b** was used directly without further purification.

A solution of **28** (5.6 mg, 0.0080 mmol) in anhydrous CH_2Cl_2 (750 μL) at 0 °C under Ar was treated with HCl (4.0 M in dioxane, 64 μL, 0.26 mmol, 32 equiv). The resulting mixture was stirred at rt for 4 h, then concentrated *in vacuo*. The crude amine was used directly in the coupling with **4b** without further purification.

A solution of crude carboxylic acid **4b** (ca. 0.0073 mmol) in anhydrous CH_2Cl_2 (500 μL) at 0 °C under Ar was treated with HOBt (ca. 20% H_2O content, 3.2 mg, 0.019 mmol, 2.6 equiv) and EDC•HCl (4.6 mg, 0.024 mmol, 3.3 equiv). The resulting mixture was stirred at 0 °C under Ar for 20 min, then treated with a solution of the amine derived from **28** (ca. 0.0080 mmol, 1.1 equiv) in anhydrous CH_2Cl_2 (500 μL) and stirred at rt for 15 h. The reaction was quenched by the addition of sat aq $NaHCO_3$ (2 mL) and extracted with $CHCl_3$ (5 × 4 mL). The combined organic layers were dried (Na_2SO_4) and concentrated *in vacuo*. The residue was purified via flash chromatography (15 mL of SiO_2, 0–15% MeOH in $CHCl_3$ gradient elution) to afford **29** (4.2 mg, 0.0032 mmol, 44%) as a clear oil. The purity of **29** was deemed suitable for proceeding to the next step based on TLC analysis. Accordingly, the purification by HPLC that would have been required for rigorous spectral characterization was not pursued.

A solution of **29** (4.2 mg, 0.0032 mmol) in anhydrous CH_2Cl_2 (1 mL) at 0 °C under Ar was treated with HCl (4.0 M in dioxane, 34 μL, 0.14 mmol, 43 equiv). The resulting mixture was stirred at rt for 4 h, then concentrated *in vacuo*. The crude amine was used directly in the coupling with **3** without further purification.

A solution of carboxylic acid **3**[2] (7.6 mg, 0.038 mmol, 12 equiv) in anhydrous DMF (500 μL) at 0 °C under Ar was treated with COMU (15.1 mg, 0.0353 mmol, 11 equiv) and 2,4,6-collidine (9.0 μL, 8.3 mg, 0.068 mmol, 21 equiv). The resulting mixture was allowed to warm to rt and was

stirred under Ar for 30 min, then treated with a solution of the crude amine derived from **29** (ca. 0.0032 mmol) in anhydrous DMF (500 μL) at 0 °C. The resulting mixture was stirred at rt under Ar for 15 h, then cooled to 0 °C. The reaction was quenched by the addition of sat aq $NaHCO_3$ (1 mL) at 0 °C, diluted with EtOAc (2 mL), and washed with brine (10 × 3 mL). The organic layer was dried (Na_2SO_4) and concentrated *in vacuo*. The residue was purified via flash chromatography (15 mL of SiO_2, 0–15% MeOH in $CHCl_3$ gradient elution) to afford **30** (2.5 mg, 0.0019 mmol, 61%) as a colorless oil: $[\alpha]^{25}_D$ –4.4 (*c* 0.25, MeOH); ^{1}H NMR (DMSO-d_6, 500 MHz) δ 9.48 (br s, 1H), 9.37 (s, 1H), 9.31 (s, 1H), 8.83 (br s, 1H), 8.29 (t, *J* = 5.9 Hz, 1H), 8.07 (d, *J* = 7.2 Hz, 1H), 8.00–7.95 (m, 1H), 7.91 (d, *J* = 9.2 Hz, 1H), 7.84 (d, *J* = 7.6 Hz, 1H), 7.67 (d, *J* = 9.4 Hz, 1H), 7.29–7.25 (m, 1H), 5.06 (br s, 1H), 5.02–4.93 (m, 2H), 4.42 (d, *J* = 9.4 Hz, 1H), 4.37 (t, *J* = 8.5 Hz, 1H), 4.28 (d, *J* = 8.0 Hz, 1H), 4.20–4.12 (m, 3H), 3.78–3.70 (m, 2H), 3.18–3.13 (m, 3H), 3.09 (dd, *J* = 7.2, 4.9 Hz, 1H), 2.89–2.83 (m, 1H), 2.81 (s, 3H), 2.80 (s, 3H), 2.66–2.64 (m, 1H), 2.38–2.36 (m, 1H), 2.35–2.27 (m, 2H), 2.18 (t, *J* = 7.4 Hz, 1H), 2.11–2.06 (m, 2H), 2.03–1.99 (m, 2H), 1.94 (br s, 6H), 1.88–1.84 (m, 1H), 1.71 (br s, 6H), 1.55–1.42 (m, 9H), 1.36 (s, 3H), 1.34 (s, 3H), 1.28 (s, 3H), 1.25 (s, 3H), 1.18 (s, 3H), 1.14 (s, 3H), 1.09 (s, 3H), 0.97–0.92 (m, 9H), 0.88–0.77 (m, 18H) ; ^{13}C NMR (DMSO-d_6, 125 MHz) δ 213.6, 175.1, 173.7, 173.32, 173.27, 172.8, 170.2, 165.2, 157.2, 156.2, 154.2, 147.4, 133.9, 130.1, 125.0, 123.9, 122.0, 116.0, 79.6, 73.2, 71.7, 70.2, 66.1, 60.7, 60.2, 59.5, 57.9, 56.5, 56.1, 49.2, 46.1, 43.1 (2C), 39.3, 34.6, 34.1, 31.8, 29.5, 29.4, 29.2, 29.0, 28.2, 28.1, 25.7, 25.2, 25.0, 24.4, 23.8, 22.6, 22.3, 21.9, 21.2, 20.7, 19.7, 18.8, 18.0, 17.5, 15.0, 14.4, 13.5, 12.4, 12.1, 9.0, 8.5; IR (film) ν_{max} 3296, 2918, 2798, 2352, 1765, 1702, 1655, 1610, 1546, 1450, 1387, 1215, 1090, 1036 cm^{-1}; HRMS (ESI) *m/z* 1289.8444 (MH$^+$, $C_{64}H_{112}N_{12}O_{15}H^+$ requires 1289.8443).

Description of Anticancer Assays

Cells (40 µL) are added into individual wells of a 384-well black/clear bottom plate on day zero at concentrations enabling continuous log growth over the course of the assay. On day one, compounds (or DMSO) are transferred using an ATS-liquid handler (100 nL per well) to individual wells containing cells and the cells are treated for 72 hours (37 °C, 5% CO_2) prior to measuring proliferation using MTS reagent following manufacturer's instructions. Net OD values are determined by subtracting the average OD values of cells (measured on day 1, T = 0) from average total OD value of cells (measured on day 4, T = 72h). IC_{50} values are calculated using proprietary software (IC_{50} values normalized against all compounds). Results represent n>2 independent studies (in duplicate).

Table S1. Antiproliferative Data of Compounds 1a, 2a, 2b, 23, 24, 26, and 30.

Compound	A549		HCT116		JURKAT		MCF7	
	Aver IC_{50} (nM)	Std Dev	Aver IC_{50} (nM)	Std Dev	Aver IC_{50} (nM)	Std Dev	Aver IC_{50} (nM)	Std Dev
**1a**	108.0	63.8	1,744.7	569.8	4,945.2	2,459.7	125.1	193.8
**2a**	86.5	46.1	1,127.8	246.4	4,210.8	1,194.3	13,012.8	13,854.0
**2b**	248.7	275.6	4,261.9	1,453.6	13,371.7	8,426.2	13,022.7	13,841.9
**23**	>25,000	0.0	>25,000	0.0	>25,000	0.0	19,185.5	11,629.0
**24**	>25,000	0.0	>25,000	0.0	>25,000	0.0	19,185.5	11,629.0
**26**	>25,000	0.0	>25,000	0.0	>25,000	0.0	6,343.8	12,438.5
**30**	20,648.1	6,215.9	>25,000	216.4	22,826.2	5,324.8	12,591.9	14,328.3
Paclitaxel	4.6	0.7	3.4	0.5	5.3	1.5	13.2	7.8
Camptothecin	27.7	6.0	10.9	2.7	7.3	0.6	13.2	7.8

Compound	MRC5		MV411		OVCAR3		RAMOS	
	Aver IC_{50} (nM)	Std Dev	Aver IC_{50} (nM)	Std Dev	Aver IC_{50} (nM)	Std Dev	Aver IC_{50} (nM)	Std Dev
**1a**	14,534.2	6,918.9	118.7	43.9	227.2	91.2	974.9	173.3
**2a**	5,469.2	604.5	20.8	9.6	81.4	34.4	361.7	64.4
**2b**	17,514.5	8,480.0	48.8	7.0	185.5	76.2	1,199.8	135.0
**23**	>25,000	0.0	>25,000	0.0	>25,000	0.0	>25,000	0.0
**24**	>25,000	0.0	>25,000	0.0	>25,000	0.0	>25,000	0.0
**26**	>25,000	0.0	>25,000	0.0	>25,000	0.0	>25,000	7,969.6

**30**	>25,000	0.0	2,702.1	1,131.6	12,643.6	3,408.0	24,322.3	911.9
Paclitaxel	8.8	2.2	4.4	0.2	3.0	0.6	5.9	2.1
Camptothecin	52.5	40.4	7.1	2.0	53.4	17.1	9.7	1.7

**Compound**	**THP1**		**U937**		**A375**		**BXPC3**	
	**Aver IC_{50} (nM)**	**Std Dev**	**Aver IC_{50} (nM)**	**Std Dev**	**Aver IC_{50} (nM)**	**Std Dev**	**Aver IC_{50} (nM)**	**Std Dev**
**1a**	9,289.9	7,479.8	1,209.6	272.4	2,136.4	1,625.9	3,816.6	789.3
**2a**	9,185.2	7,520.7	434.7	70.0	1,506.9	718.1	3,901.3	1,509.5
**2b**	9,685.9	7,089.5	947.3	691.0	254.2	74.0	9,310.0	4,017.8
**23**	>25,000	0.0	>25,000	0.0	>25,000	0.0	>25,000	0.0
**24**	>25,000	0.0	>25,000	0.0	>25,000	0.0	>25,000	0.0
**26**	>25,000	0.0	>25,000	0.0	>25,000	0.0	>25,000	0.0
**30**	>25,000	0.0	>25,000	0.0	24,742.5	477.6	>25,000	0.0
Paclitaxel	6.8	2.2	3.0	0.7	7.7	2.6	5.5	1.8
Camptothecin	44.9	22.2	19.7	2.1	5.9	2.9	38.4	21.8

**Compound**	**CALU6**		**COLO678**		**HL60**		**HT29**	
	**Aver IC_{50} (nM)**	**Std Dev**	**Aver IC_{50} (nM)**	**Std Dev**	**Aver IC_{50} (nM)**	**Std Dev**	**Aver IC_{50} (nM)**	**Std Dev**
**1a**	2,219.3	1,687.2	2,686.3	450.2	577.6	182.1	2,886.4	621.5
**2a**	578.0	700.6	2,008.8	543.5	370.4	224.9	2,271.9	127.9
**2b**	1,003.0	885.3	6,501.7	2,024.8	465.0	82.3	5,254.0	1,484.0
**23**	>25,000	0.0	>25,000	0.0	>25,000	0.0	>25,000	0.0
**24**	>25,000	0.0	>25,000	0.0	>25,000	0.0	>25,000	0.0
**26**	>25,000	0.0	>25,000	0.0	>25,000	0.0	>25,000	0.0
**30**	>25,000	0.0	>25,000	0.0	20,025.5	5,602.3	>25,000	0.0
Paclitaxel	3.6	0.3	24.0	31.3	9.7	4.7	6.0	2.7
Camptothecin	10.2	3.0	125.5	102.1	21.0	8.6	103.1	76.1

**Compound**	**NCIH1048**		**PANC1005**		**SNUC1**	
	**Aver IC_{50} (nM)**	**Std Dev**	**Aver IC_{50} (nM)**	**Std Dev**	**Aver IC_{50} (nM)**	**Std Dev**
**1a**	3,110.4	1,222.3	2,832.8	419.7	129.1	26.4
**2a**	1,307.0	30.4	1,967.4	839.2	34.4	12.2
**2b**	2,366.1	664.7	8,813.2	2,318.1	58.3	23.6
**23**	>25,000	0.0	>25,000	0.0	>25,000	0.0
**24**	>25,000	0.0	>25,000	0.0	24,871.8	362.7
**26**	>25,000	0.0	>25,000	0.0	>25,000	0.0
**30**	>25,000	0.0	>25,000	0.0	20,551.2	6,920.0

Paclitaxel	4.7	0.4	7.4	9.4	26.5	14.2
Camptothecin	9.7	4.4	45.3	61.3	47.4	16.9

Table S2. Human Cell Line Descriptions.

Human Cell Line*	Description
A375	Malignant Melanoma
A549	Lung - NSCLC
BXPC3	Pancreatic - Adenocarcinoma
CALU6	Lung - Adenocarcinoma
COLO678	Colon Carcinoma
HCT116	Colon Carcinoma
HL60	Leukema - Promyelocytic
HT29	Colon Carcinoma
JURKAT	T-cell - acute T-cell leukemia
MCF7	Breast Carcinoma
MRC5	Lung Fibroblast
MV411	Acute Myeloid Leukemia
NCIH1048	Lung - SCLC
OVCAR3	Ovary - Adenocarcinoma
PANC1005	Pancreatic - Adenocarcinoma
RAMOS	Burkitt's lymphoma
SNUC1	Colon Carcinoma
THP1	Monocyte-like - Acute Monocytic Leukemia
U937	Pro-monocytic, Human Myeloid Leukemia

*All cells were obtained from ATCC and are authenticated

Computational Data

Table S3. AMBER Partial Charges for the *N*-Terminal Acyl Group. Results of the R.E.D. RESP partial charge calculations for the *N*-terminal acyl group. Stereobonds are used where the output was otherwise ambiguous.

***N*-Terminal Acyl Group (NTA)**

Ref.	Type	Charge
1	C	0.5542
2	O	-0.5455
3	CT	0.0459
4	CT	-0.0869
5	HC	0.0369
6	HC	0.0369
7	HC	0.0369
8	CT	-0.2884
9	HC	0.0704
10	HC	0.0704
11	HC	0.0704
12	C	0.4655
13	O	-0.4947
14	CT	0.0658
15	HC	0.0031
16	CT	-0.2482
17	HC	0.0692
18	HC	0.0692
19	HC	0.0692
20	CT	-0.1398
21	HC	0.0396
22	HC	0.0396
23	CT	0.2324
24	HC	-0.0060
25	CT	-0.1839
26	HC	0.0293
27	HC	0.0293
28	HC	0.0293
29	CT	-0.1244
31	HC	0.0181
31	HC	0.0181
32	HC	0.0181
**Total**		0.0000

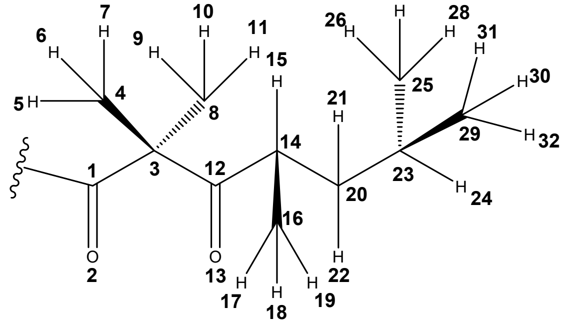

Table S4. AMBER Partial Charges for β-Hydroxyisoleucine. Results of the R.E.D. RESP partial charge calculations for β-hydroxyisoleucine. Stereobonds are used where the output was otherwise ambiguous.

Hydroxyisoleucine (β-OHILE)

Ref.	Type	Charge
1	C	0.4444
2	O	-0.5203
3	CT	-0.0431
4	H1	0.0593
5	CT	0.2817
6	CT	-0.1480
7	HC	0.0460
8	HC	0.0460
9	HC	0.0460
10	OH	-0.6284
11	HC	0.3977
12	CT	0.0189
13	HC	-0.0060
14	HC	-0.0060
15	CT	-0.0428
16	HC	0.0159
17	HC	0.0159
18	HC	0.0159
19	N	-0.2302
20	H	0.2371
**Total**		0.0000

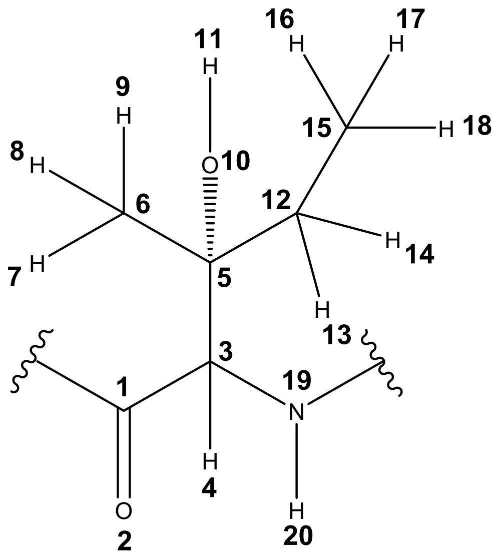

Table S5. AMBER Partial Charges for Dehydroisoleucine. Results of the R.E.D. RESP partial charge calculations for (*Z*)-Dehydroisoleucine. Stereobonds are used where the output was otherwise ambiguous.

(*Z*)-Dehydroisoleucine (*Z*-ΔILE)

Ref.	Type	Charge
1	C	0.5621
2	O	-0.5591
3	CM	-0.1089
4	CM	0.0739
5	CT	-0.2263
6	HC	0.0872
7	HC	0.0872
8	HC	0.0872
9	CT	-0.0358
10	HC	0.0456
11	HC	0.0456
12	CT	-0.0905
13	HC	0.0294
14	HC	0.0294
15	HC	0.0294
16	N	-0.3673
17	H	0.3109
**Total**		0.0000

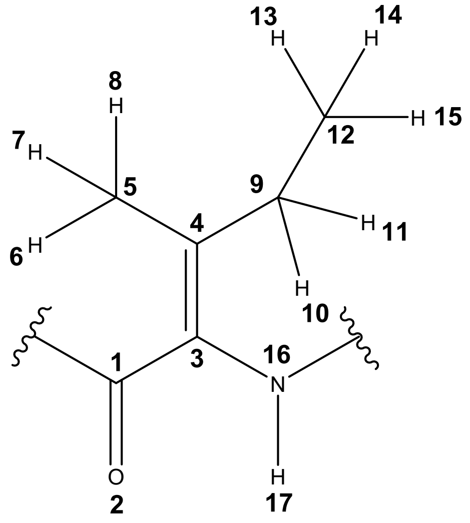

Table S6. AMBER Partial Charges for (*E*)-Dehydroisoleucine. Results of the R.E.D. RESP partial charge calculations for (*E*)-Dehydroisoleucine. Stereobonds are used where the output was otherwise ambiguous.

(*E*)-Dehydroisoleucine (*E*-ΔILE)

Ref.	Type	Charge
1	C	0.5852
2	O	-0.5634
3	CM	-0.2488
4	CM	0.0909
5	CT	-0.0166
6	HC	0.0379
7	HC	0.0379
8	CT	-0.0204
9	HC	0.0107
10	HC	0.0107
11	HC	0.0107
12	CT	-0.1016
13	HC	0.0444
14	HC	0.0444
15	HC	0.0444
16	N	-0.2323
17	H	0.2659
**Total**		0.0000

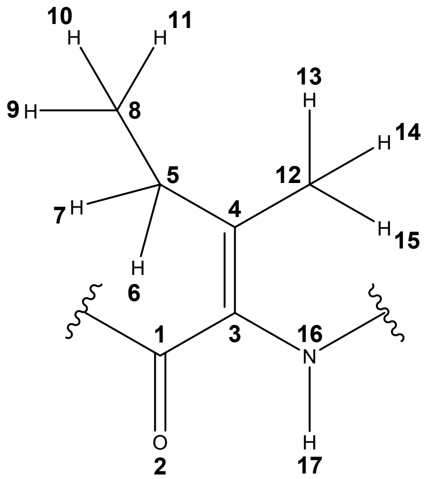

Table S7. AMBER Partial Charges for β-Hydroxyvaline. Results of the R.E.D. RESP partial charge calculations for hydroxyvaline. Stereobonds are used where the output was otherwise ambiguous.

β-Hydroxyvaline (β-OHVAL)

**Ref.**	**Type**	**Charge**
1	C	0.5959
2	O	-0.5575
3	C	-0.0130
4	H	0.1044
5	C	0.2615
6	C	-0.1277
7	H	0.0408
8	H	0.0408
9	H	0.0408
10	O	-0.7079
11	H	0.4541
12	C	-0.1843
13	H	0.0517
14	H	0.0517
15	H	0.0517
16	N	-0.3346
17	H	0.2316
**Total**		0.0000

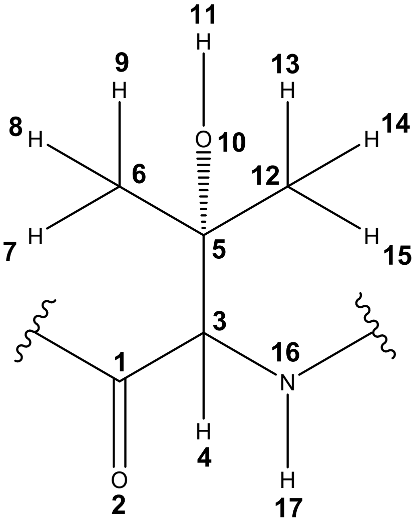

Table S8. AMBER Partial Charges for Dehydrovaline. Results of the R.E.D. RESP partial charge calculations for dehydrovaline. Stereobonds are used where the output was otherwise ambiguous.

Dehydrovaline (ΔVAL)

Ref.	Type	Charge
1	C	0.7785
2	O	-0.6029
3	CM	-0.2929
4	CM	0.1206
5	CT	-0.2080
6	HC	0.0652
7	HC	0.0652
8	HC	0.0652
9	CT	-0.1418
10	HC	0.0642
11	HC	0.0642
12	HC	0.0642
13	N	-0.2955
14	H	0.2538
**Total**		0.0000

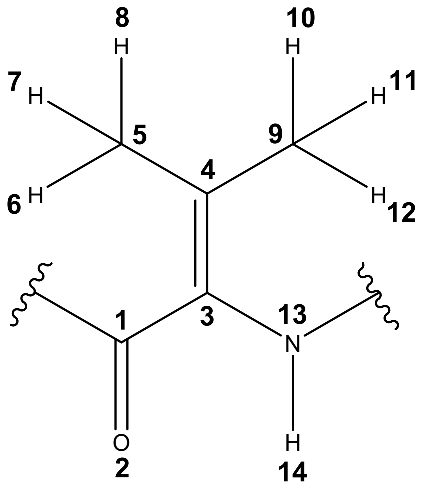

Table S9. AMBER Partial Charges for Dehydroethylnorvaline. Results of the R.E.D. RESP partial charge calculations for dehydroethylnorvaline. Stereobonds are used where the output was otherwise ambiguous.

Dehydroethylnorvaline (ΔEnv)

Ref.	Type	Charge
1	C	0.5958
2	O	-0.5687
3	CM	-0.1596
4	CM	-0.0116
5	CT	-0.0059
6	HC	0.0499
7	HC	0.0499
8	CT	-0.0348
9	HC	0.0111
10	HC	0.0111
11	HC	0.0111
12	CT	-0.0318
13	HC	0.0587
14	HC	0.0587
15	CT	-0.1276
16	HC	0.0375
17	HC	0.0375
18	HC	0.0375
19	N	-0.3280
20	H	0.3092
**Total**		0.0000

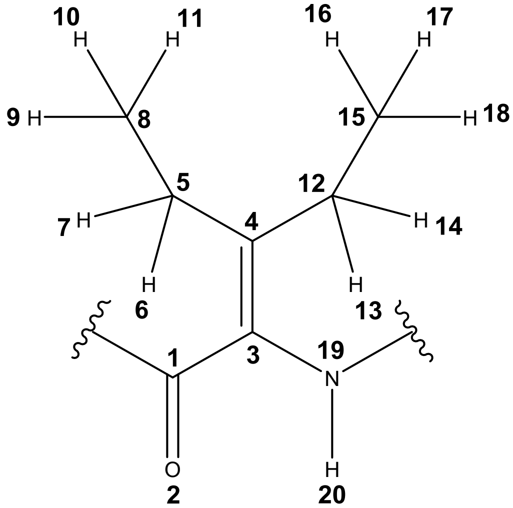

Table S10. Amber Partial Charges for the *C*-Terminal Amine. Results of the R.E.D. RESP partial charge calculations for the *C*-terminal amine. Stereobonds are used where the output was otherwise ambiguous.

***C*-Terminal Amine (CTA)**

Ref.	Type	Charge
1	N	-0.4859
2	H	0.2983
3	CT	0.0106
4	H1	0.1642
5	CT	0.2061
6	HC	-0.0089
7	CT	-0.1229
8	HC	0.0209
9	HC	0.0209
10	HC	0.0209
11	CT	-0.2487
12	HC	0.0579
13	HC	0.0579
14	HC	0.0579
15	CT	-0.2269
16	H1	0.1146
17	H1	0.1146
18	N3	-0.2375
19	CT	-0.0988
20	H1	0.0663
21	H1	0.0663
22	H1	0.0663
23	CT	-0.1607
24	H1	0.0822
25	H1	0.0822
26	H1	0.0822
**Total**		0.0000

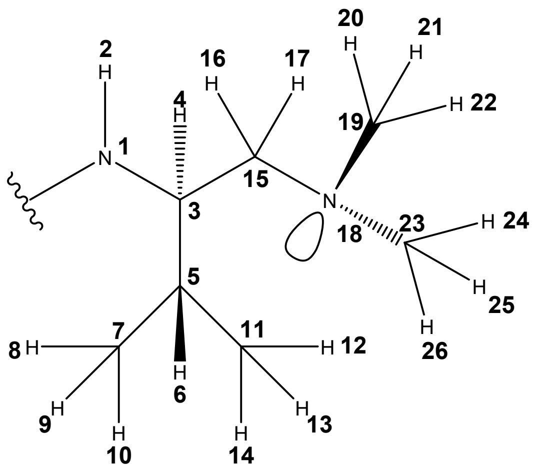

Table S11. Bond Stretch Function Definition. The obtained AMBER force constant (N) and equilibrium bond length (R) parameters for the indicated atoms.

Type	Atom Types	N	R
HrmStr1	N–CM	400.10	1.399
HrmStr1	H1–C	340.00	1.08

Table S12. Angle Bend Function Definition. The obtained AMBER force constant (N) and equilibrium bond angle (θ) parameters for the indicated atoms.

Type	Atom 1	N	θ
HrmBnd1	C–CT–C	63.380	111.63
HrmBnd1	CT–C–CT	62.040	116.50
HrmBnd1	C–N–CM	64.53	122.15
HrmBnd1	N–CM–CM	68.71	123.67
HrmBnd1	N–CM–C	69.192	116.055
HrmBnd1	H–N–CM	47.63	117.90
HrmBnd1	CM–C–N	69.957	115.17
HrmBnd1	CM–CT–CT	63.41	111.56
HrmBnd1	CT–CM–CT	62.87	115.65
HrmBnd1	H1–C–N	50.0	109.50
HrmBnd1	H1–C–O	50.00	109.50

Table S13. Torsional Angle Function Definition. The obtained AMBER parameters for the torsional angles. The required values include four atoms, one to four phase offsets (PO), V/2 magnitudes (Mag), and the number of paths (NPaths).

Type	Atom Types	PO 1	PO2	PO3	PO4	Mag 1	Mag 2	Mag 3	Mag 4	N P
AmbTrs	C–CT–C–O	0	180	0	0	0	0	0	0	1
AmbTrs	C–CT–C–CT	0	180	0	0	0	0	0	0	1
AmbTrs	C–CT–CT–HC	0	0	0	0	0	0	0.2	0	1
AmbTrs	O–C–CT–CT	0	180	0	0	0	0	0	0	1
AmbTrs	O–C–N–H	0	180	0	0	2	2.5	0	0	1
AmbTrs	CT–C–N–H	0	180	0	0	0	2.5	0	0	1
AmbTrs	CT–C–CT–HC	0	180	0	0	0	0	0	0	1
AmbTrs	CT–C–CT–CT	0	180	0	0	0	0	0	0	1

AmbTrs	C–CT–C–N	0	180	0	0	0	0	0	0	1
AmbTrs	C–CT–CT–CT	0	0	0	0	0	0	0.2	0	1
AmbTrs	O–C–CT–HC	0	0	180	0	0.8	0	0.1	0	1
AmbTrs	CT–CT–CT–HC	0	0	0	0	0	0	0.2	0	1
AmbTrs	CT–CT–CT–CT	180	180	0	0	0.2	0.3	0.2	0	1
AmbTrs	HC–CT–CT–HC	0	0	0	0	0	0	0.2	0	1
AmbTrs	C–N–CT–H1	0	0	0	0	0	0	0	0	1
AmbTrs	C–N–CT–CT	0	0	180	180	0.5	0	0.2	0.5	1
AmbTrs	O–C–N–CT	0	180	0	0	0	2.5	0	0	1
AmbTrs	N–CT–CT–CT	0	0	0	0	0	0	0.2	0	1
AmbTrs	N–CT–CT–OH	0	0	0	0	0	0	0.2	0	1
AmbTrs	N–CT–C–O	0	180	0	0	0	0	0	0	1
AmbTrs	H–N–CT–H1	0	0	0	0	0	0	0	0	1
AmbTrs	H–N–CT–CT	0	0	0	0	0	0	0	0	1
AmbTrs	H–N–CT–C	0	0	0	0	0	0	0	0	1
AmbTrs	CT–CT–OH–HO	0	0	0	0	0.3	0	0.2	0	1
AmbTrs	H1–CT–CT–CT	0	0	0	0	0	0	0.2	0	1
AmbTrs	H1–CT–CT–OH	0	0	0	0	0.3	0	0	0	1
AmbTrs	H1–CT–C–O	0	0	180	0	0.8	0	0.1	0	1
AmbTrs	H1–CT–C–N	0	180	0	0	0	0	0	0	1
AmbTrs	CT–CT–C–O	0	180	0	0	0	0	0	0	1
AmbTrs	C–CT–CT–OH	0	0	0	0	0	0	0.2	0	1
AmbTrs	HC–CT–CT–OH	0	0	0	0	0.3	0	0	0	1
AmbTrs	CT–CT–CT–OH	0	0	0	0	0	0	0.2	0	1
AmbTrs	C–N–CM–CM	180	180	0	0	1.2	0.7	0	0	1
AmbTrs	C–N–CM–C	0	180	0	0	0	0.7	0	0	1
AmbTrs	O–C–N–CM	0	180	0	0	0	2.5	0	0	1
AmbTrs	N–CM–CM–CT	0	180	0	0	0	6.7	0	0	1
AmbTrs	N–CM–C–O	0	180	0	0	0	2.2	0	0	1

AmbTrs	N–CM–C–N	0	180	0	0	0	2.2	0	0	1
AmbTrs	H–N–CM–CM	0	180	0	0	0	0.7	0	0	1
AmbTrs	H–N–CM–C	0	180	0	0	0	0.7	0	0	1
AmbTrs	CM–CM–CT–HC	0	0	180	0	1.2	0	0.4	0	1
AmbTrs	CM–CM–CT–CT	0	0	0	0	0	0	0	0	1
AmbTrs	CM–C–N–H	0	180	0	0	0	2.5	0	0	1
AmbTrs	CM–CM–C–O	0	180	0	0	0	2.2	0	0	1
AmbTrs	CM–CM–C–N	0	180	0	0	0	2.2	0	0	1
AmbTrs	CM–CT–CT–HC	0	0	0	0	0	0	0.2	0	1
AmbTrs	CT–CM–CT–HC	0	0	0	0	0	0	0	0	1
AmbTrs	CT–CM–CT–CT	0	0	0	0	0	0	0	0	1
AmbTrs	N–CT–CT–H1	0	0	0	0	0	0	0.2	0	1
AmbTrs	N–CT–CT–N3	0	0	0	0	0	0	0.2	0	1
AmbTrs	N–CT–CT–HC	0	0	0	0	0	0	0.2	0	1
AmbTrs	CT–CT–N3–CT	0	0	0	0	0	0	0.2	0	1
AmbTrs	H1–CT–CT–H1	0	0	0	0	0	0	0.2	0	1
AmbTrs	H1–CT–CT–N3	0	0	0	0	0	0	0.2	0	1
AmbTrs	H1–CT–CT–HC	0	0	0	0	0	0	0.2	0	1
AmbTrs	CT–N3–CT–H1	0	0	0	0	0	0	0.2	0	1
AmbTrs	N3–CT–CT–CT	0	0	0	0	0	0	0.2	0	1

Table S14. Applied ONIOM Methods. Shows each of the analogs, the ONIOM partitioning, and the model chemistries that were used to optimize the structures.

Analogs	ONIOM Partitioning	Model Chemistry
Parent: YA	84:177	
Analog 1: VVV	75:177	
Analog 2: VVE	81:177	
Analog 3: VEE	87:177	B3LYP/d95(d,p):AMBER
Analog 4: EEE	93:177	B3LYP/6-311g(d,p):AMBER
Analog 5: EEV	87:177	M06-2X/6-31+G(d):AMBER
Analog 6: EVV	81:177	
Analog 7: EVE	87:177	
Analog 8: VEV	81:177	

Table S15. RMSD Comparision of All Heavy Atoms. Extra methyl groups were removed so that all structures had the same number of atoms. All methods used AMBER for the low layer. Values are calculated in angstroms.

	**AMBER**	**B3LYP/ d95(d,p)**	**B3LYP/ 6-311g(d,p)**	**M06-2X/ 6-31+G(d)**
**1: VVV**	0.1112	1.3861	1.4055	1.3892
**2: VVE**	0.4772	0.9712	1.0010	1.4828
**3: VEE**	0.4595	1.5816	1.6535	0.9689
**4: EEE**	0.3525	1.1119	1.1771	0.7729
**5: EEV**	0.1936	1.4367	1.3862	1.1808
**6: EVV**	0.1034	0.4241	0.2036	1.3060
**7: EVE**	0.2592	1.5890	1.5544	1.2500
**8: VEV**	0.4772	1.5890	0.6943	0.8822

Table S16. RMSD Comparison for Backbone Atoms Only. Calculated RMSDs from the backbone for each analog in angstroms.

	AMBER	B3LYP/ d95(d,p)	B3LYP/ 6-311g(d,p)	M06-2X/ 6-31+G(d)
**1: VVV**	0.0663	0.8276	0.8454	1.0020
**2: VVE**	0.2999	0.7467	0.7623	1.1194
**3: VEE**	0.3728	0.8591	0.9706	0.7261
**4: EEE**	0.2585	0.6692	0.6963	0.5618
**5: EEV**	0.2585	0.8452	0.7453	0.8478
**6: EVV**	0.0847	0.8452	0.1289	0.8517
**7: EVE**	0.1515	0.8630	1.0237	0.7567
**8: VEV**	0.1248	0.8297	0.4115	0.5895

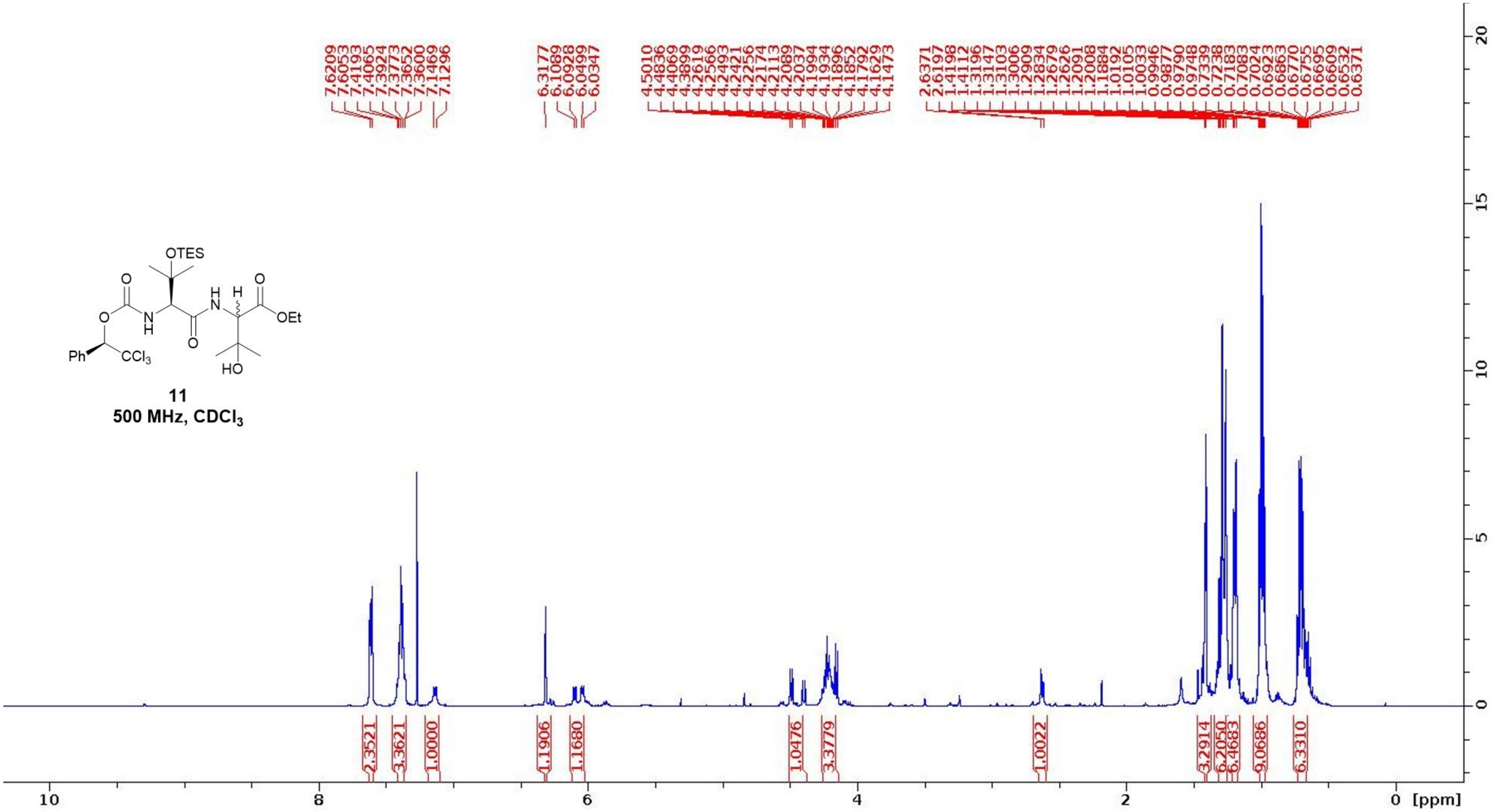

11

500 MHz, $CDCl_3$

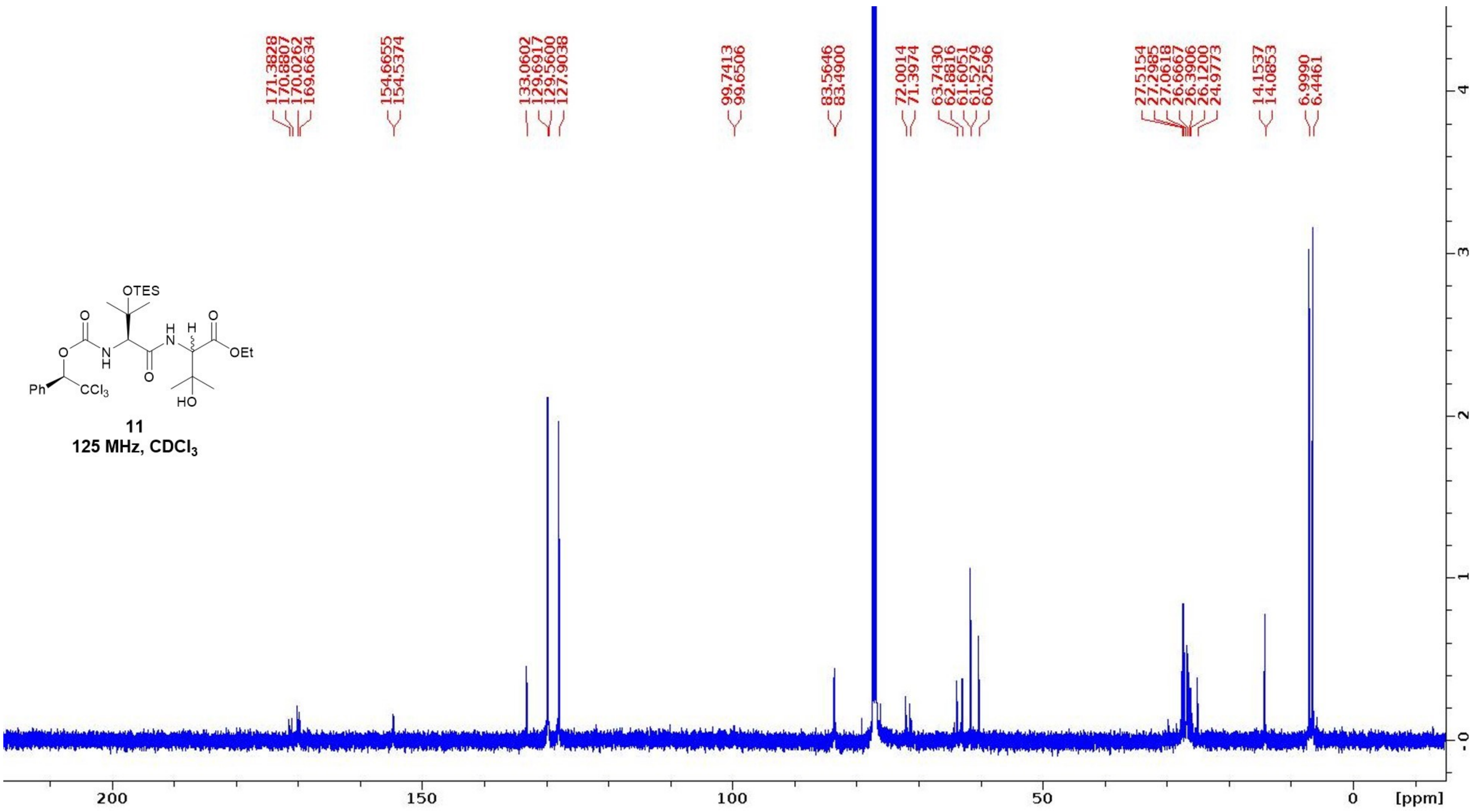

11
125 MHz, $CDCl_3$

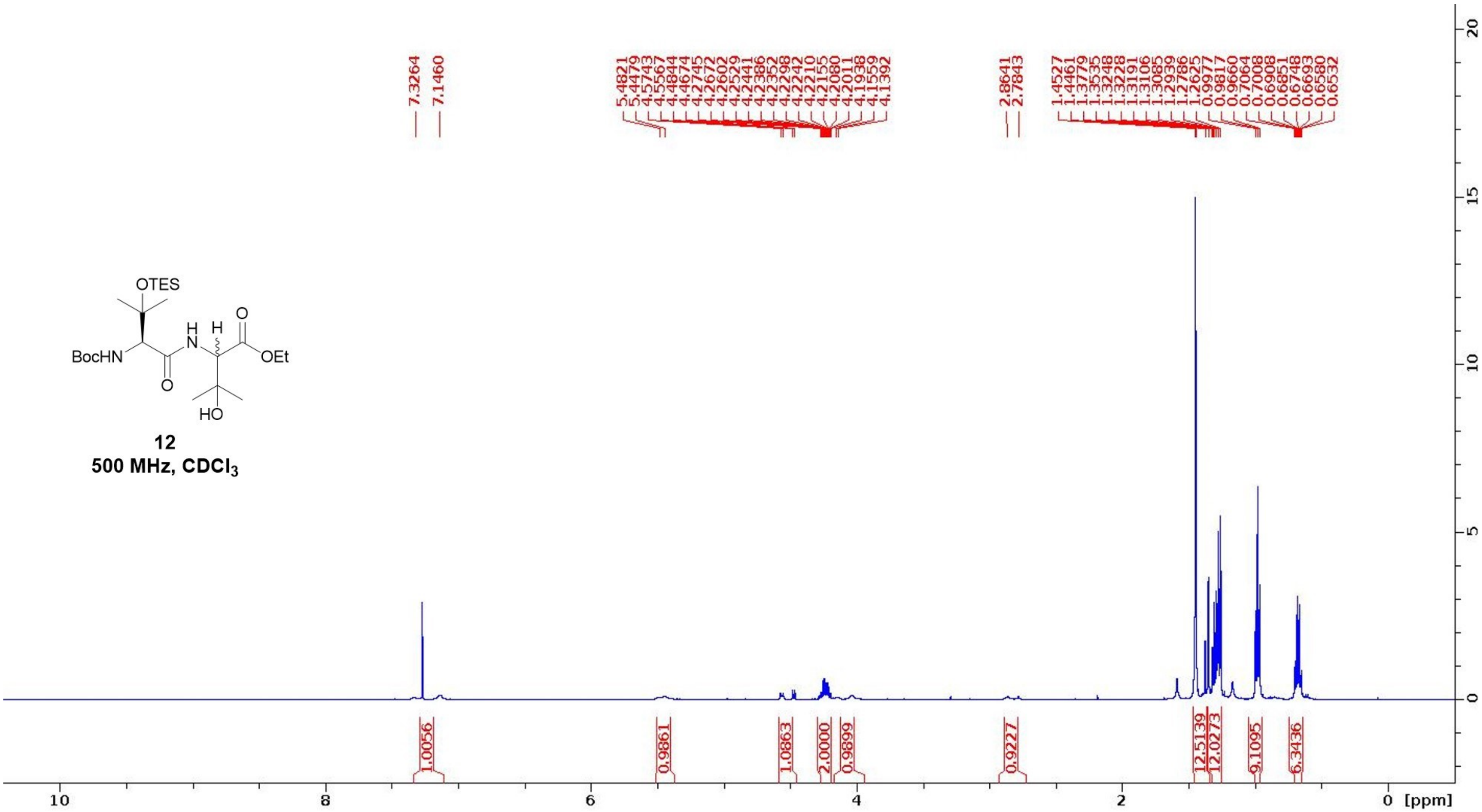

12
500 MHz, CDCl$_3$

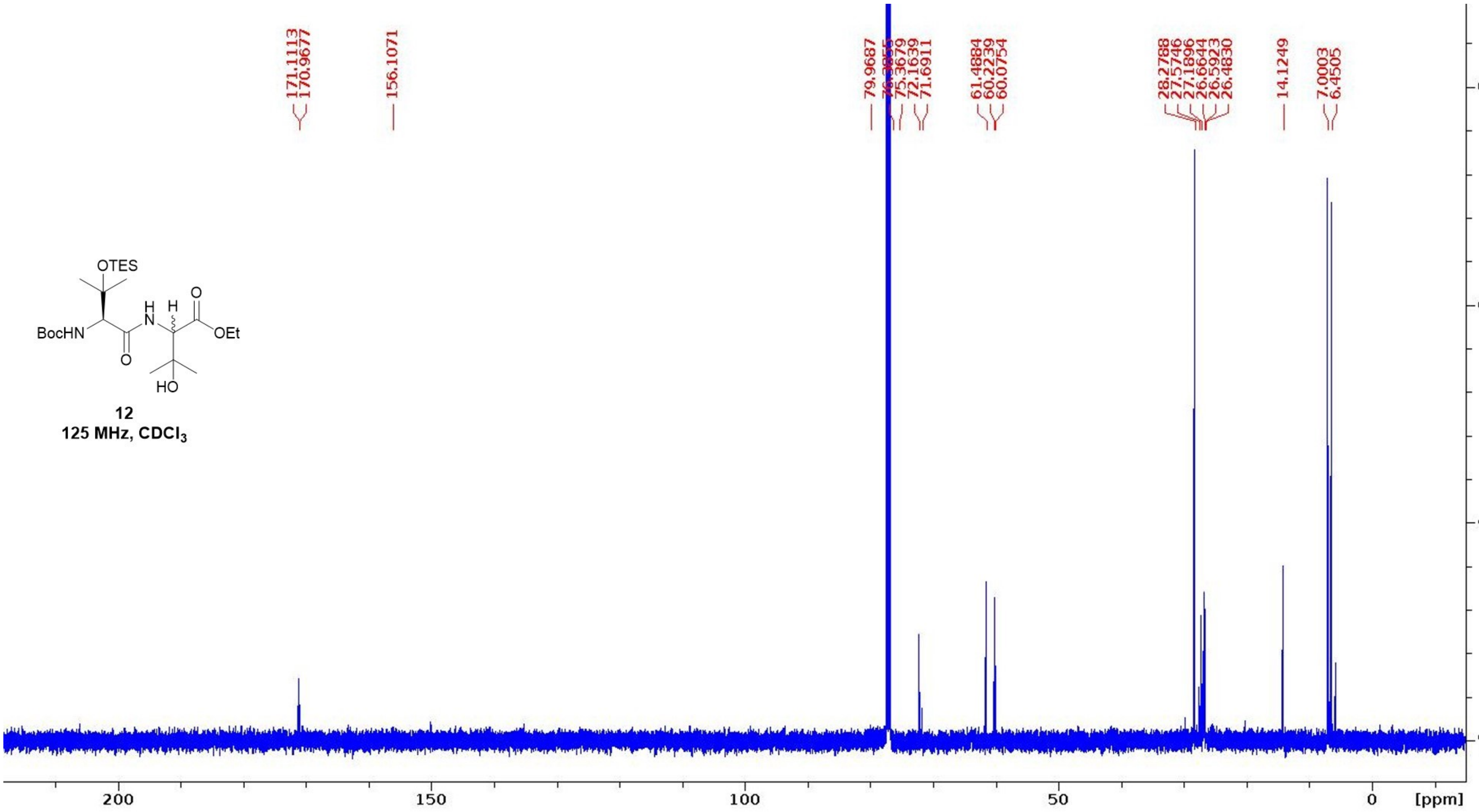
171.1113
170.9677
156.1071
79.9687
75.3679
72.1639
71.6911
61.4884
60.2239
60.0754
28.2788
27.5746
27.1896
26.6644
26.5923
26.4830
14.1249
7.0003
6.4505
OTES
BocHN
H
N
H
O
OEt
O
HO
12
125 MHz, $CDCl_3$
200
150
100
50
0
[ppm]
3
2
1
0

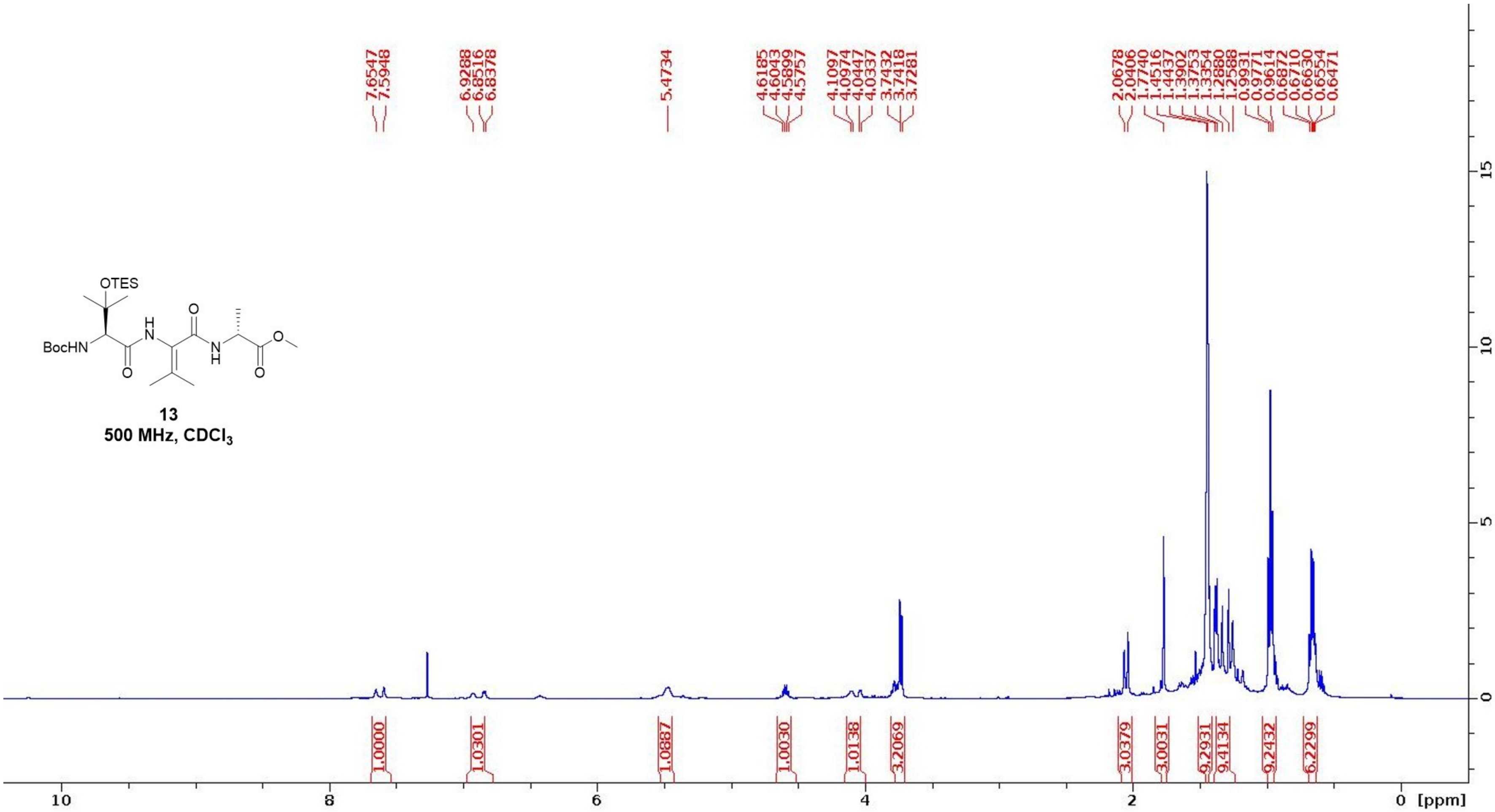

13
500 MHz, $CDCl_3$

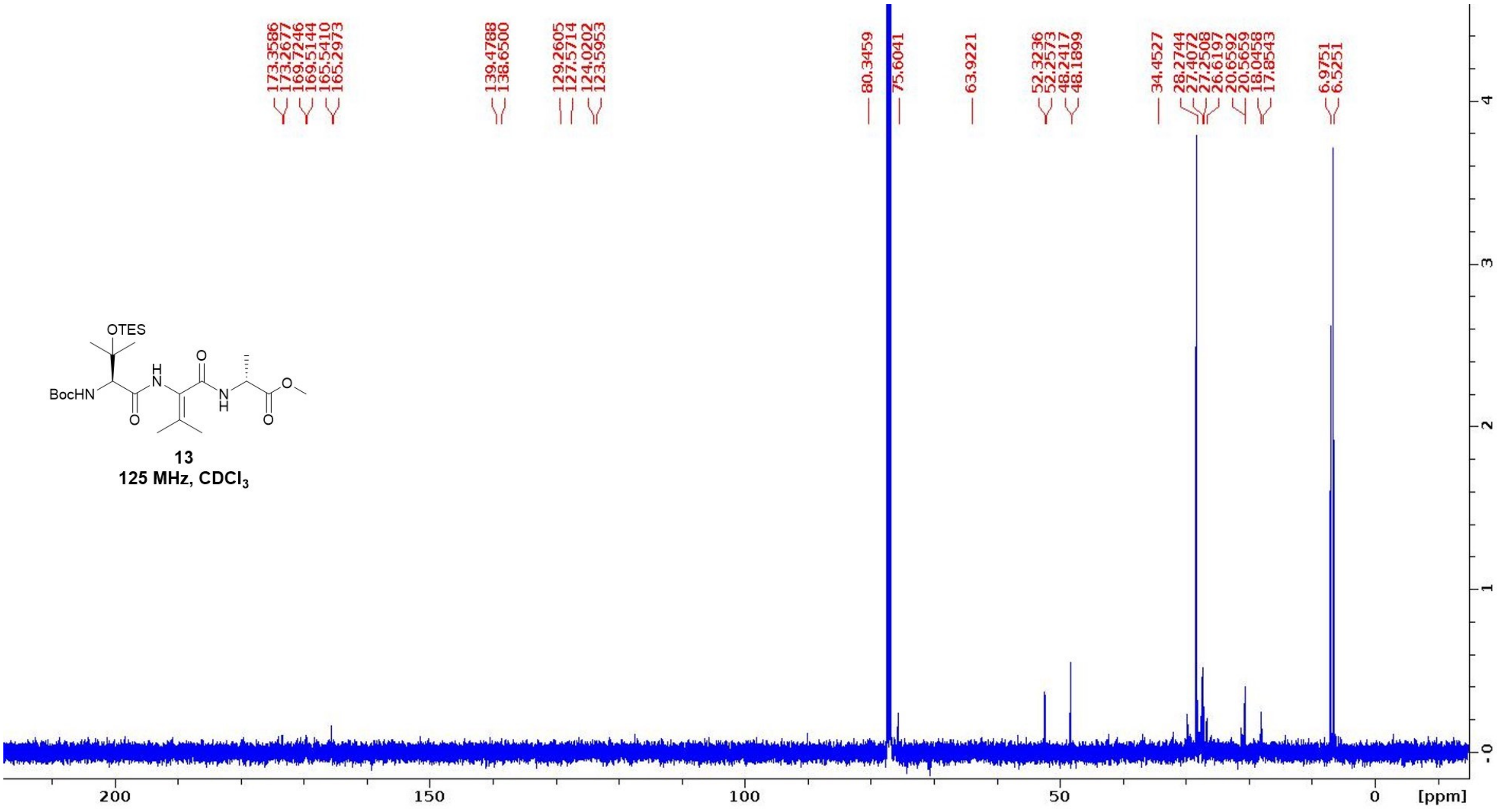

13
125 MHz, $CDCl_3$

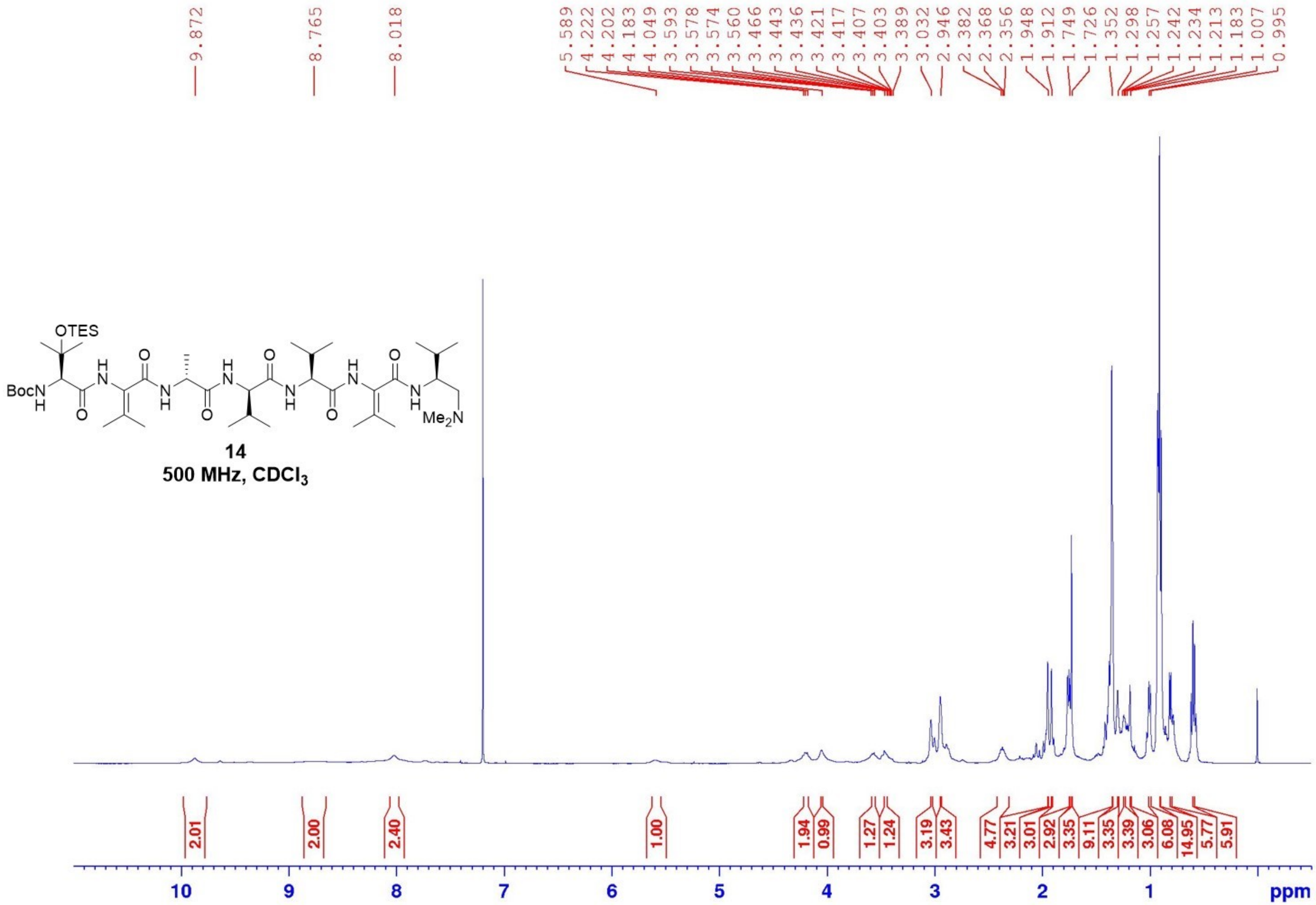

14
500 MHz, $CDCl_3$

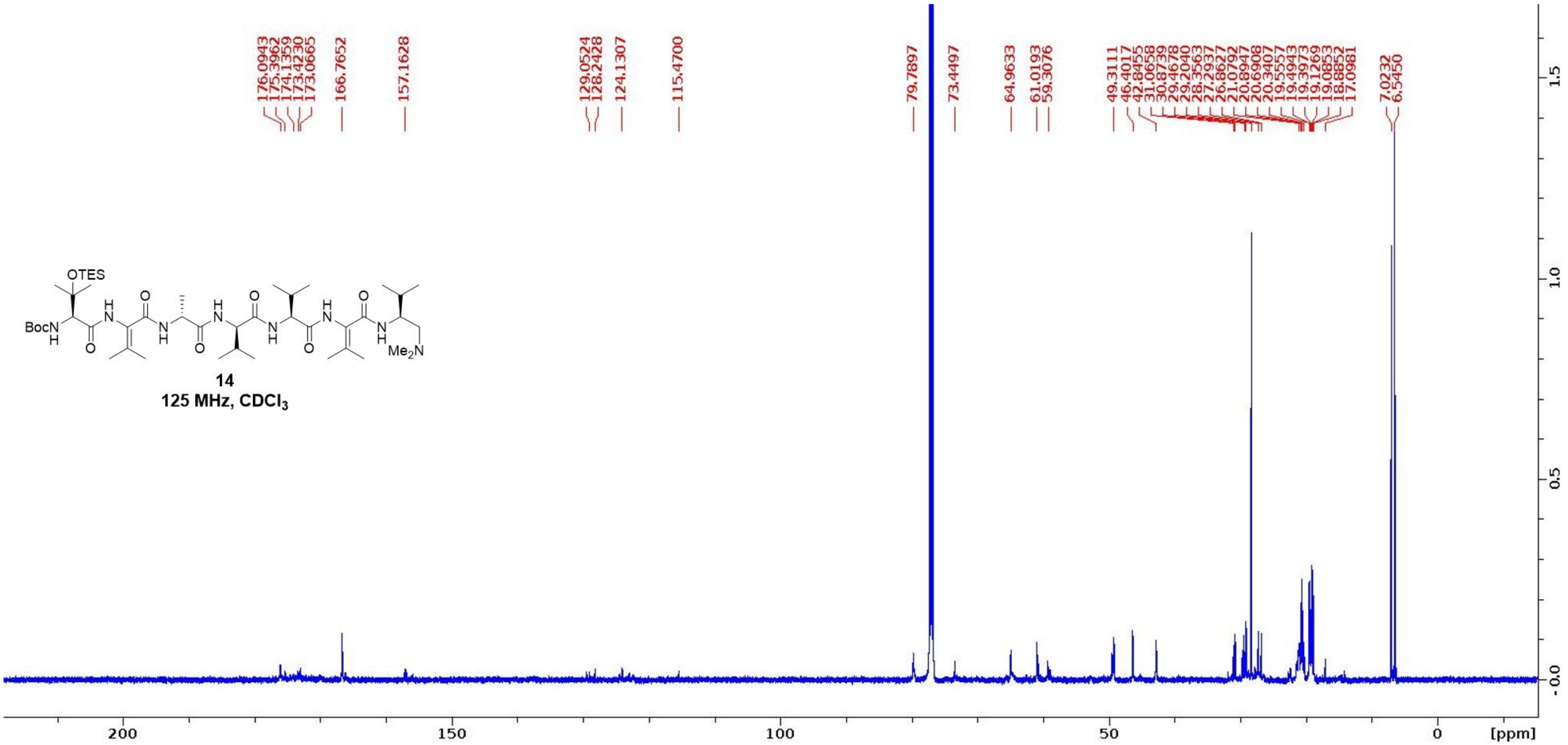

14
125 MHz, CDCl$_3$

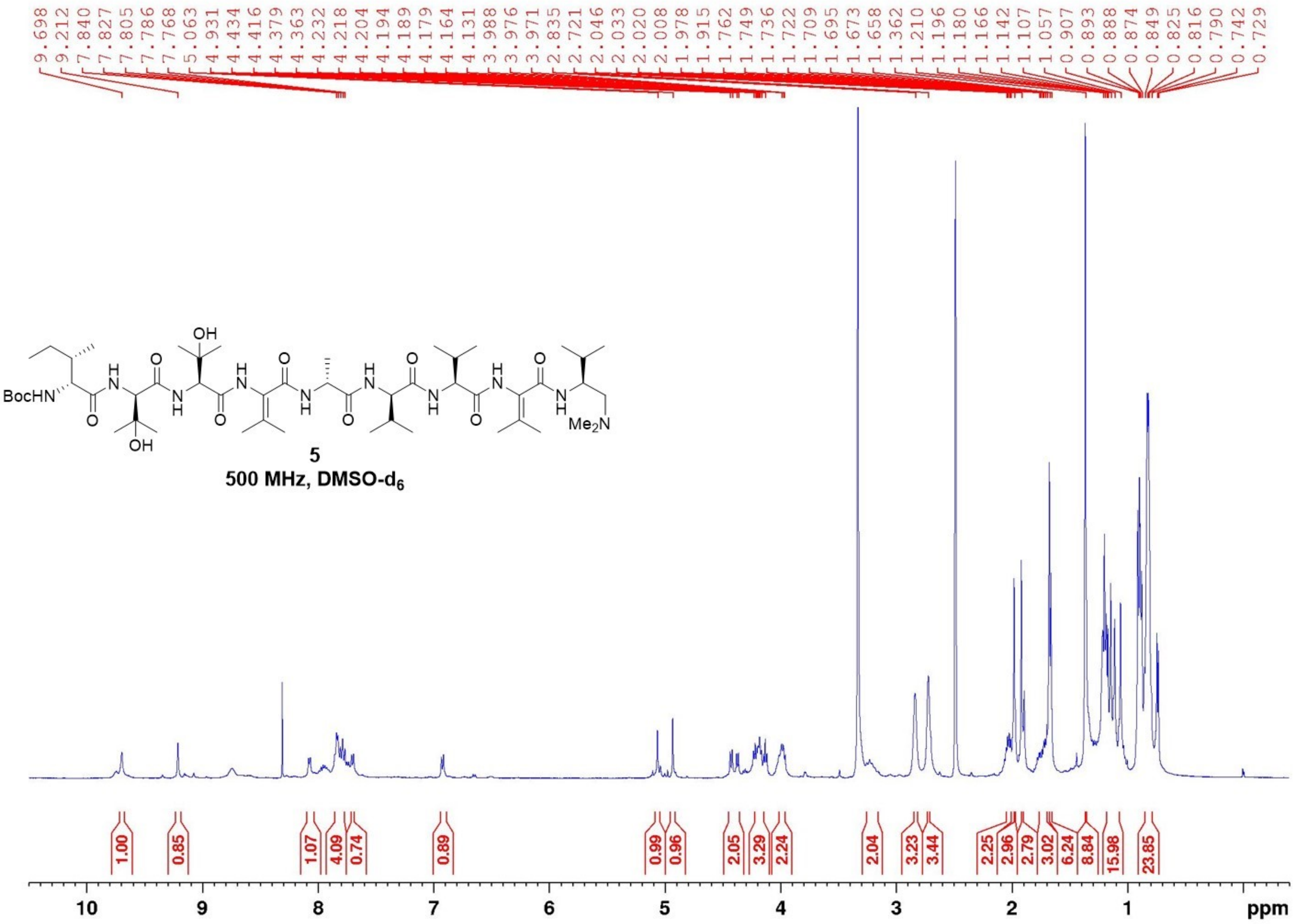

5

500 MHz, DMSO-d$_6$

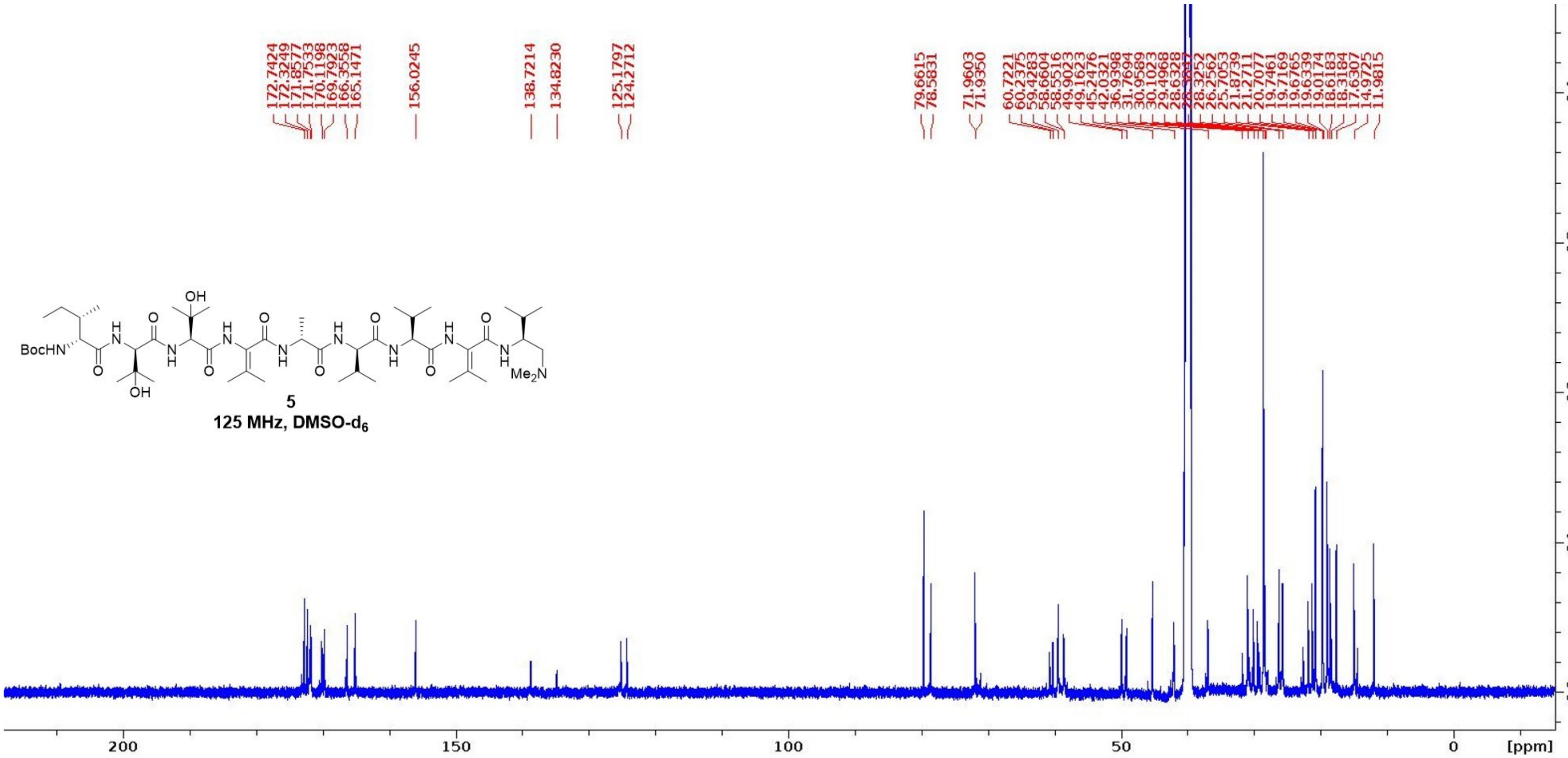
172.7424
172.3249
171.8577
171.7533
170.1198
169.7923
166.3558
165.1471
156.0245
138.7214
134.8230
125.1797
124.2712
79.6615
78.5831
71.9603
71.9350
60.7221
60.2375
59.4283
58.6604
58.5516
49.9023
49.1623
45.2476
42.0321
36.9398
31.7694
30.9589
30.1023
29.4968
28.6328
28.3252
26.2562
25.7053
21.8739
21.2711
20.7077
19.7461
19.7169
19.6765
19.6339
19.0174
18.6183
18.3184
17.6307
14.9725
11.9815
BocHN
OH
NH
O
Me2N
5
125 MHz, DMSO-d6
200
150
100
50
0
[ppm]
0.0
0.1
0.2
0.3
0.4

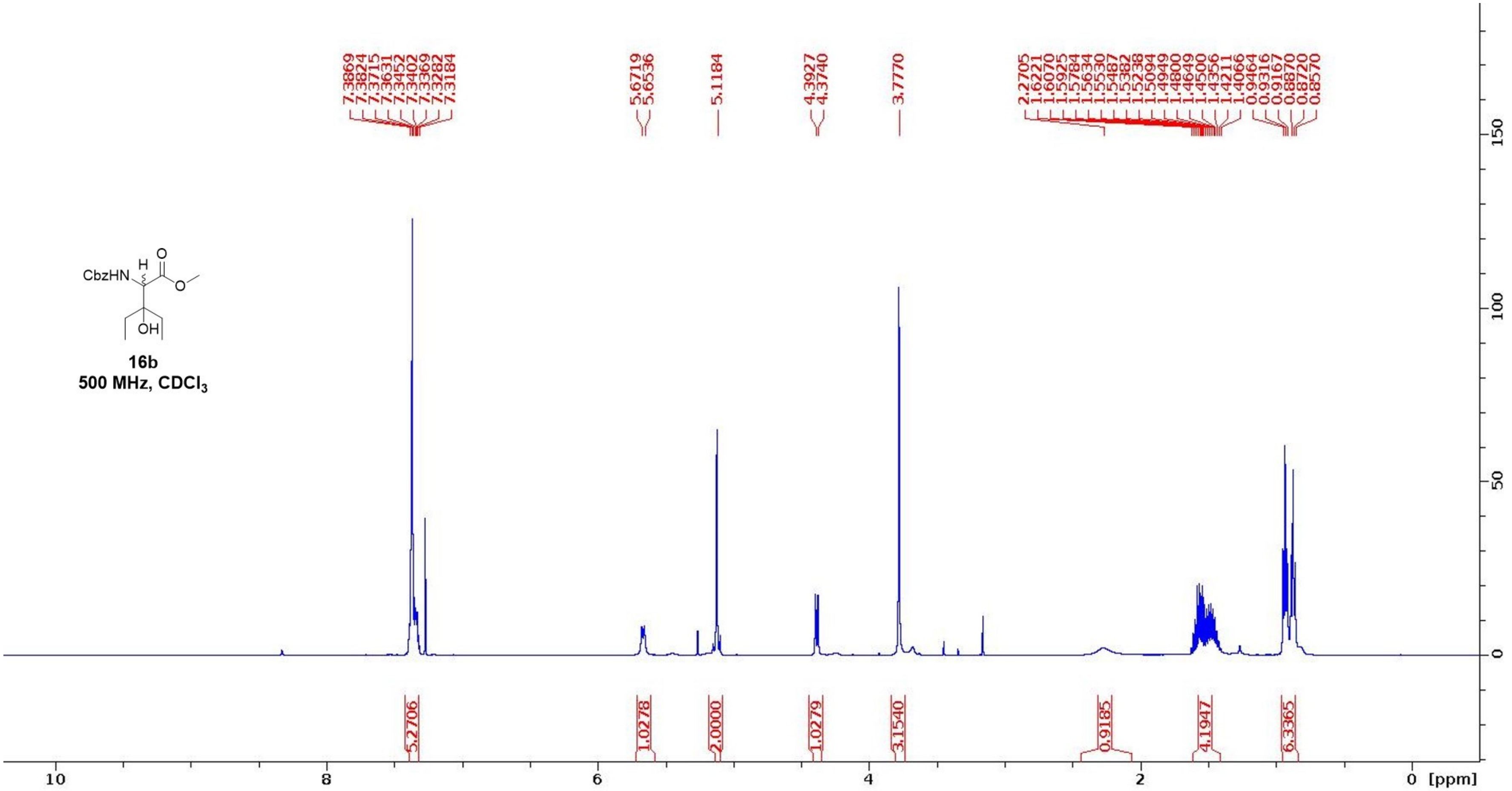

16b
500 MHz, $CDCl_3$

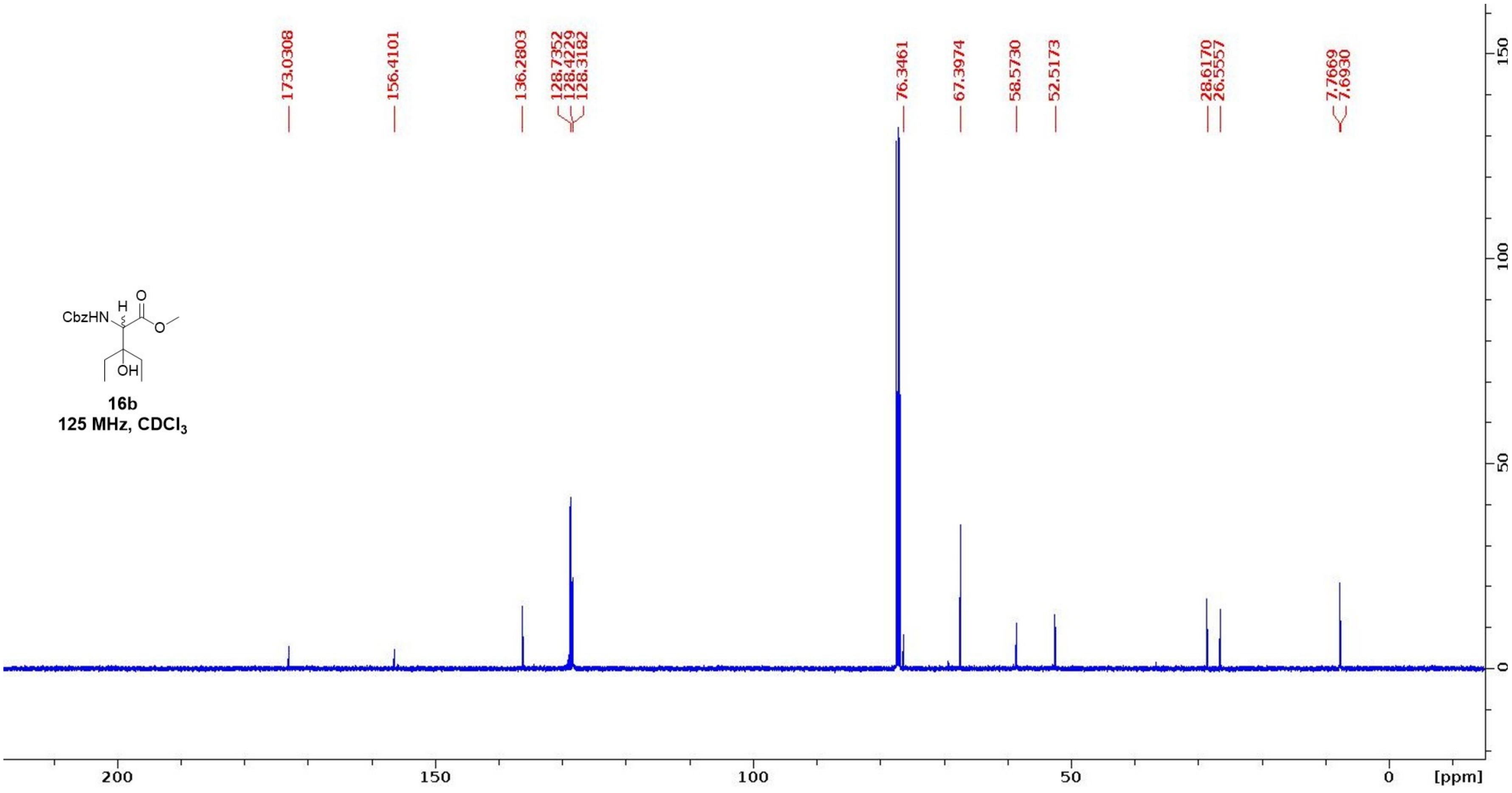
173.0308
156.4101
136.2803
128.7352
128.4229
128.3182
76.3461
67.3974
58.5730
52.5173
28.6170
26.5557
7.7669
7.6930
CbzHN
H
O
O
OH
16b
125 MHz, CDCl3
200
150
100
50
0
[ppm]

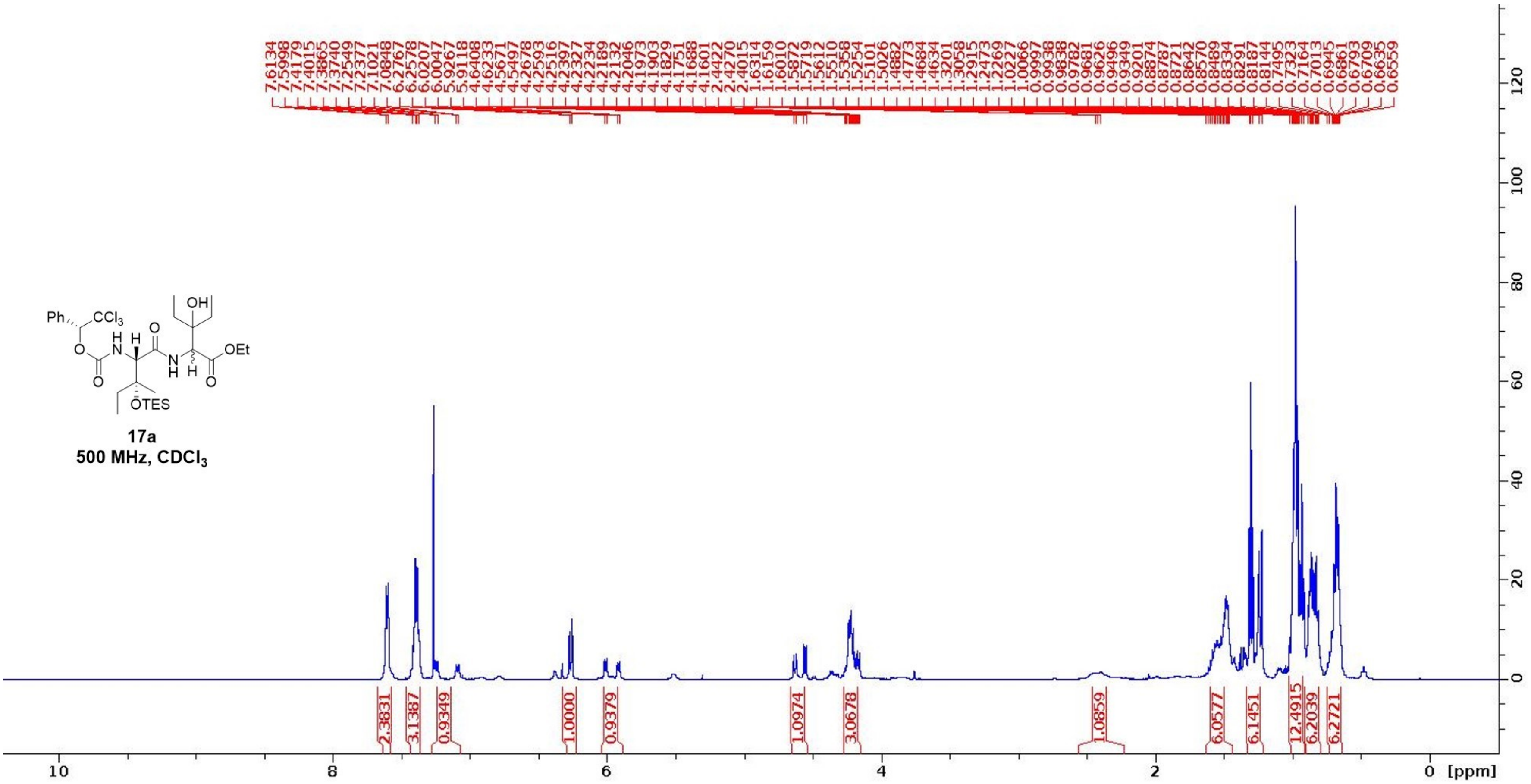

17a
500 MHz, CDCl$_3$

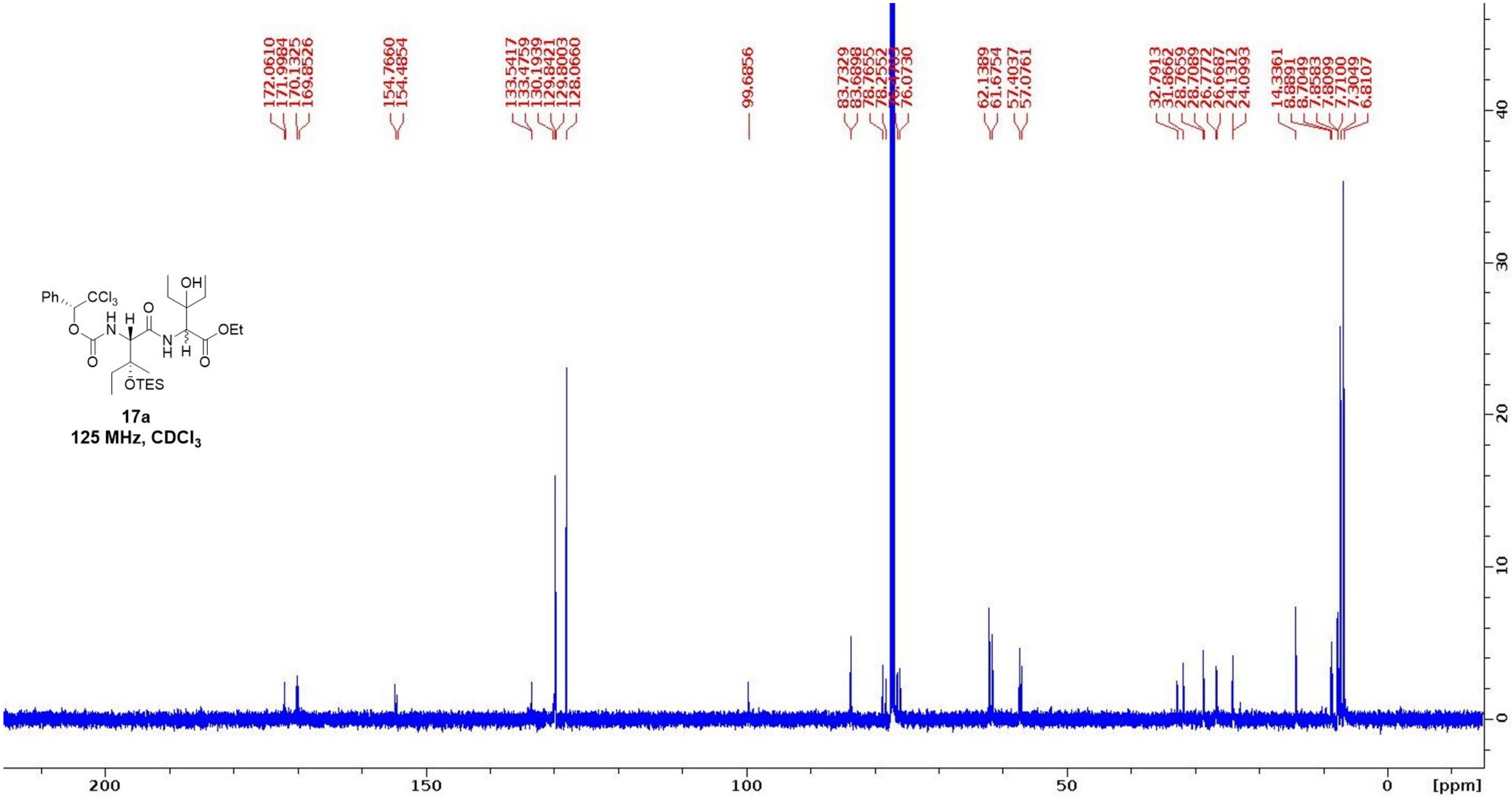

17a
125 MHz, CDCl3
172.0610
171.9984
170.1325
169.8526
154.7660
154.4854
133.5417
133.4759
130.1939
129.8421
129.8003
128.0660
99.6856
83.7329
83.6898
78.7655
78.2552
76.0730
62.1389
61.6754
57.4037
57.0761
32.7913
31.8662
28.7659
28.7069
26.7772
26.6687
24.1312
24.0993
14.3361
8.8891
8.7049
7.8583
7.8099
7.7100
7.3049
6.8107
[ppm]

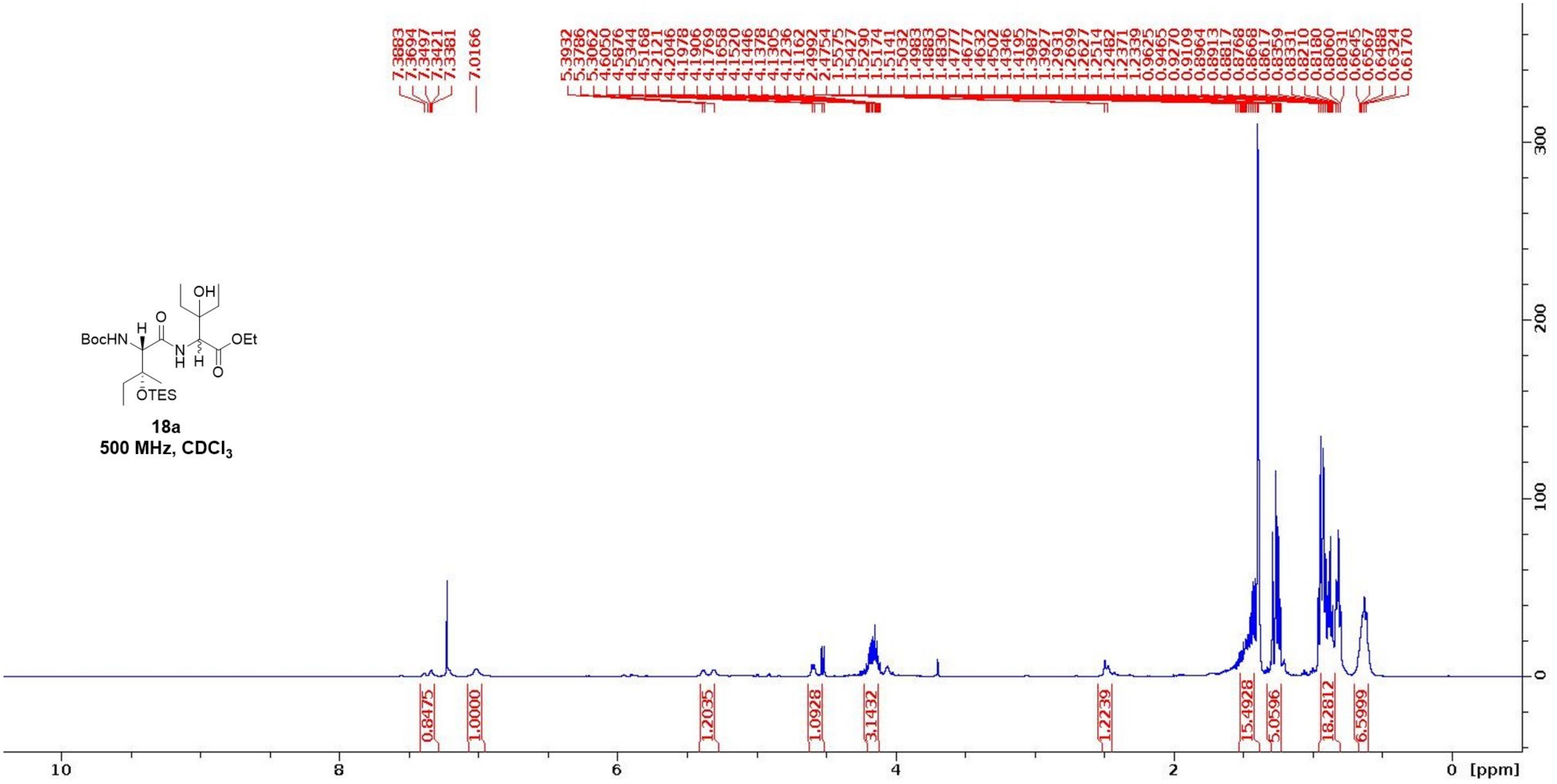
BocHN
OH
OEt
OTES
18a
500 MHz, CDCl3
[ppm]

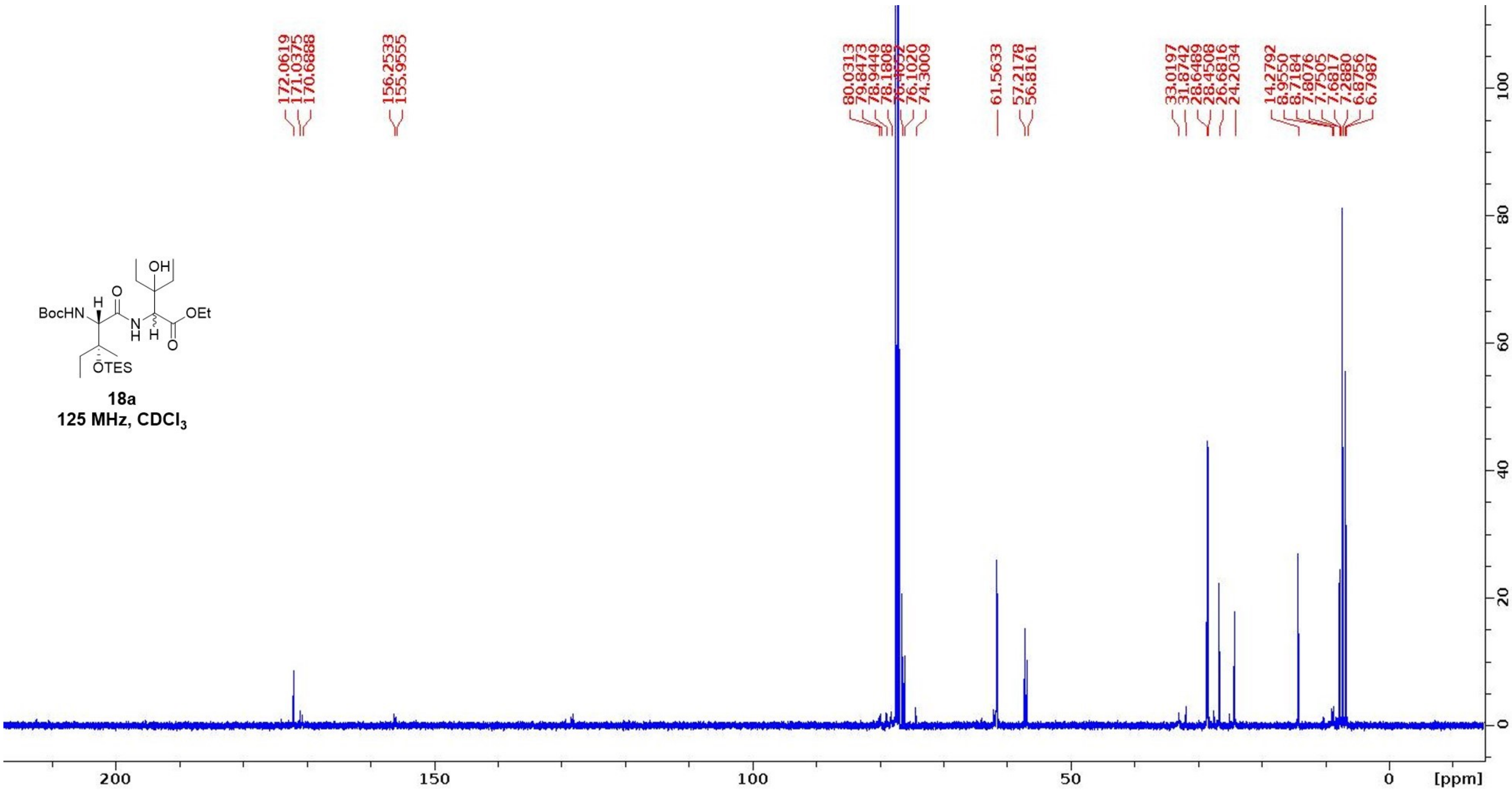

18a
125 MHz, $CDCl_3$

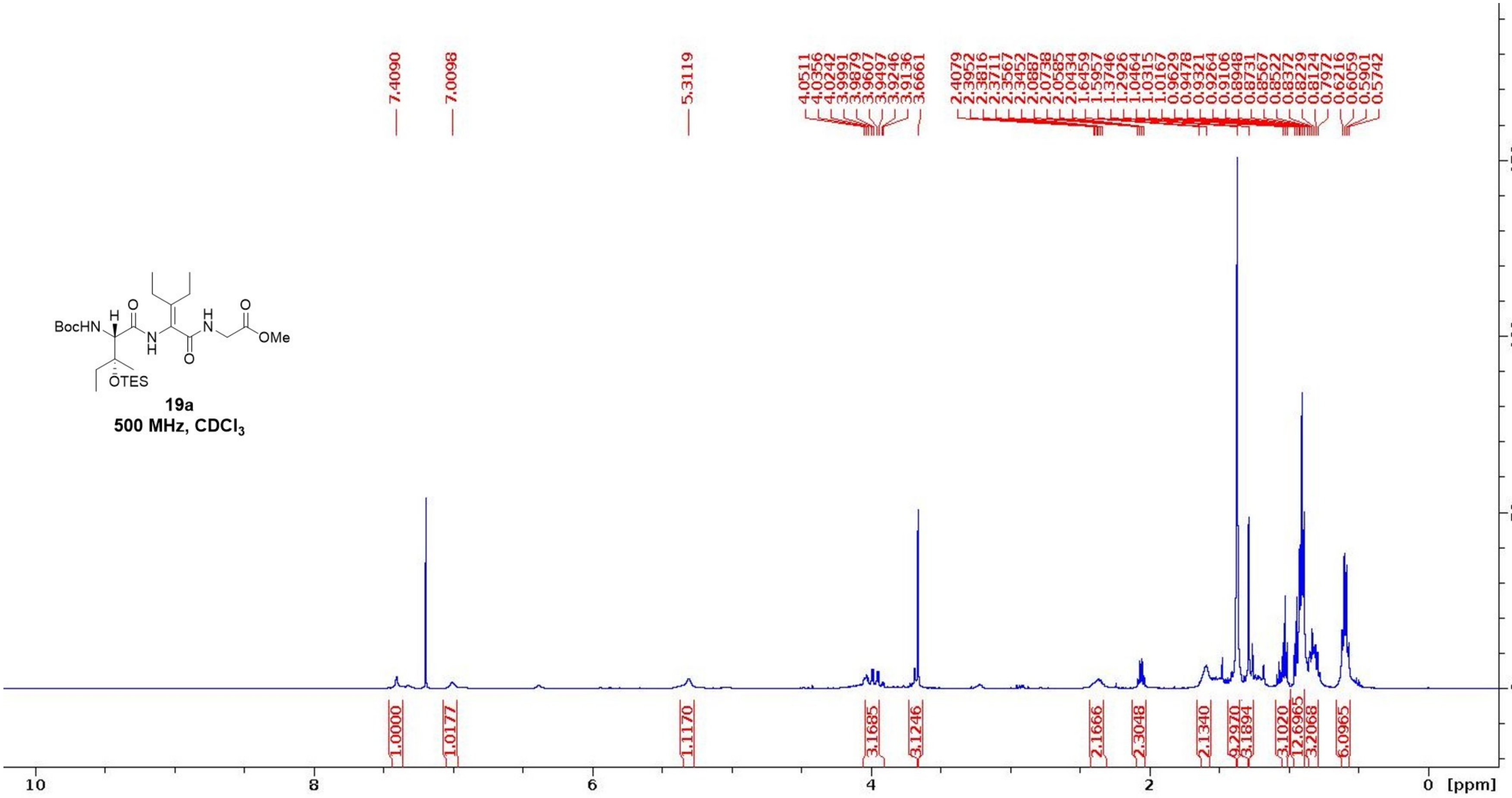
7.4090
7.0098
5.3119
4.0511 4.0356 4.0242 3.9991 3.9879 3.9607 3.9497 3.9246 3.9136 3.6661
2.4079 2.3952 2.3816 2.3711 2.3567 2.3452 2.0887 2.0738 2.0585 2.0434 1.6459 1.5957 1.3746 1.2926 1.0464 1.0315 1.0167 0.9629 0.9478 0.9321 0.9264 0.9106 0.8948 0.8731 0.8567 0.8522 0.8372 0.8229 0.8124 0.7972 0.6216 0.6059 0.5901 0.5742
BocHN
H
O
N
H
O
H
N
O
OMe
OTES
19a
500 MHz, CDCl3
1.0000
1.0177
1.1170
3.1685
3.1246
2.1666
2.3048
2.1340
9.2970
3.1894
3.1020
12.6965
3.2068
6.0965
10
8
6
4
2
0 [ppm]
150
100
50
0

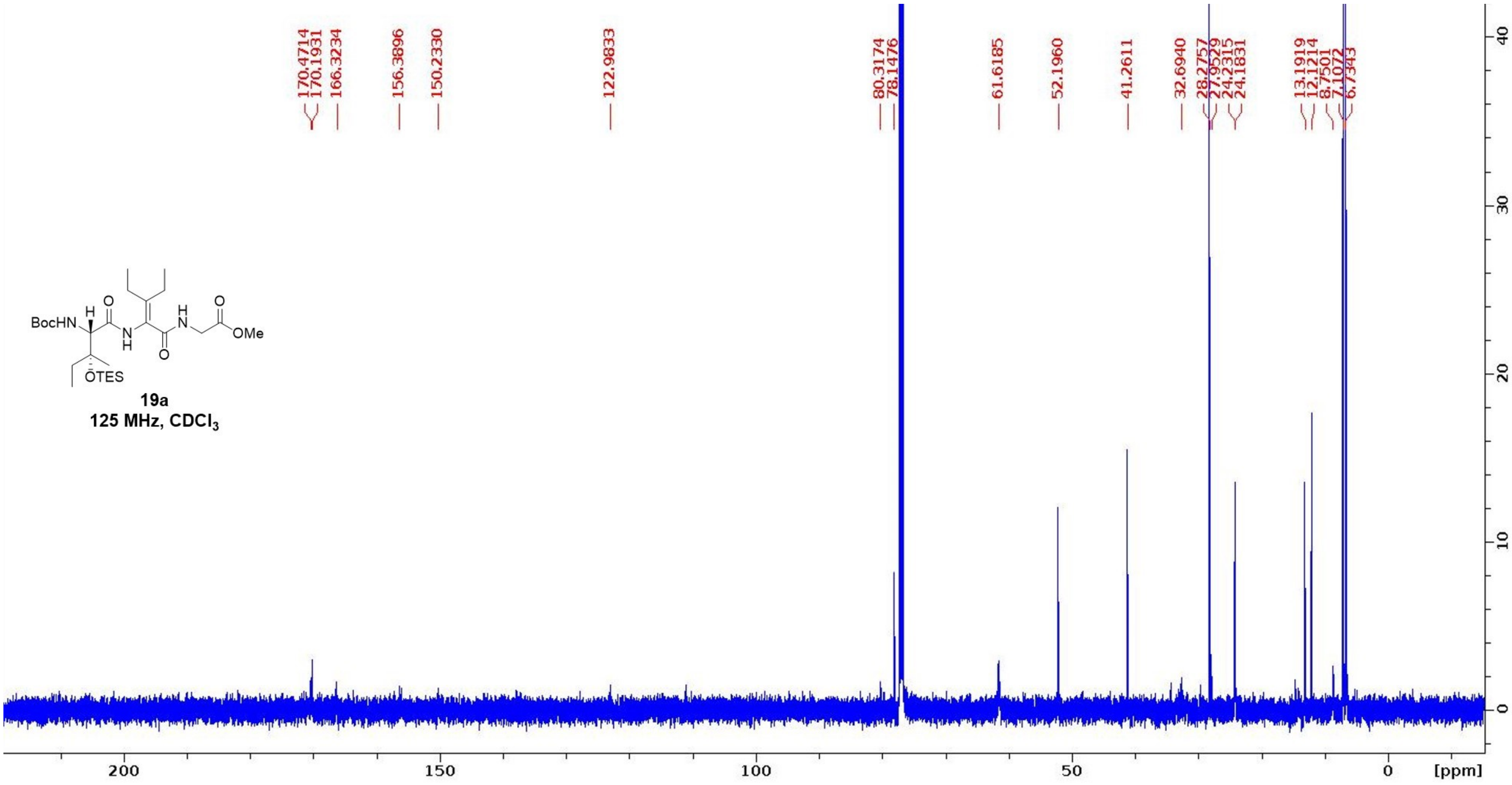

19a
125 MHz, $CDCl_3$

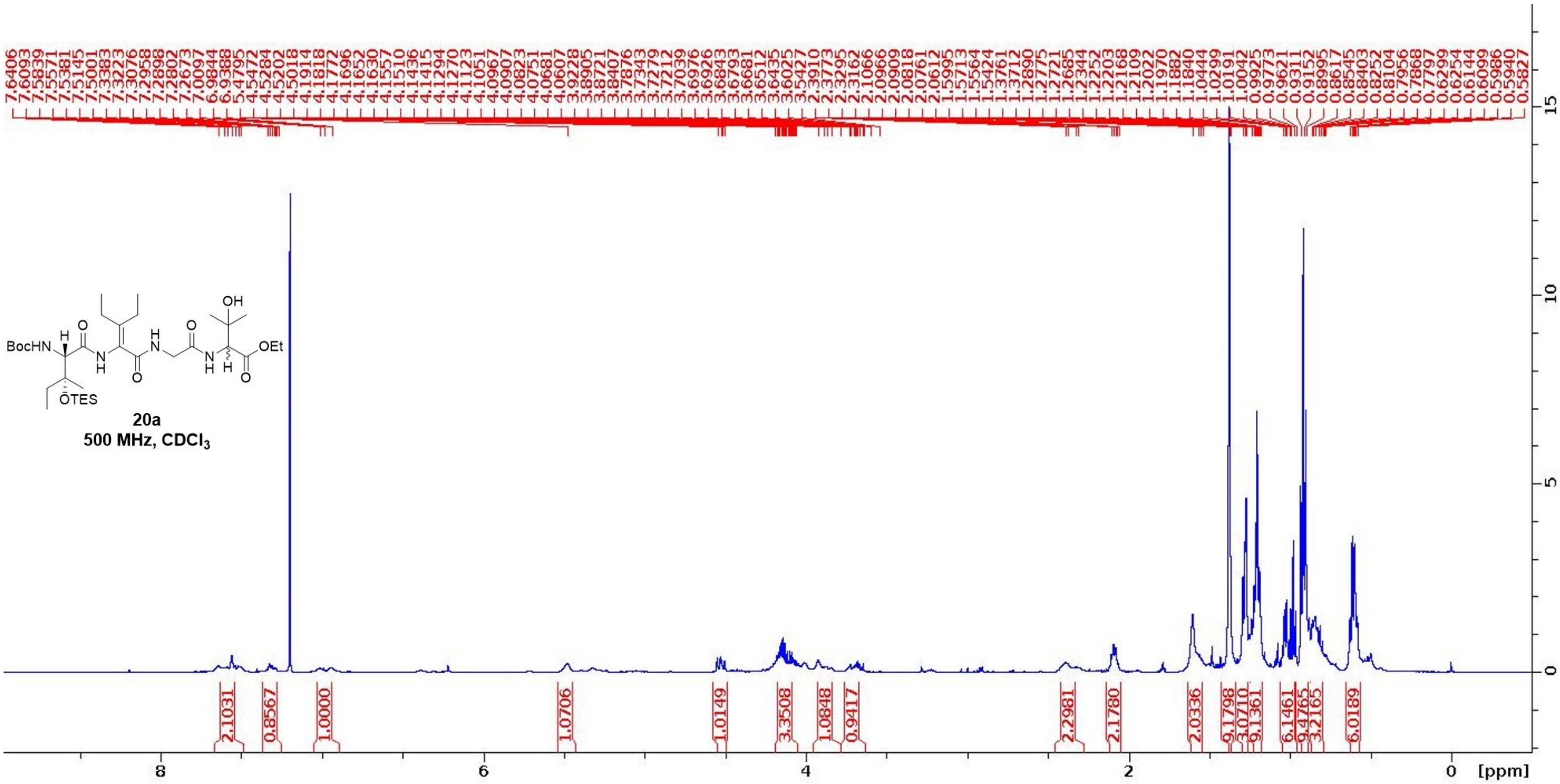

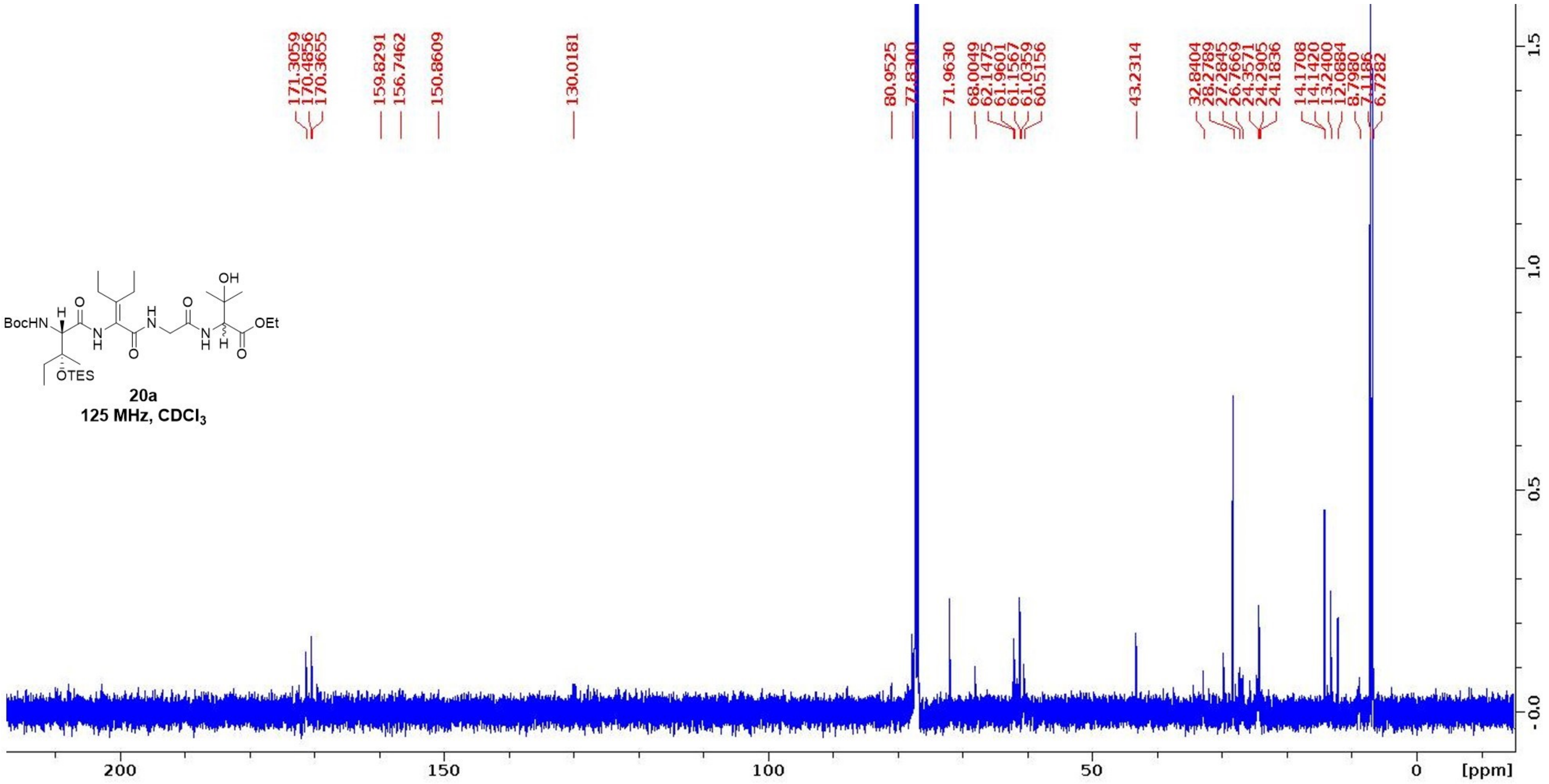

20a
125 MHz, $CDCl_3$

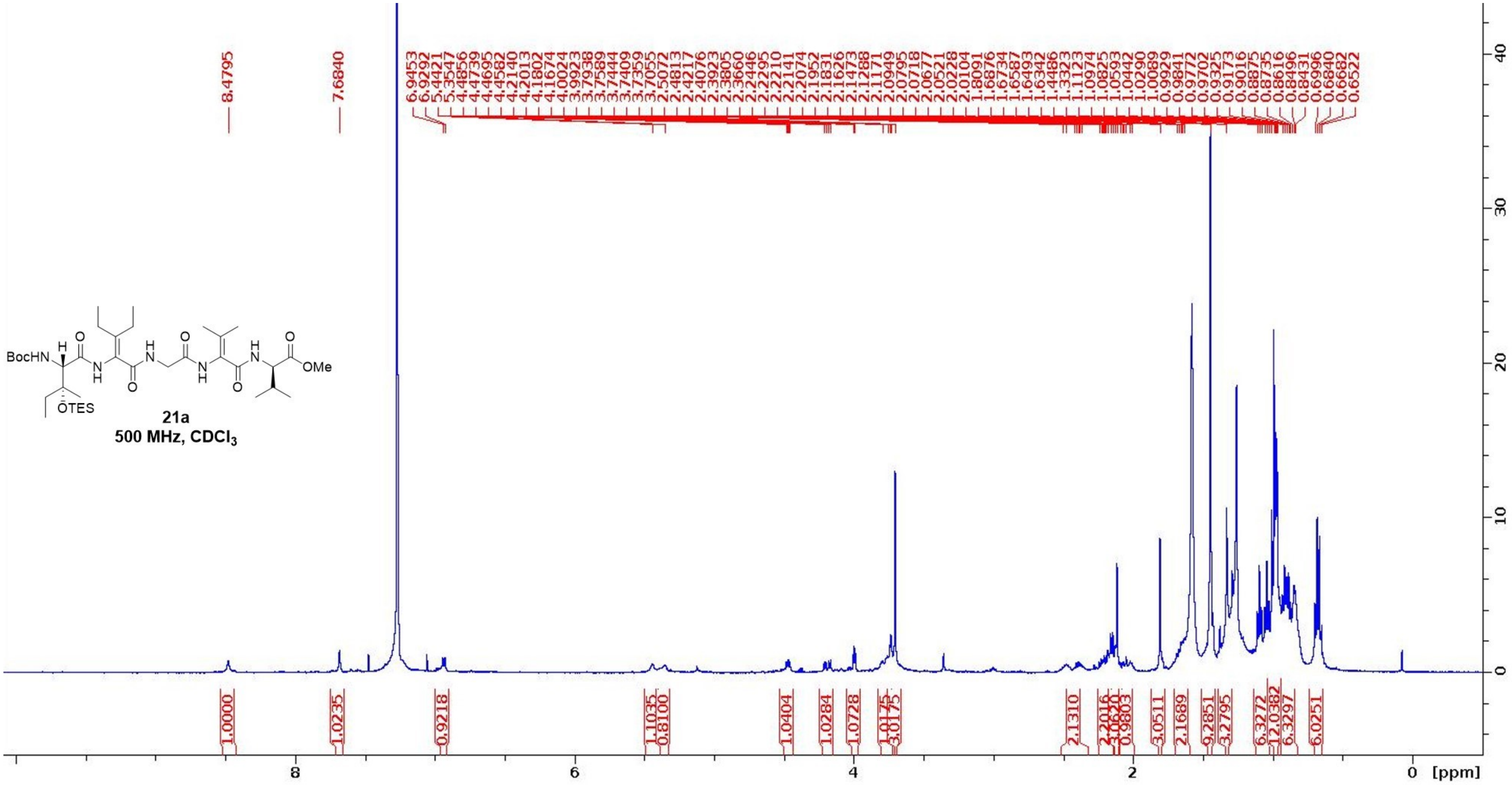
BocHN
OTES
OMe
21a
500 MHz, CDCl3
[ppm]

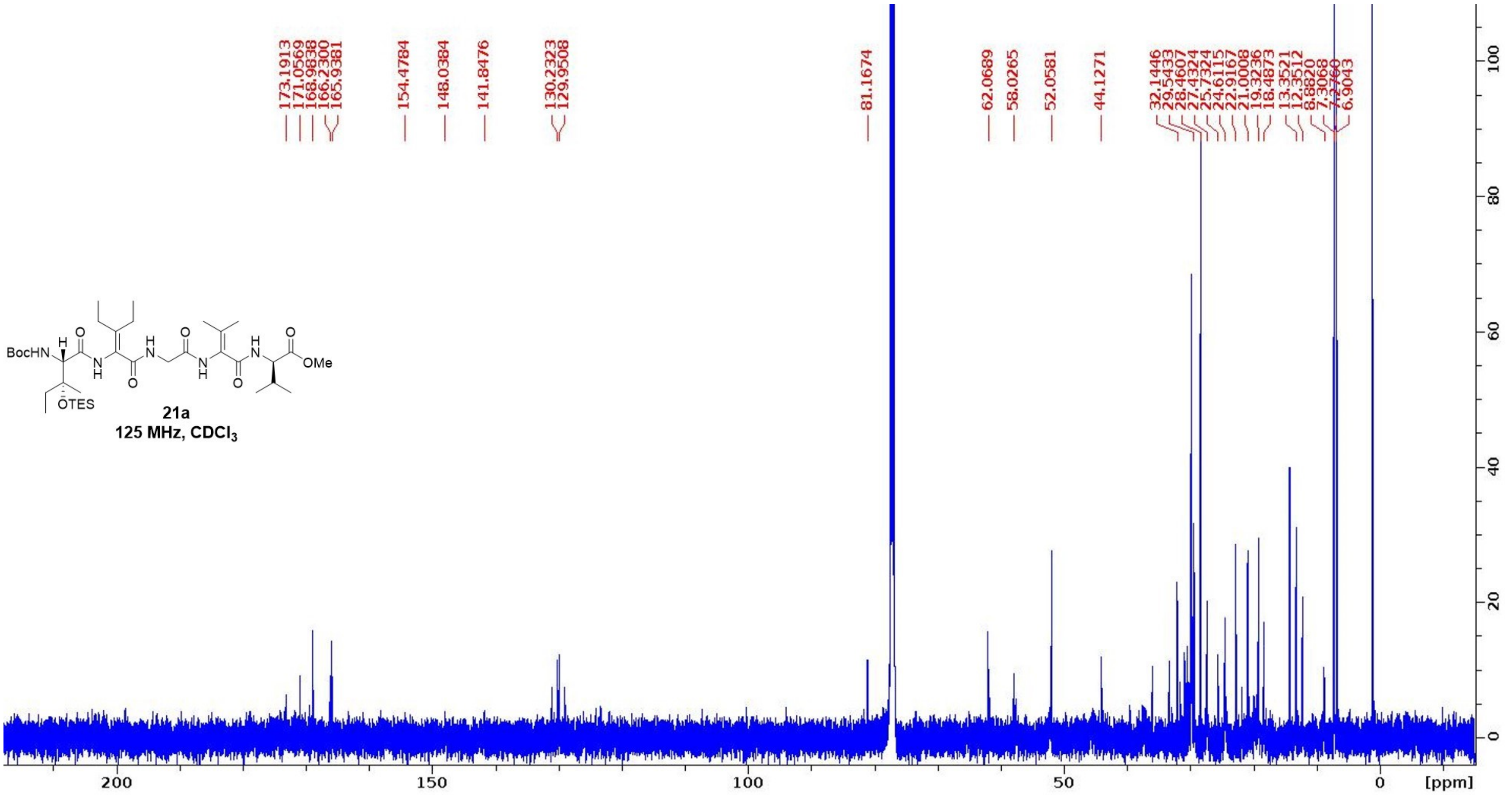
173.1913
171.0569
168.9838
166.2300
165.9381
154.4784
148.0384
141.8476
130.2323
129.9508
81.1674
62.0689
58.0265
52.0581
44.1271
32.1446
29.5433
28.4607
27.4324
25.7324
24.6115
22.9167
21.0008
19.3236
18.4873
13.3521
12.3512
8.8820
7.3068
7.2763
6.9043
BocHN
OTES
OMe
21a
125 MHz, CDCl3
[ppm]

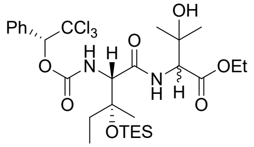

17b
500 MHz, $CDCl_3$

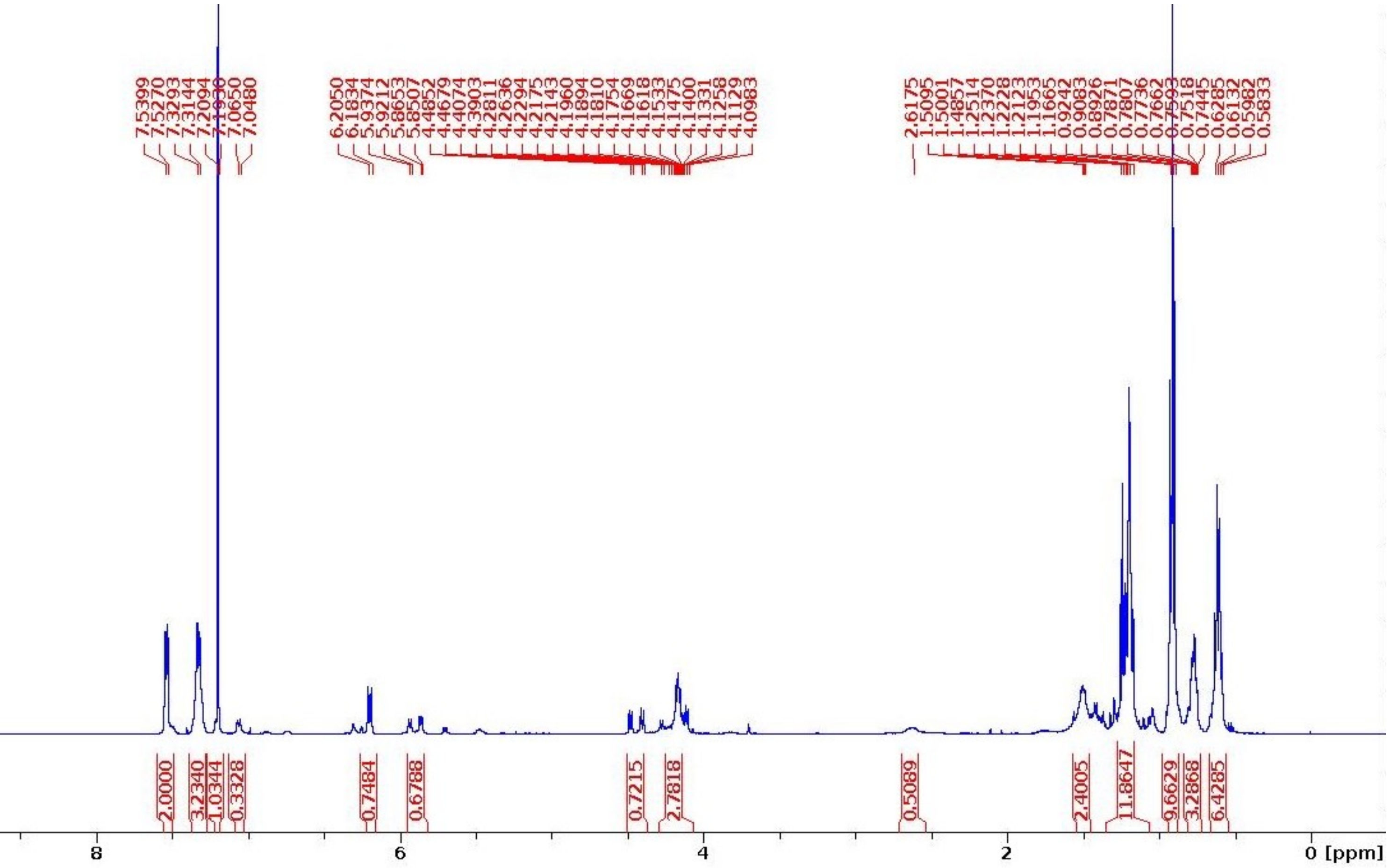

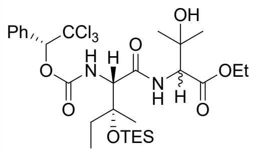

17b
125 MHz, $CDCl_3$

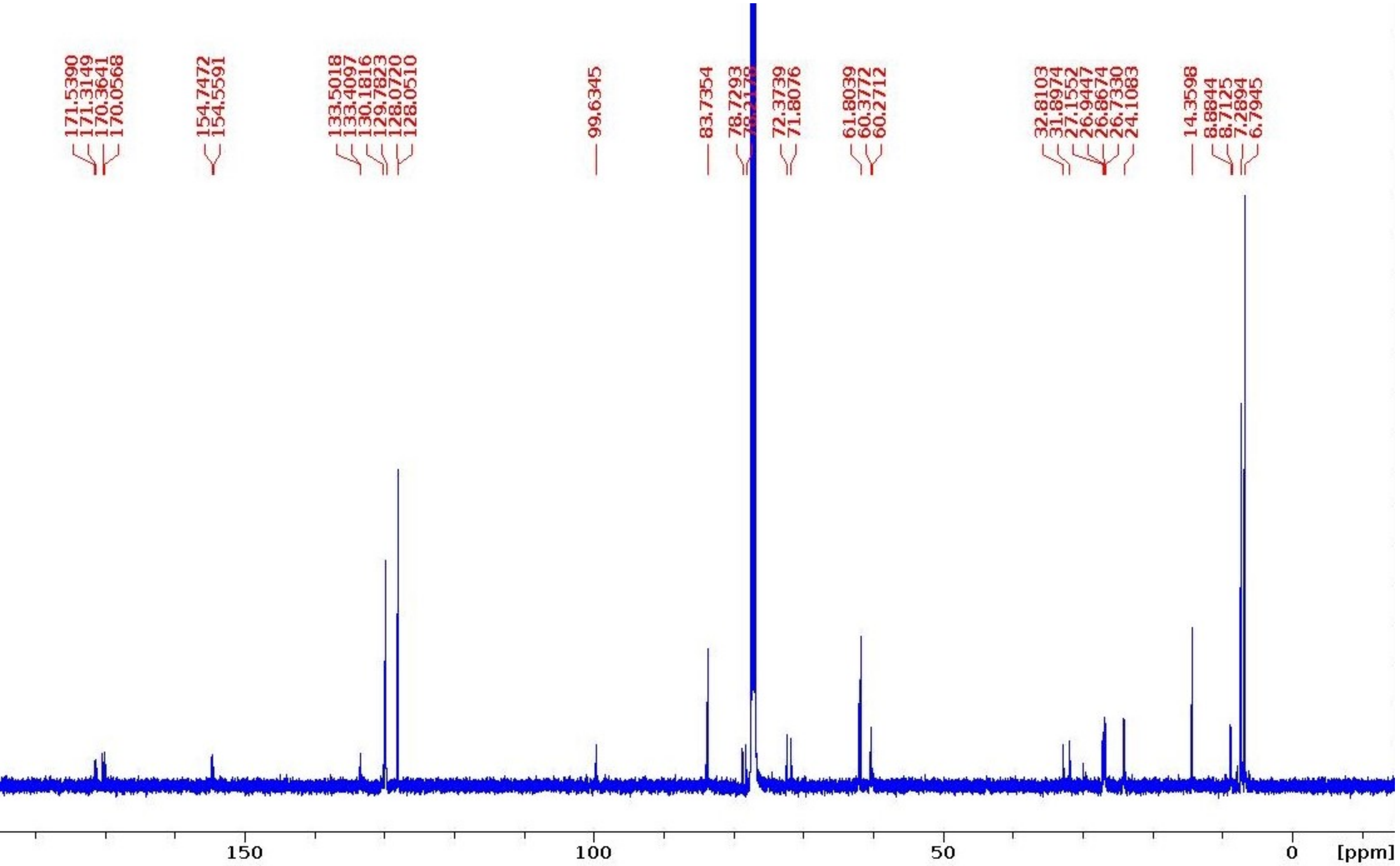

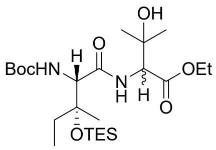

18b

500 MHz, $CDCl_3$

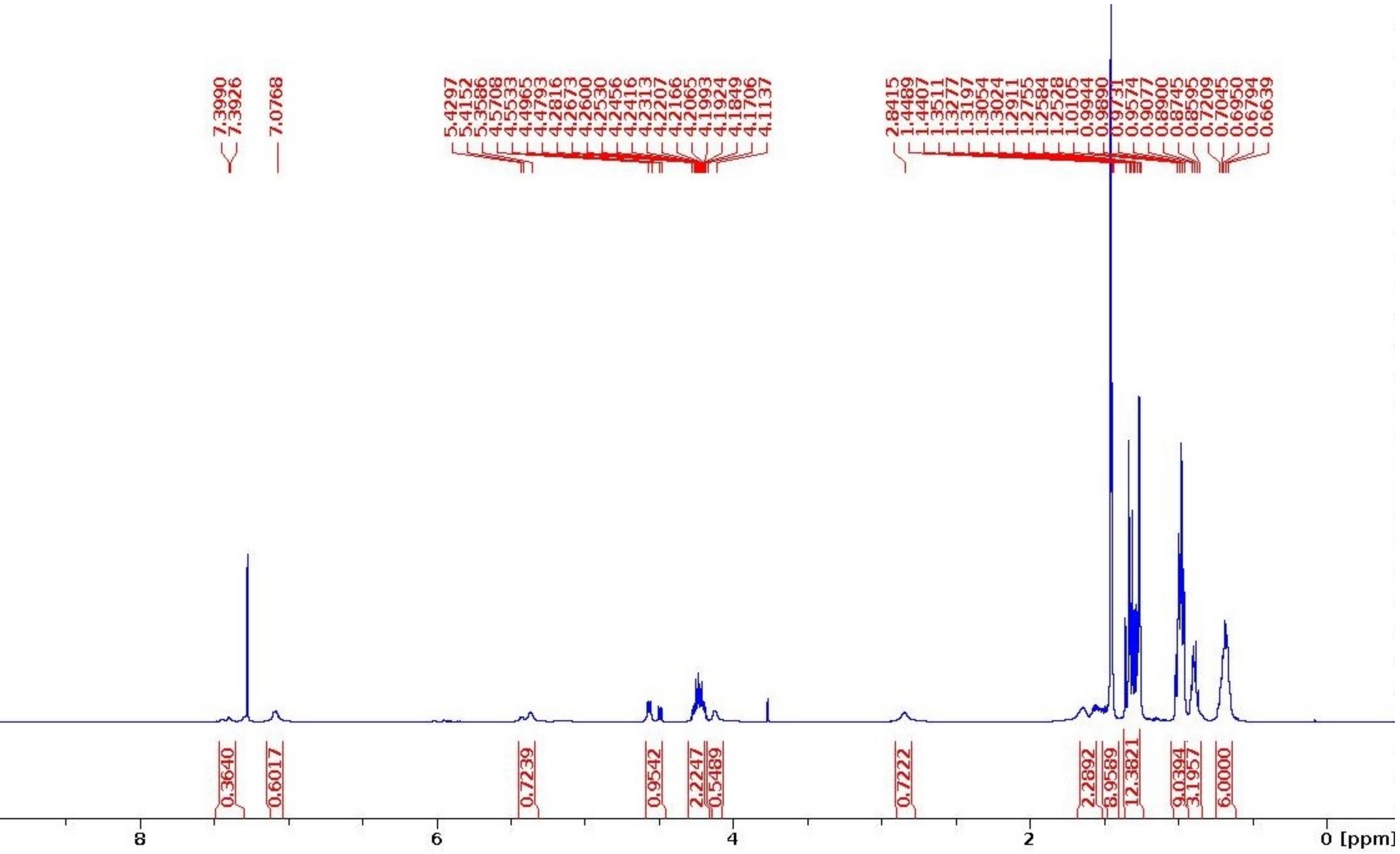

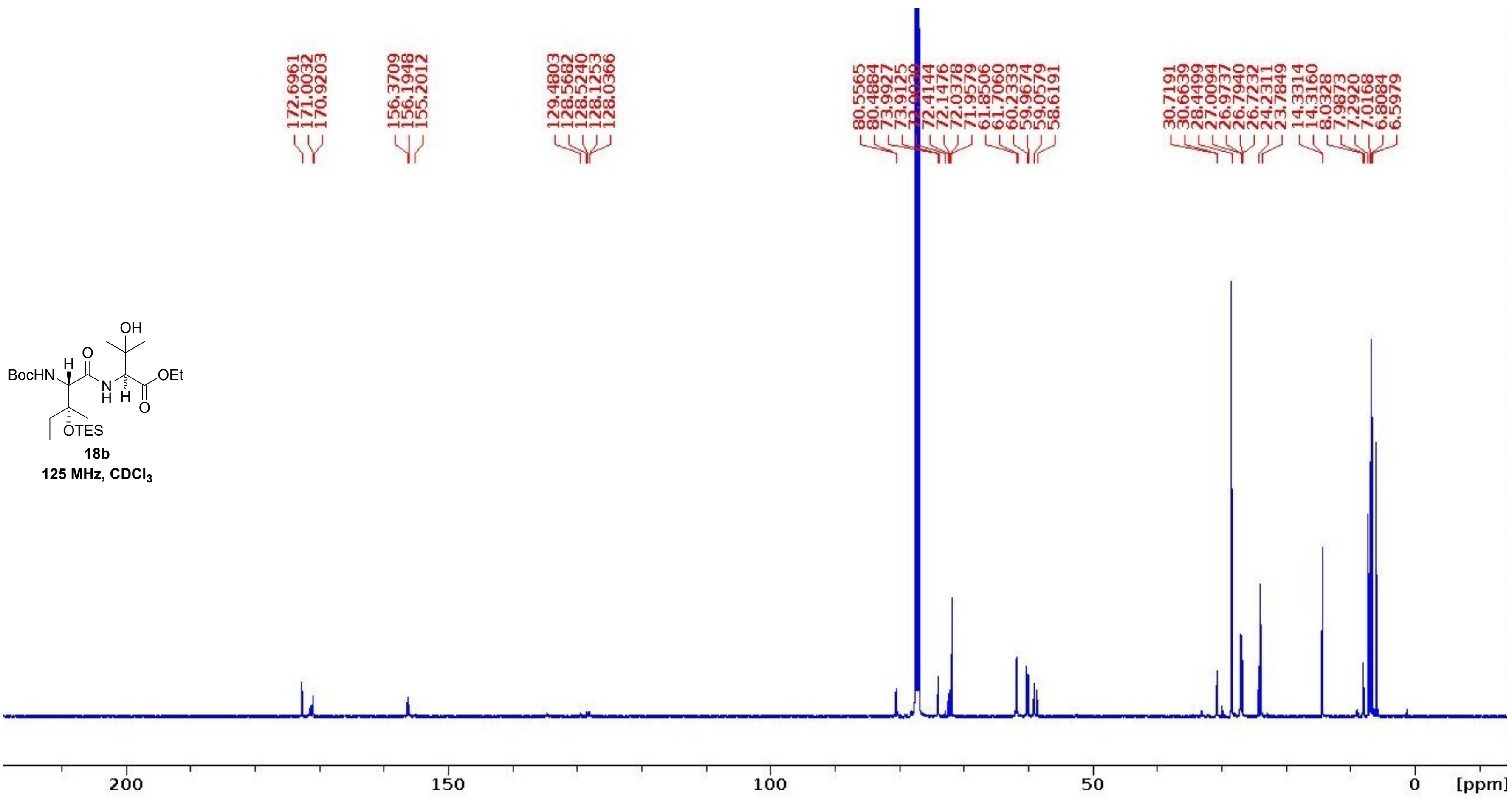

18b
125 MHz, $CDCl_3$

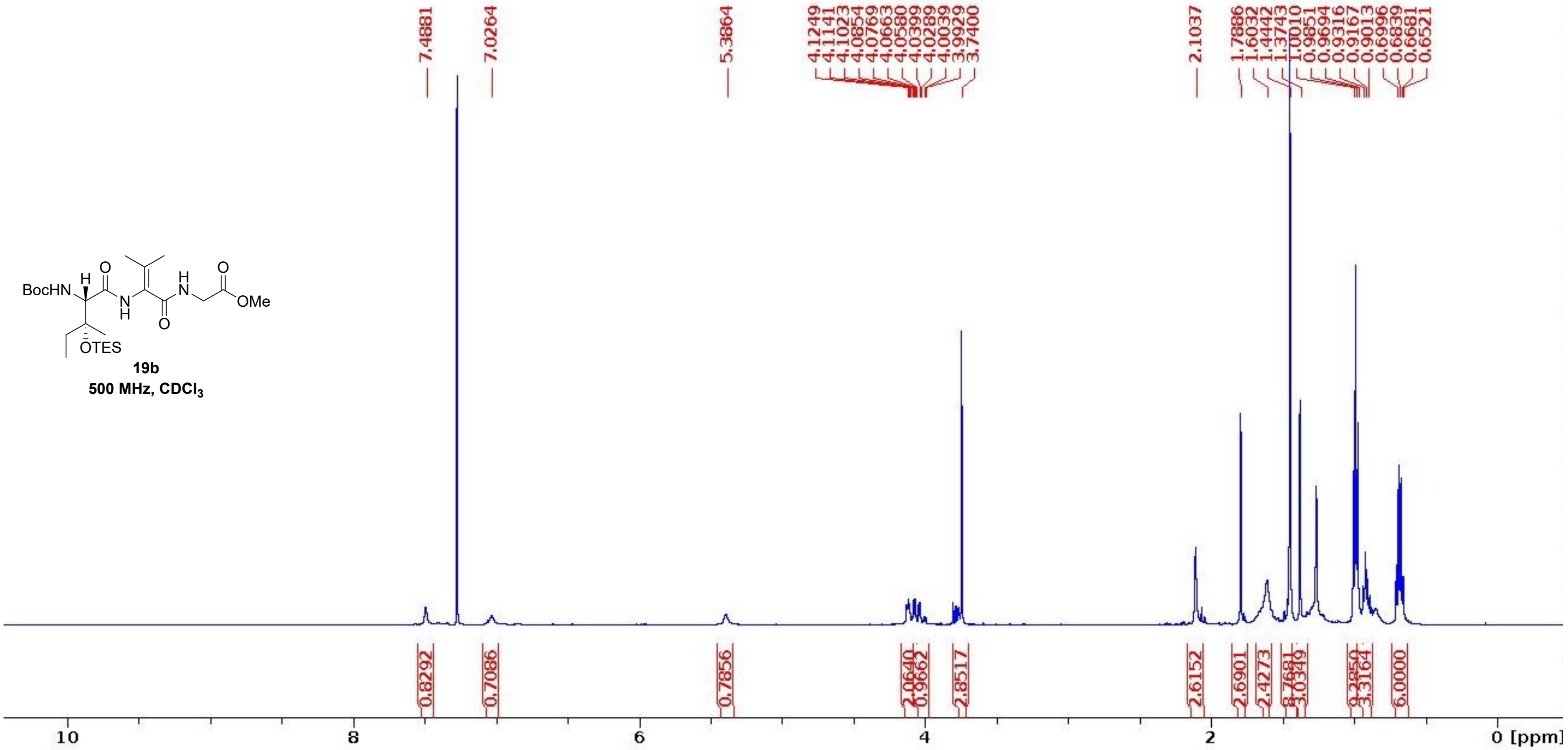
BocHN
H
O
N
H
O
H
N
O
OMe
OTES
19b
500 MHz, CDCl3
7.4881
7.0264
5.3864
4.1249
4.1141
4.1023
4.0854
4.0769
4.0663
4.0580
4.0399
4.0289
4.0039
3.9929
3.7400
2.1037
1.7886
1.6032
1.4442
1.3743
1.0010
0.9851
0.9694
0.9316
0.9167
0.9013
0.6996
0.6839
0.6681
0.6521
0.8292
0.7086
0.7856
2.8517
2.6152
2.6901
2.4273
6.0000
10
8
6
4
2
0 [ppm]

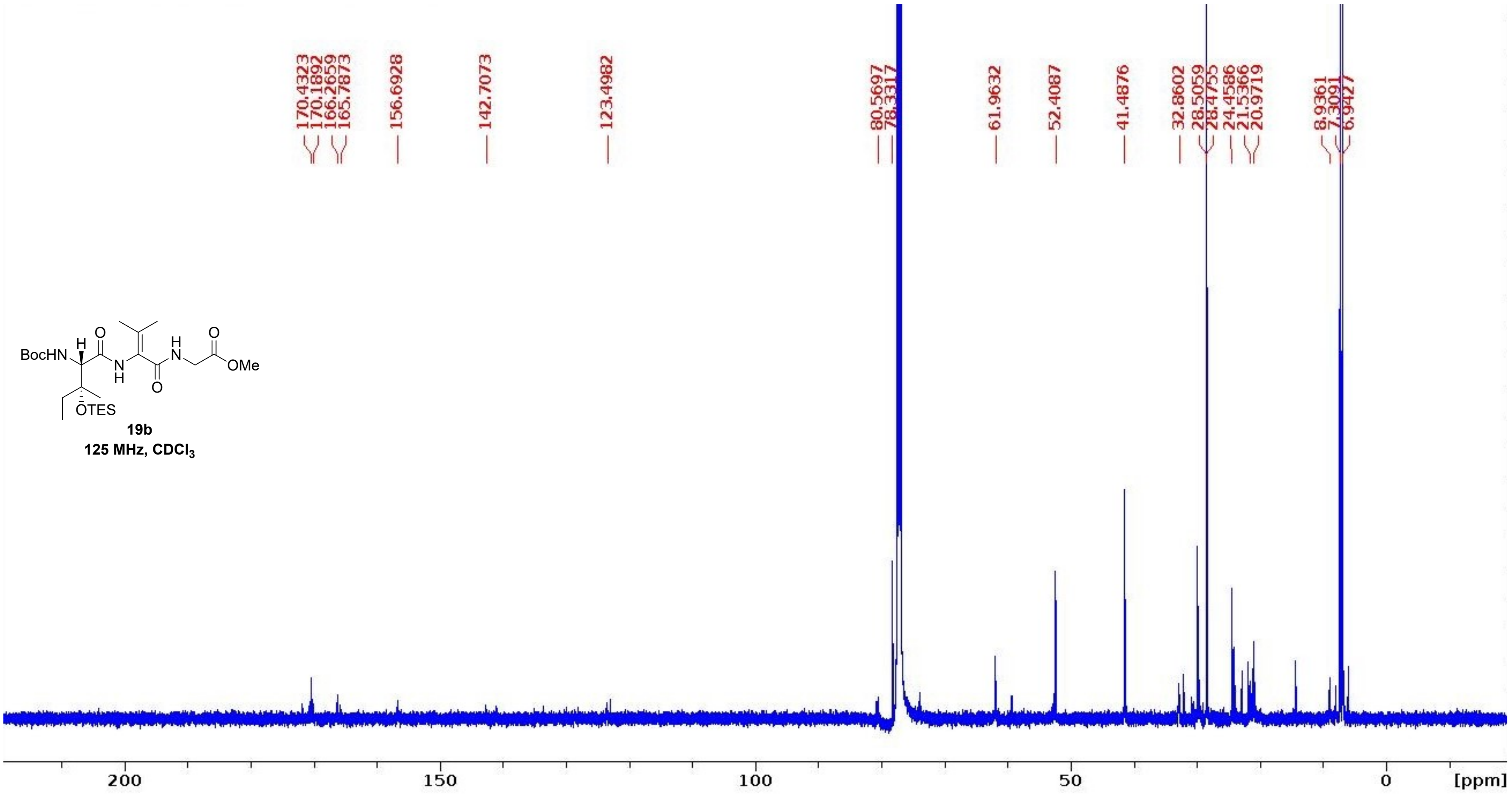

170.4323
170.1892
166.2659
165.7873
156.6928
142.7073
123.4982
80.5697
78.3317
61.9632
52.4087
41.4876
32.8602
28.5059
28.4755
24.4586
21.5366
20.9719
8.9361
7.3091
6.9427
BocHN
OTES
OMe
19b
125 MHz, CDCl3
200
150
100
50
0
[ppm]

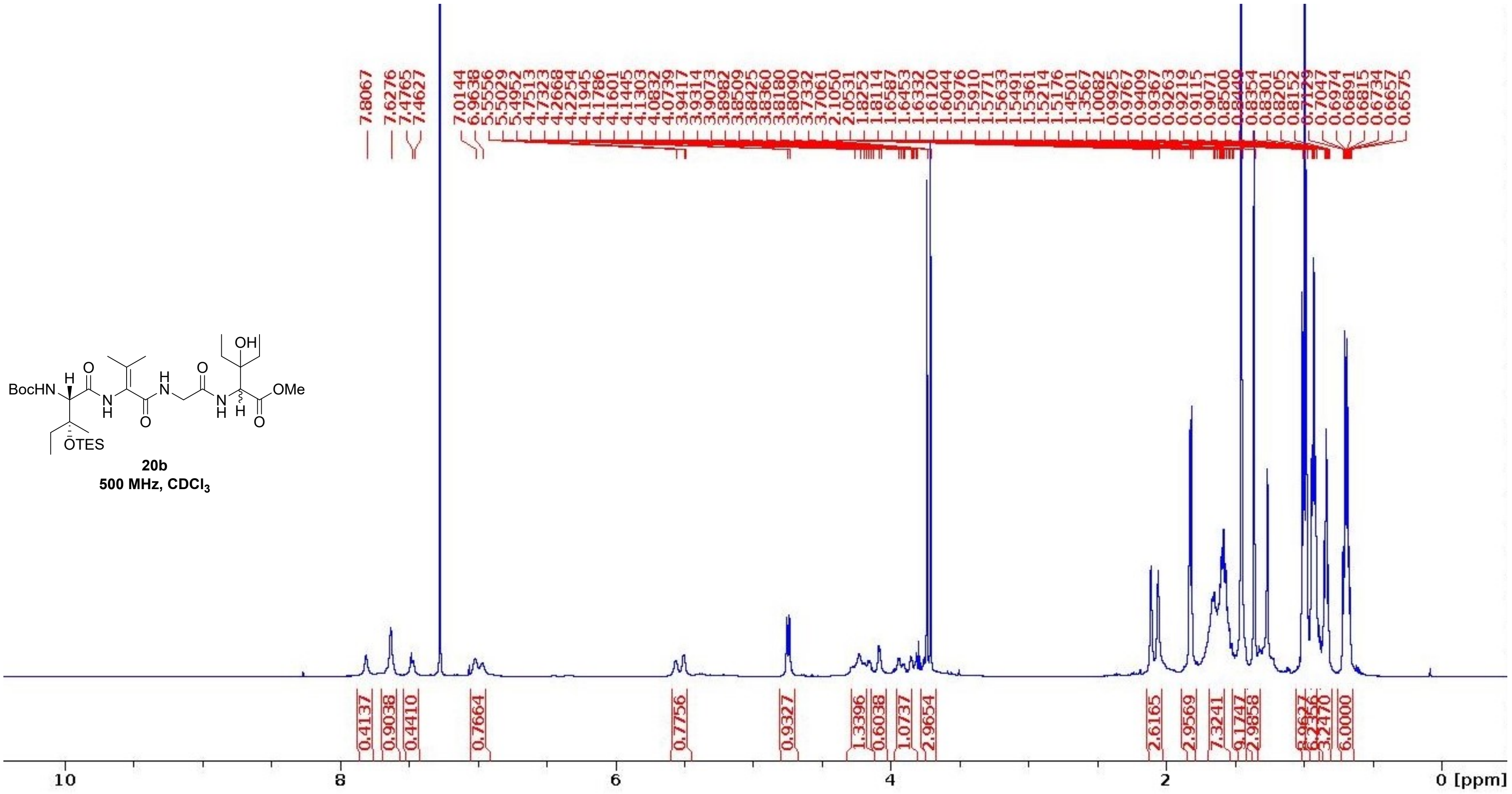

20b
500 MHz, $CDCl_3$

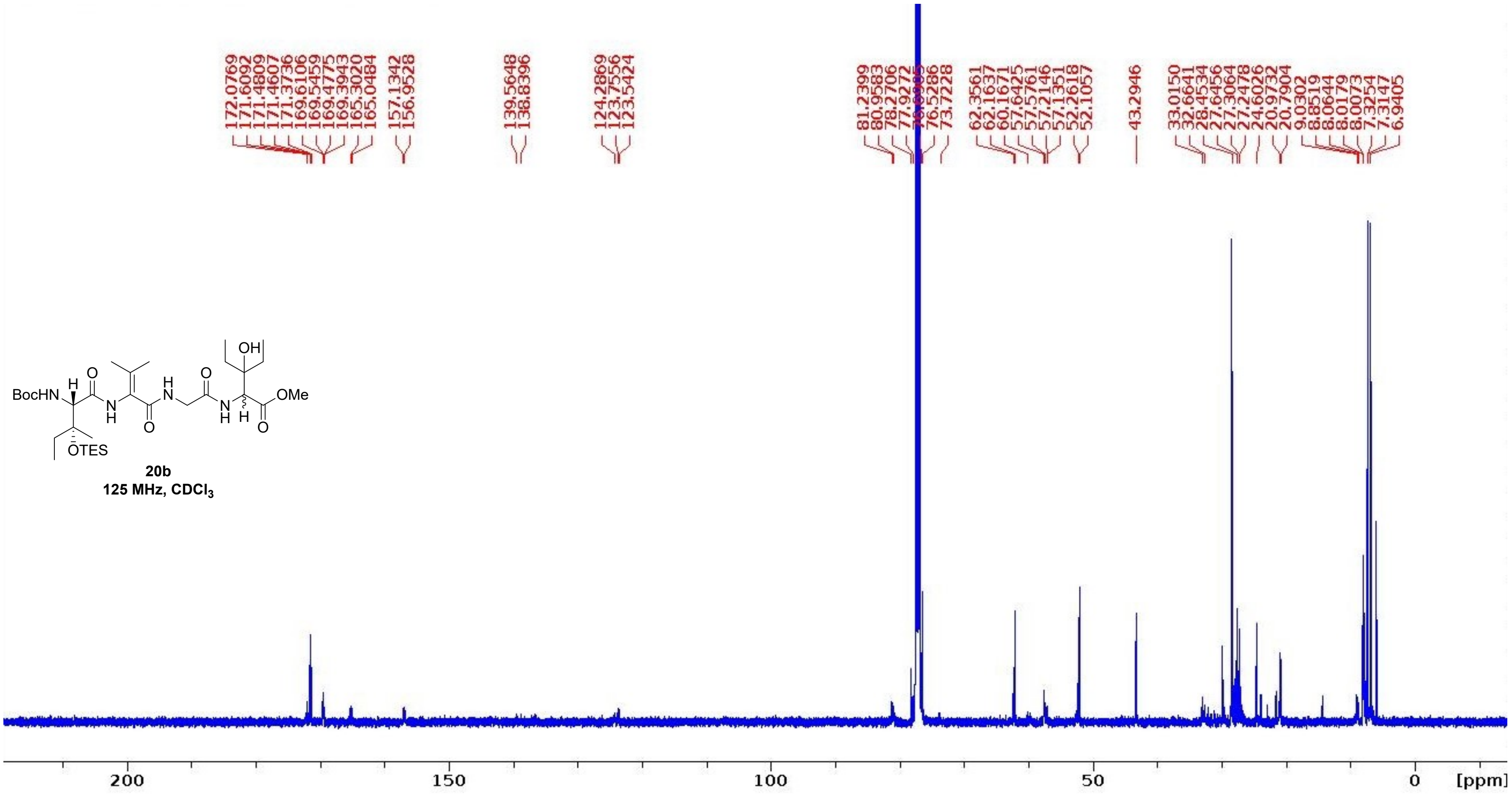

20b
125 MHz, $CDCl_3$

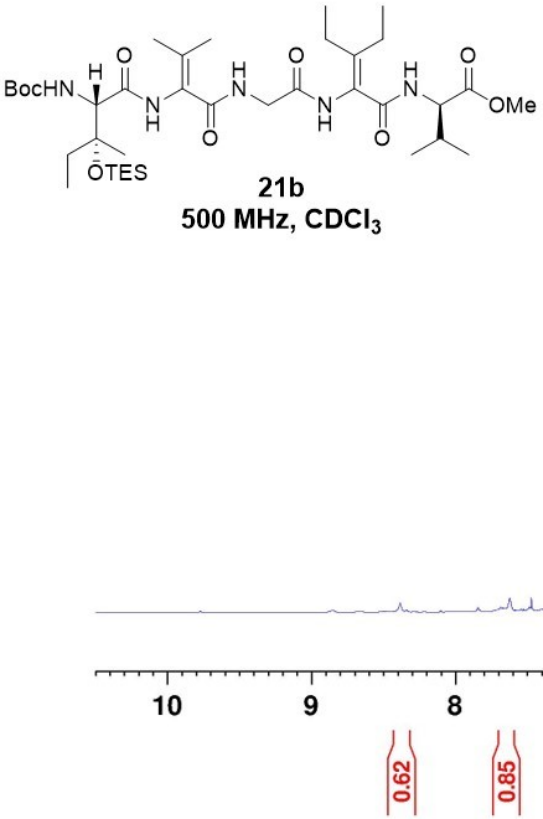

21b
500 MHz, $CDCl_3$

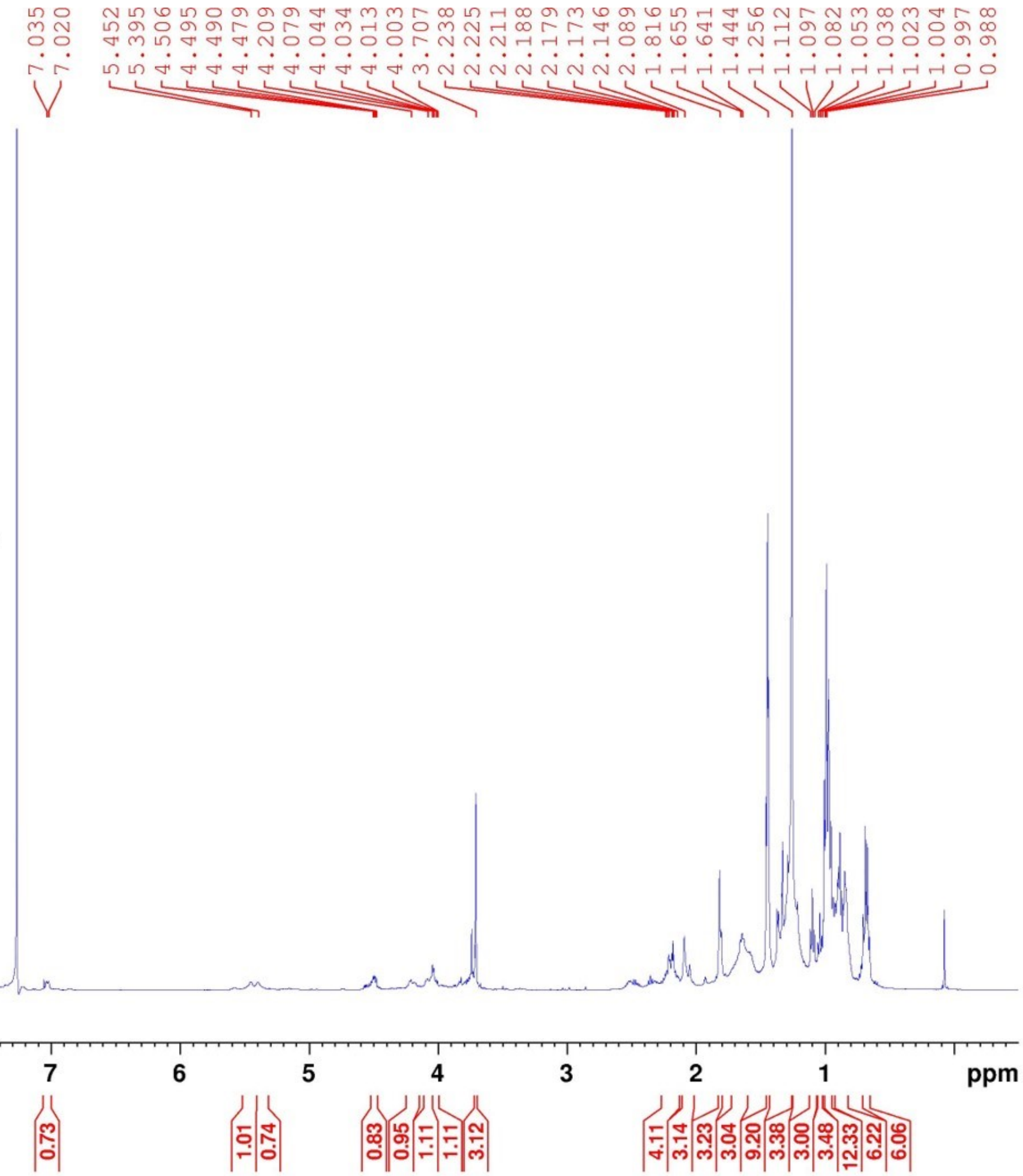

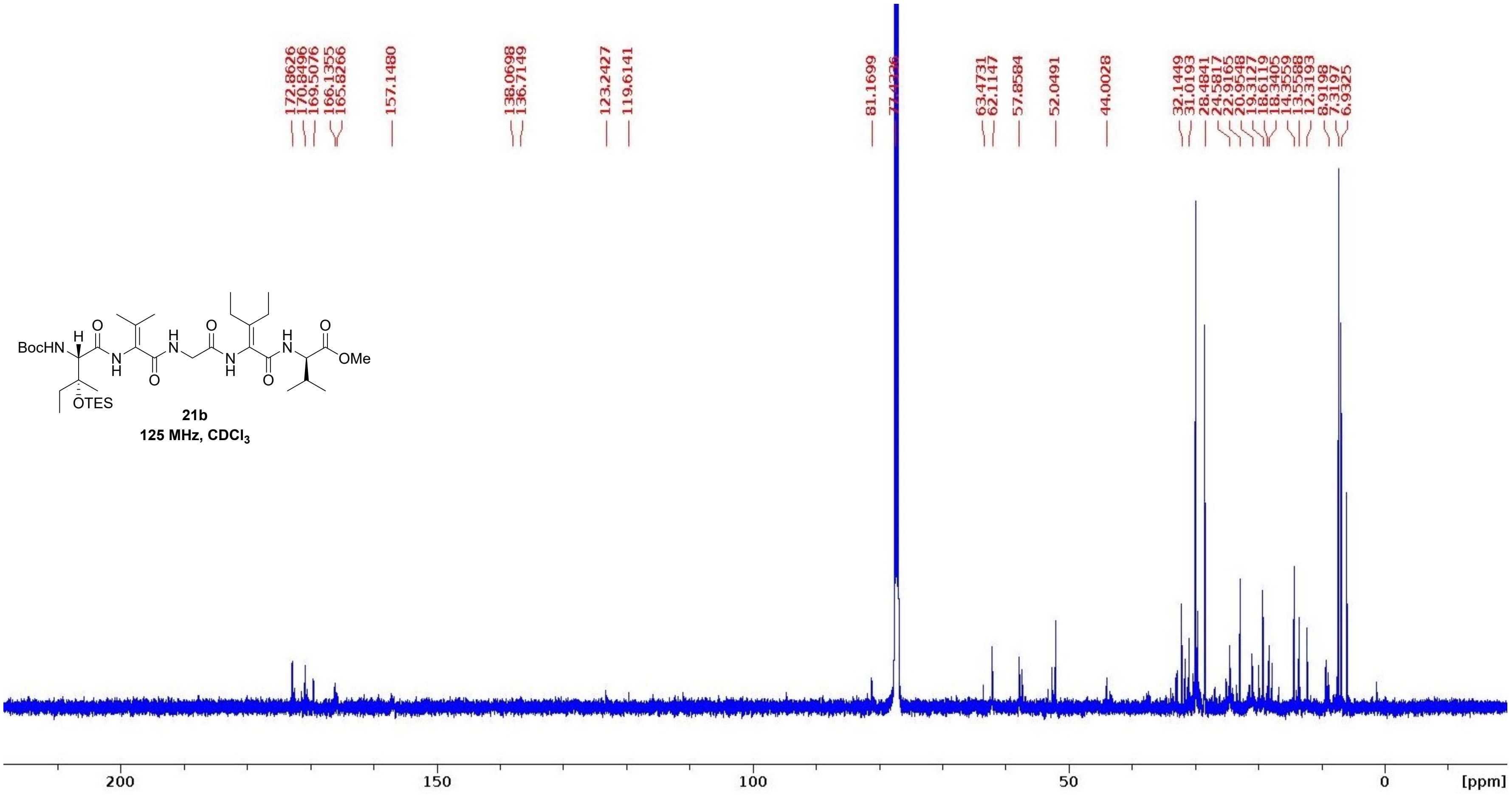
172.8626
170.8496
169.5076
166.1355
165.8266
157.1480
138.0698
136.7149
123.2427
119.6141
81.1699
63.4731
62.1147
57.8584
52.0491
44.0028
32.1449
31.0193
28.4841
24.5817
22.9165
20.9548
19.3127
18.6119
18.3405
14.3559
13.5588
12.3193
8.9198
7.3197
6.9325
BocHN
OTES
OMe
21b
125 MHz, $CDCl_3$
200
150
100
50
0
[ppm]

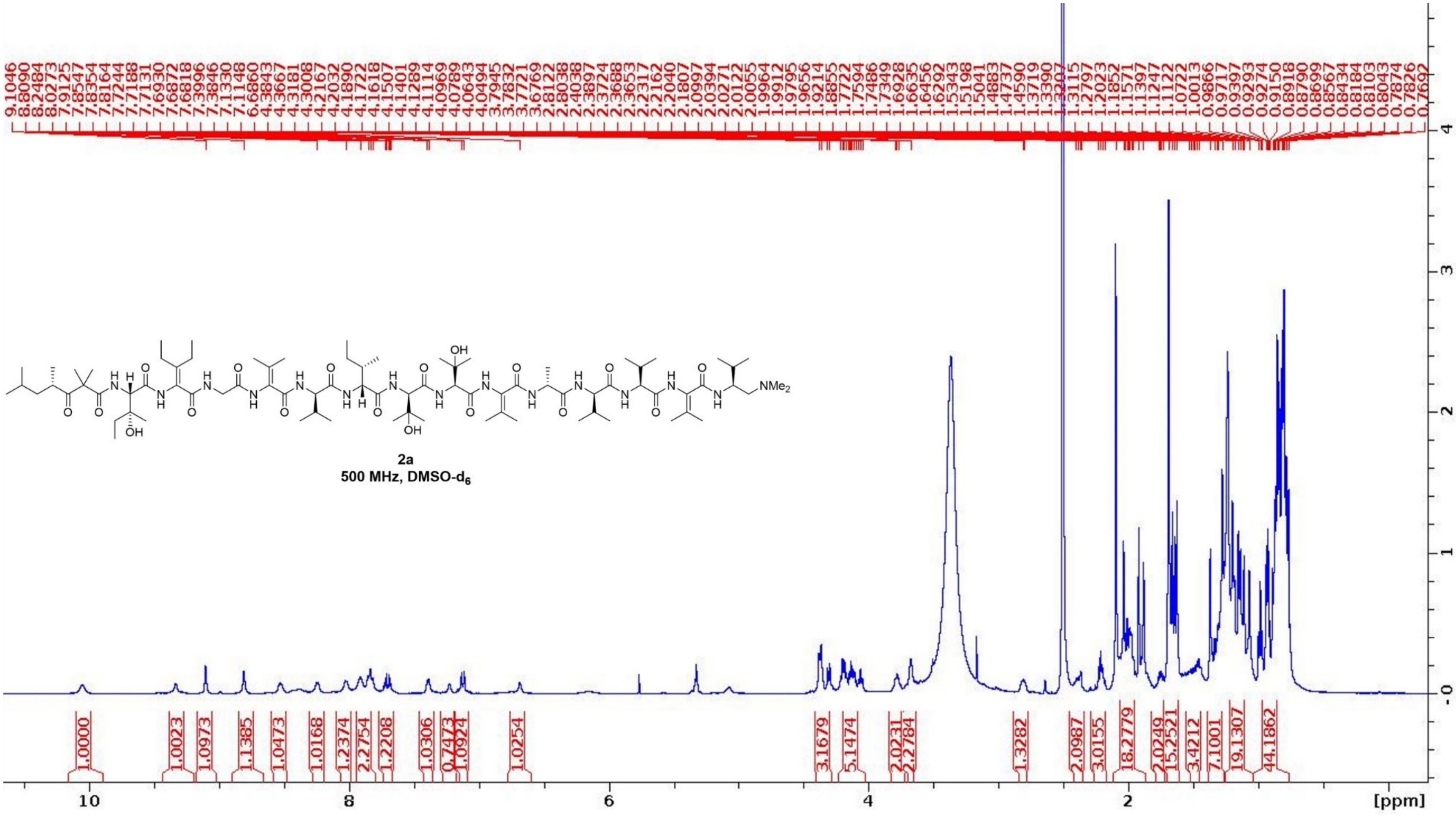
2a
500 MHz, DMSO-d6
[ppm]

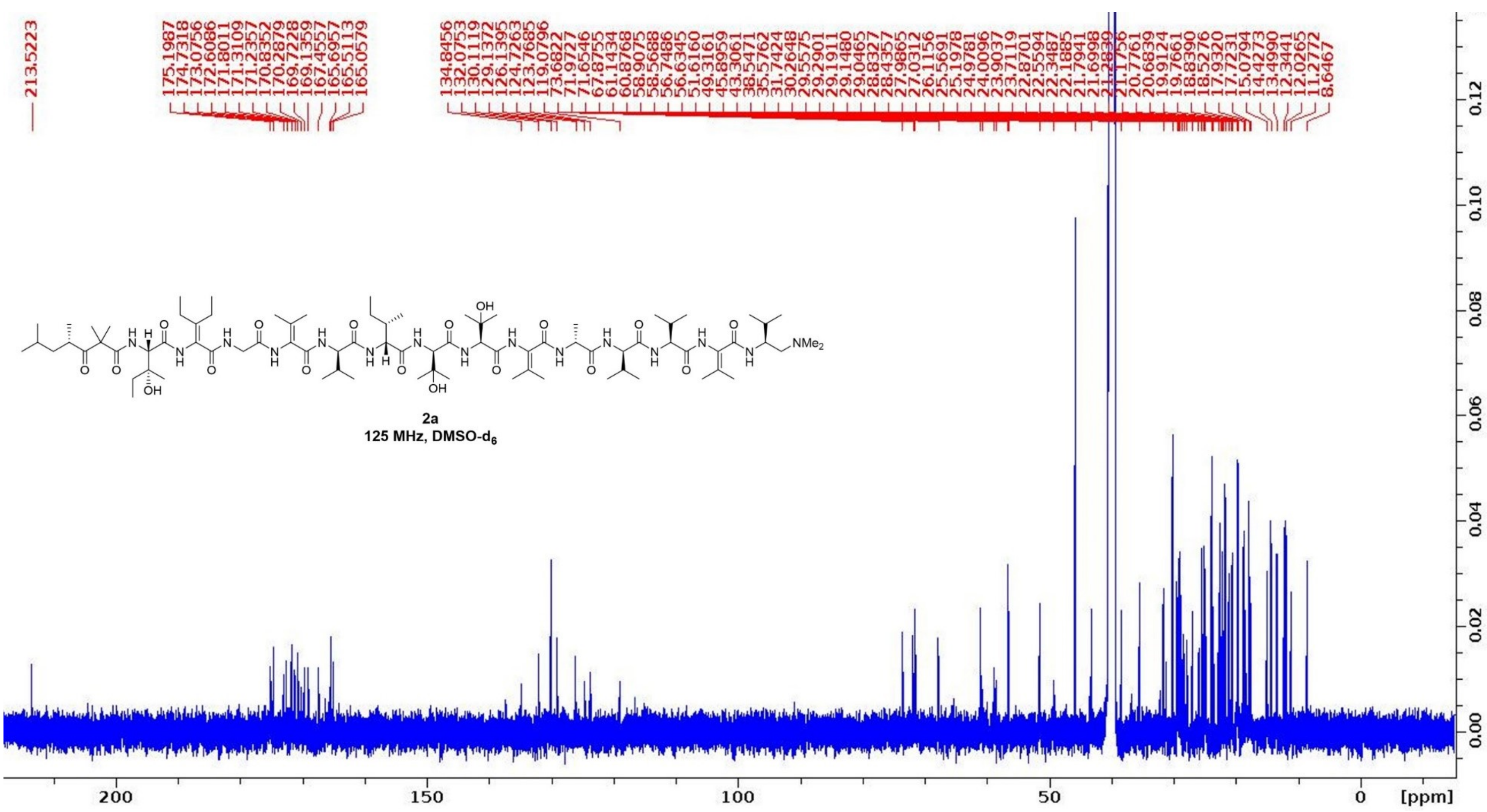
213.5223
175.1987
174.7318
173.0756
172.6086
171.8011
171.3109
171.2357
170.8352
170.2879
169.7228
169.1359
167.4557
165.6957
165.5113
165.0579
134.8456
132.0753
130.1119
129.1372
126.1395
124.7263
123.7685
119.0796
73.6822
71.9727
71.6546
67.8755
61.1434
60.8768
58.9075
58.5688
56.7486
56.6345
51.6160
49.3161
45.8959
43.3061
38.5471
35.5762
31.7424
30.2648
29.5575
29.2901
29.1911
29.1480
29.0465
28.8327
28.4357
27.9865
27.0312
26.1156
25.5691
25.1978
24.9781
24.0096
23.9037
23.7119
22.8701
22.5594
22.3487
22.1885
21.7941
21.6998
20.7761
20.6639
19.9124
19.7661
18.8390
18.5276
17.9320
17.7231
15.0794
14.4273
13.4990
12.3441
12.0265
11.2772
8.6467
OH
NMe2
2a
125 MHz, DMSO-d6
200
150
100
50
0
[ppm]
0.12
0.10
0.08
0.06
0.04
0.02
0.00

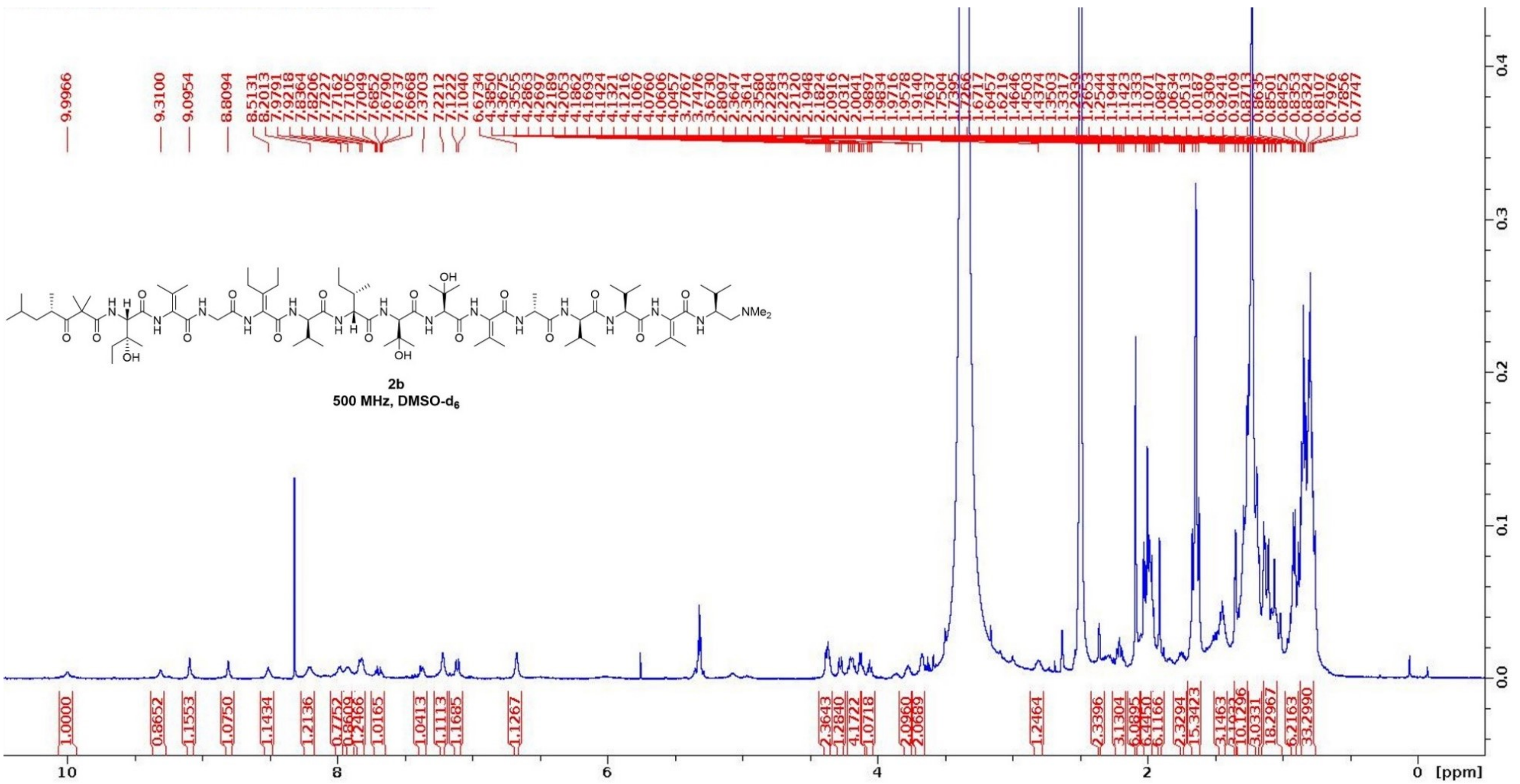
2b
500 MHz, DMSO-d6
[ppm]

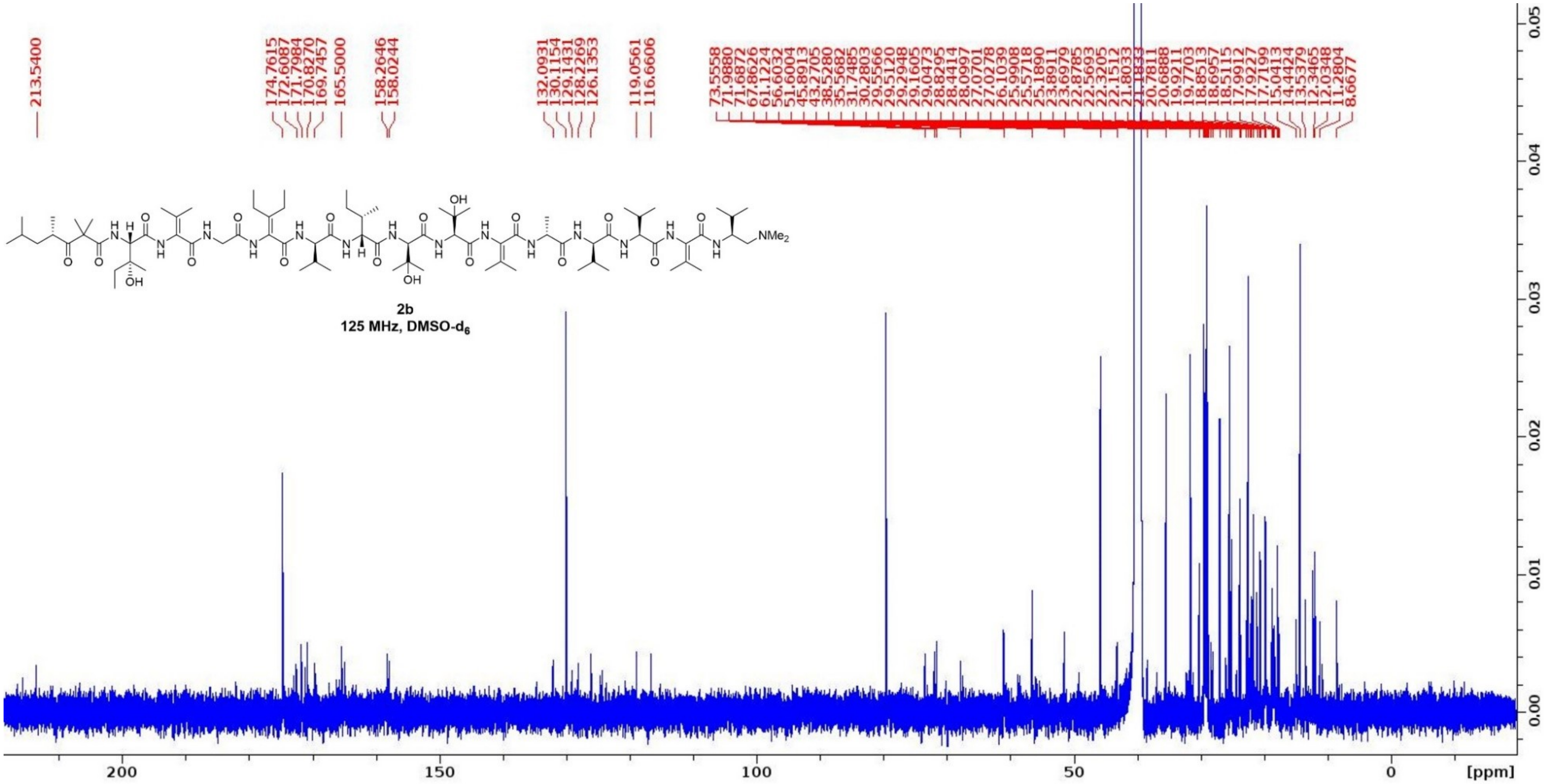
213.5400
174.7615
172.6087
171.7984
170.8270
169.7457
165.5000
158.2646
158.0244
132.0931
130.1154
129.1431
128.2269
126.1353
119.0561
116.6606
73.5558
71.9880
71.6872
67.8626
61.1224
56.6032
51.6004
45.8913
43.2705
38.5280
35.5682
31.7485
30.2803
29.5566
29.5120
29.2948
29.1605
29.0473
28.8295
28.4414
28.0997
27.0701
27.0278
26.1039
25.9908
25.5718
25.1890
23.8911
23.6979
22.8785
22.5693
22.3205
22.1512
21.8033
21.1833
20.7811
20.6888
19.9211
19.7703
18.8513
18.6957
18.5115
17.9912
17.9227
17.7199
15.0413
14.4424
13.5379
12.3465
12.0348
11.2804
8.6677
OH
NMe2
2b
125 MHz, DMSO-d6
200
150
100
50
0
[ppm]
0.00
0.01
0.02
0.03
0.04
0.05

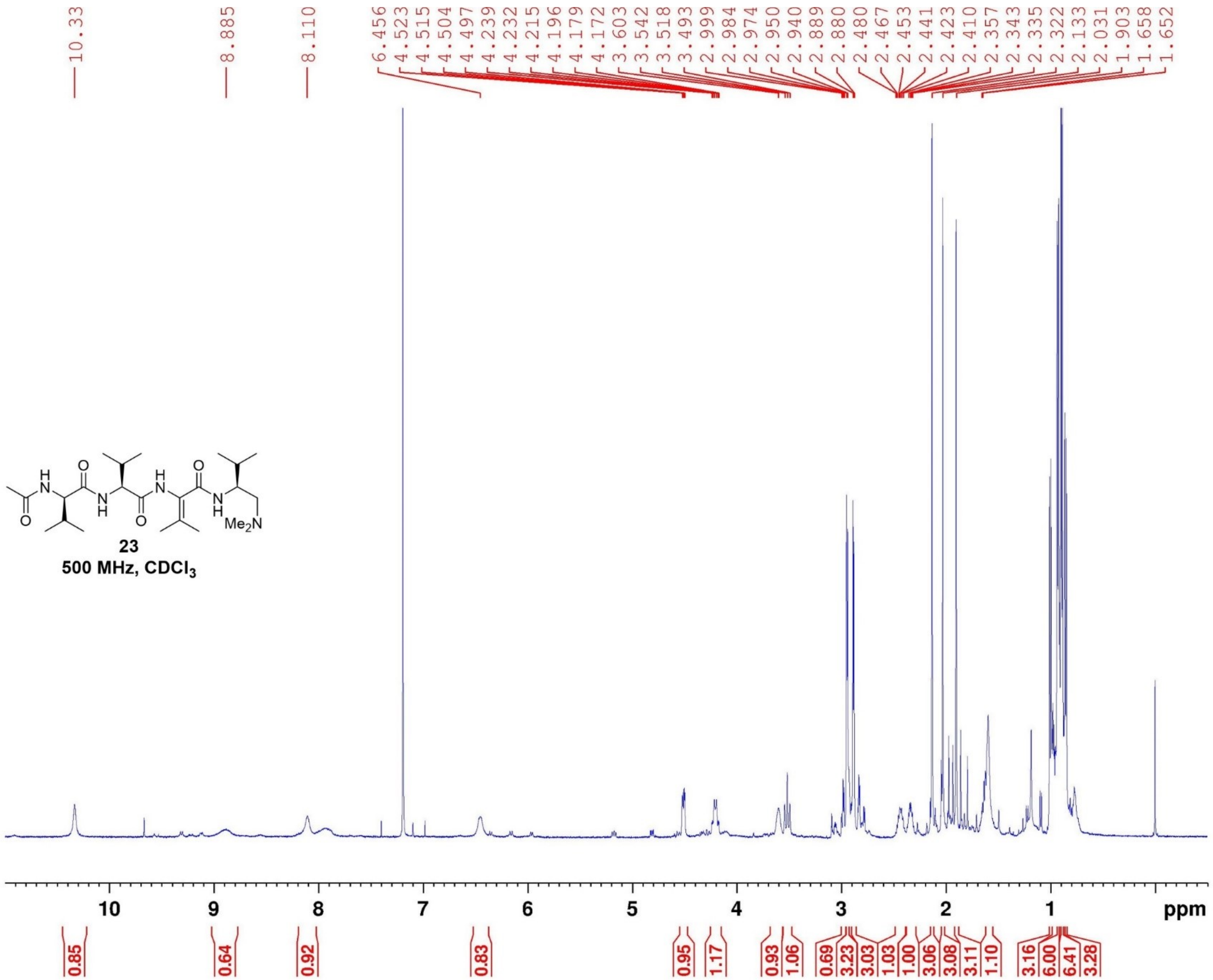

23
500 MHz, $CDCl_3$

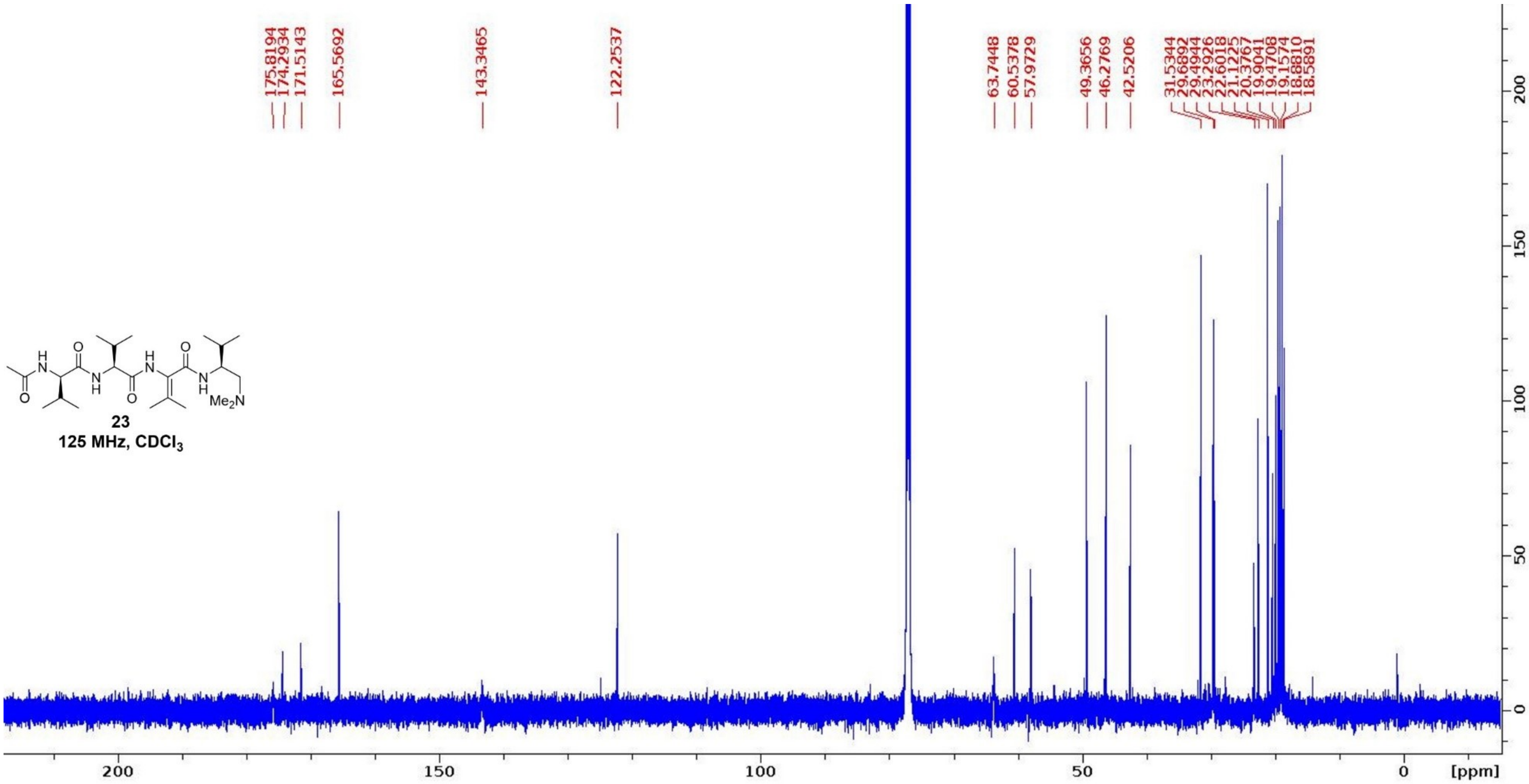
175.8194
174.2934
171.5143
165.5692
143.3465
122.2537
63.7448
60.5378
57.9729
49.3656
46.2769
42.5206
31.5344
29.6892
29.4944
23.2926
22.6018
21.1225
20.3767
19.9041
19.4708
19.1574
18.8810
18.5891
23
125 MHz, CDCl3
[ppm]

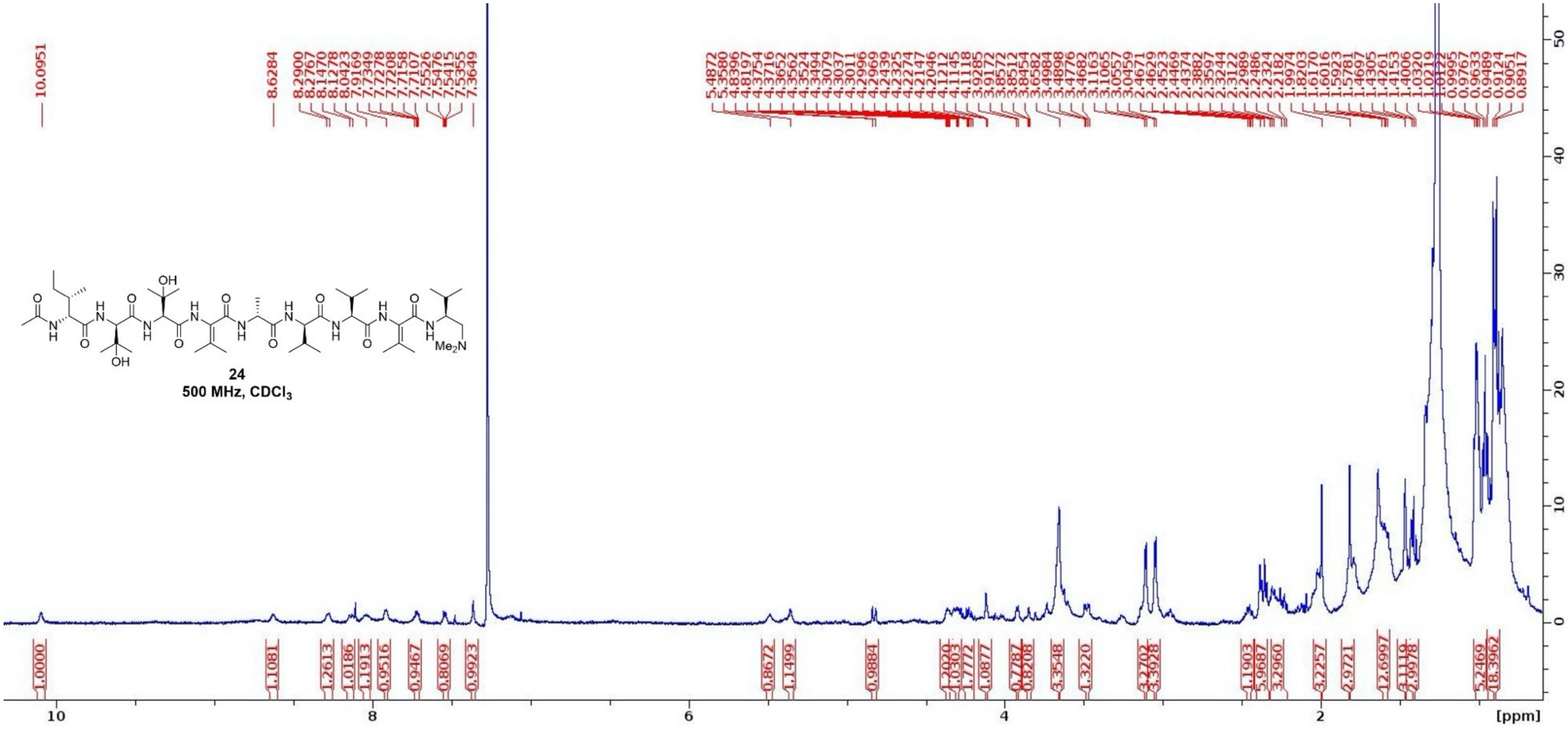

24
500 MHz, $CDCl_3$

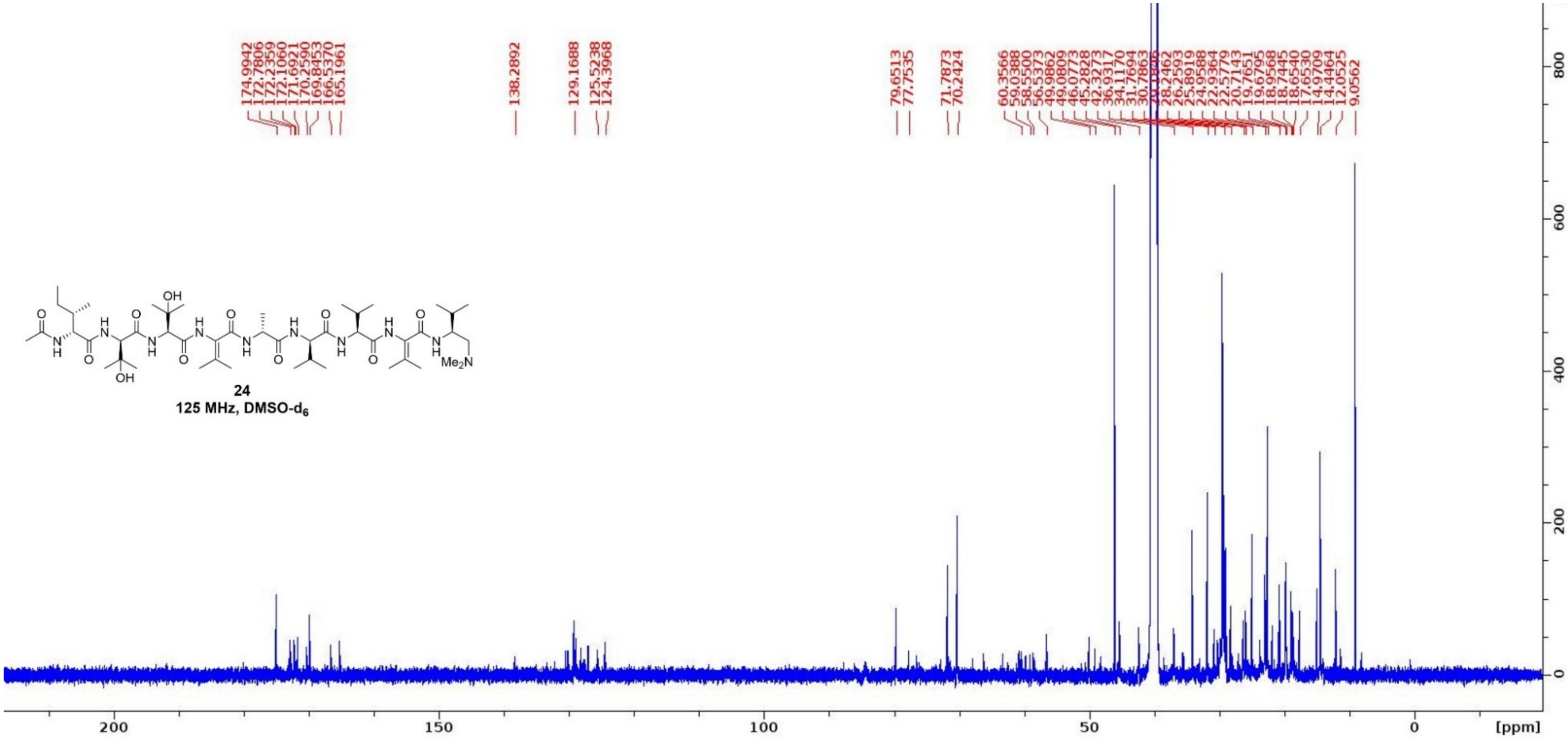

24
125 MHz, DMSO-d6

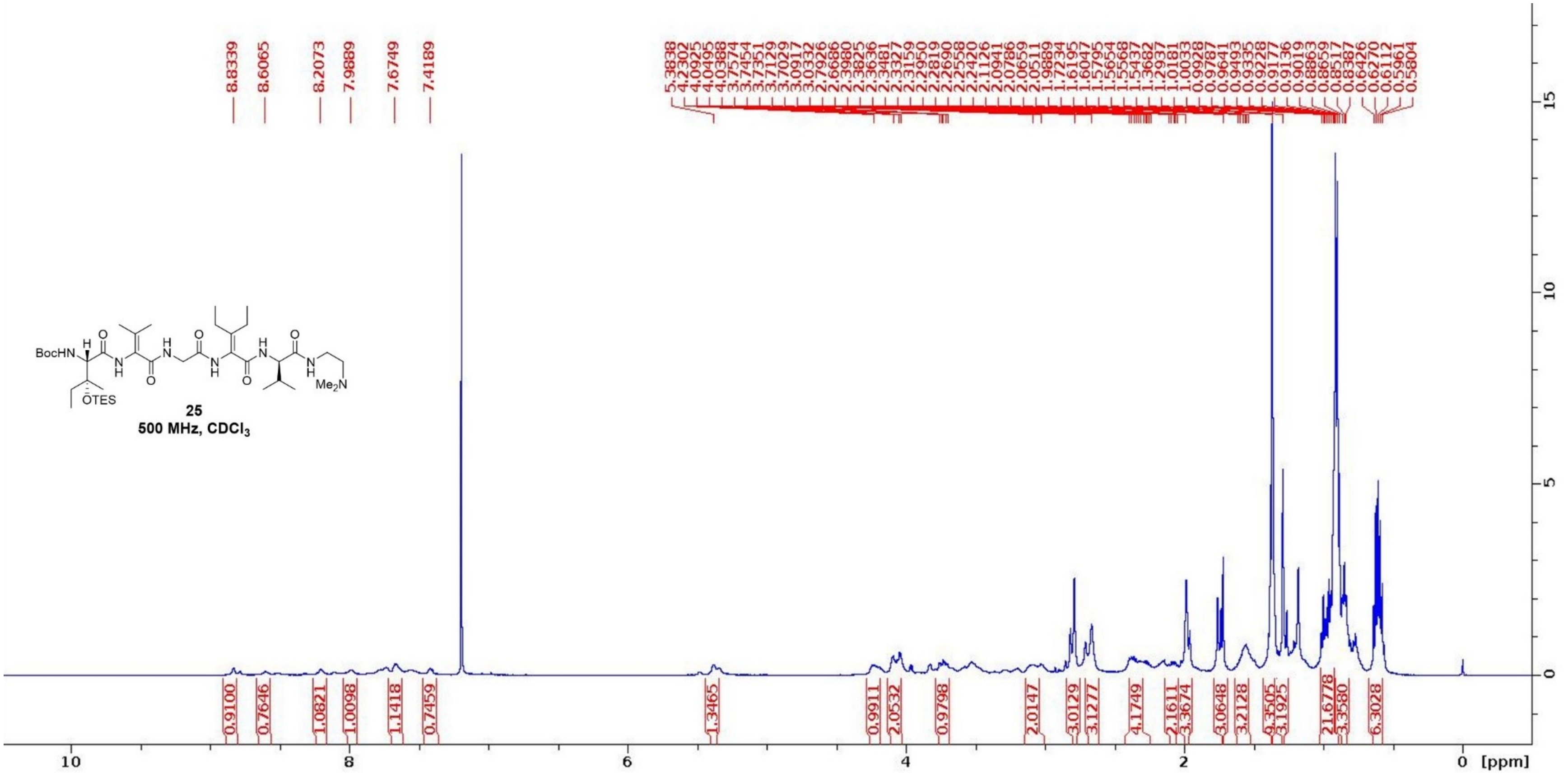
BocHN
OTES
Me2N
25
500 MHz, CDCl3
[ppm]

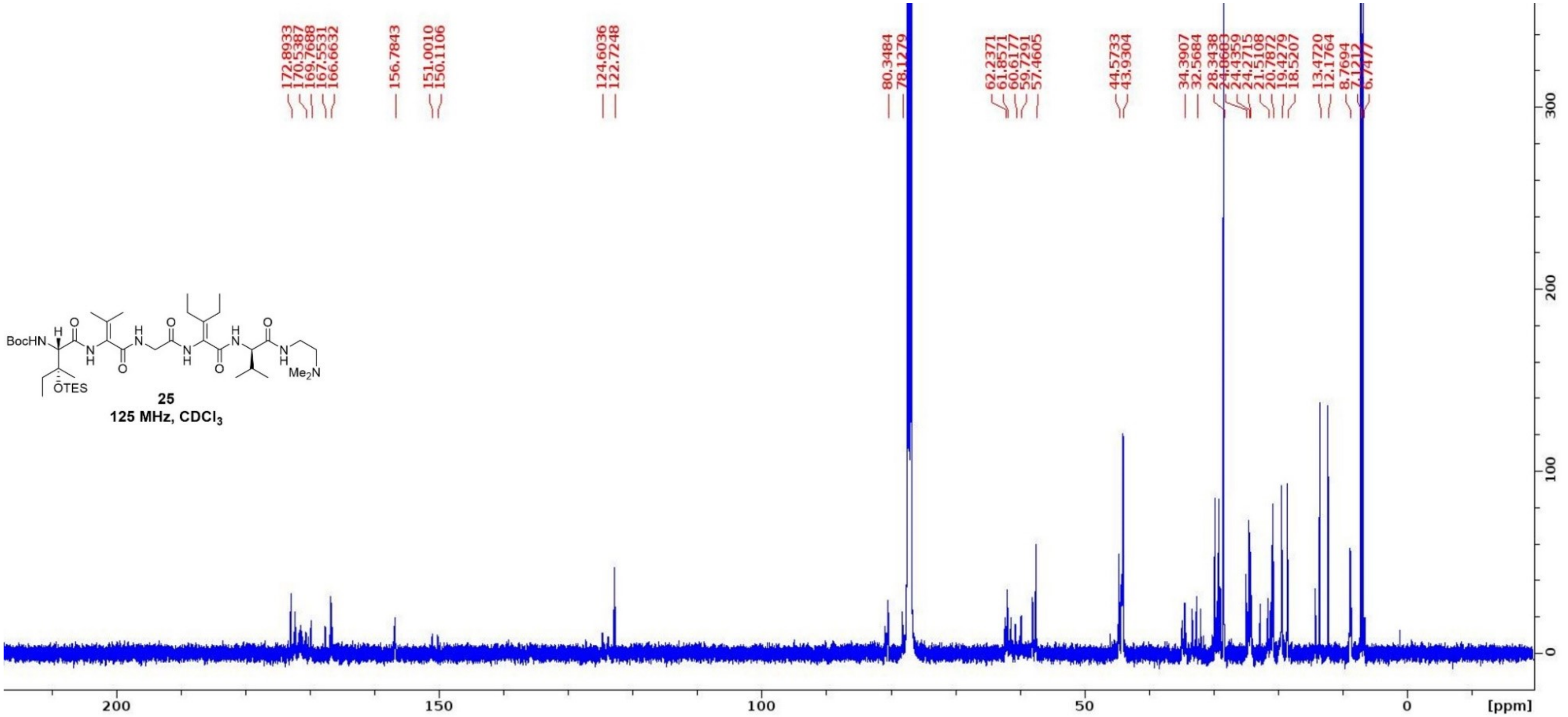
172.8933
170.5387
169.7688
167.5531
166.6632
156.7843
151.0010
150.1106
124.6036
122.7248
80.3484
78.1279
62.2371
61.8571
60.6177
59.7291
57.4605
44.5733
43.9304
34.3907
32.5684
28.3438
24.8683
24.4359
24.2715
21.5108
20.7872
19.4279
18.5207
13.4720
12.1764
8.7694
7.1212
6.7477
BocHN
H
O
N
H
O
N
H
O
N
H
O
N
H
O
N
H
Me2N
OTES
25
125 MHz, CDCl3
300
200
100
0
200
150
100
50
0
[ppm]

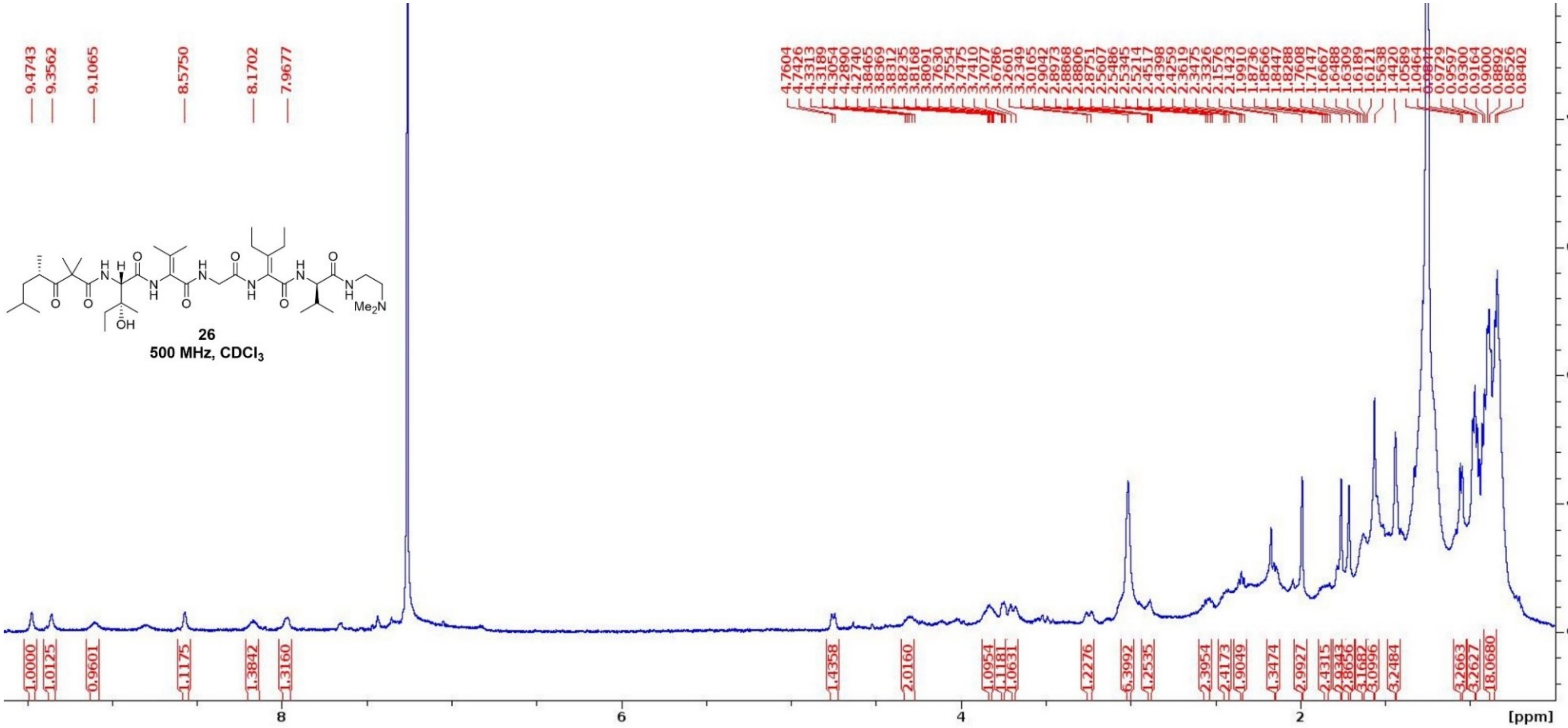

9.4743
9.3562
9.1065
8.5750
8.1702
7.9677
4.7604
4.7426
4.3313
4.3189
4.3054
4.2890
4.2740
3.8465
3.8369
3.8312
3.8235
3.8168
3.8091
3.7630
3.7554
3.7475
3.7410
3.7077
3.6786
3.2601
3.2349
3.0165
2.9042
2.8973
2.8868
2.8806
2.8751
2.5607
2.5486
2.5345
2.5214
2.4517
2.4398
2.4259
2.3619
2.3475
2.3326
2.1576
2.1423
1.9910
1.8736
1.8566
1.8447
1.8288
1.7608
1.7147
1.6667
1.6488
1.6309
1.6189
1.6121
1.5638
1.4420
1.0589
1.0464
0.9729
0.9597
0.9300
0.9164
0.9000
0.8892
0.8526
0.8402
OH
N
H
O
Me2N
26
500 MHz, CDCl3
1.0000
1.0125
0.9601
1.1175
1.3842
1.3160
1.4358
2.0160
1.0954
1.1181
1.0631
1.2276
6.3992
1.2535
2.3954
2.4173
1.9049
1.3474
2.9927
2.4315
3.1682
3.0996
3.2484
3.2663
3.2627
18.0680
8
6
4
2
[ppm]
0
1
2
3
4

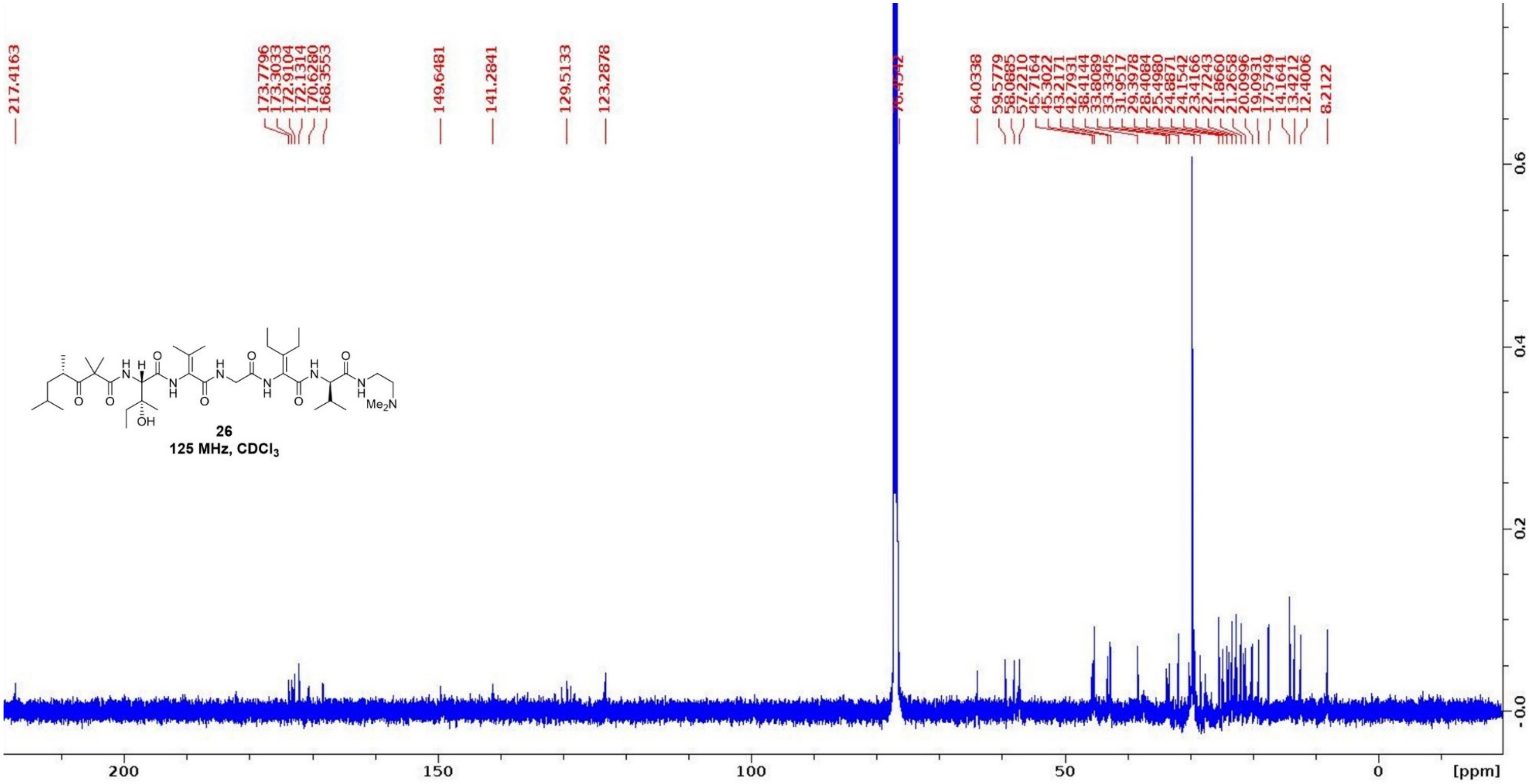
217.4163
173.7796
173.3033
172.9104
172.1314
170.6280
168.3553
149.6481
141.2841
129.5133
123.2878
76.4542
64.0338
59.5779
58.0885
57.2210
45.7164
45.3022
43.2171
42.7931
38.4144
33.8089
33.3345
31.9517
29.3978
28.4084
25.4980
24.8871
24.1542
23.4166
22.7243
21.8660
21.2658
20.0996
19.0931
17.5749
14.1641
13.4212
12.4006
8.2122
26
125 MHz, CDCl3
200
150
100
50
0
[ppm]
0.6
0.4
0.2
-0.0

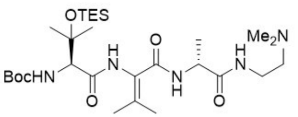

27
500 MHz, CDCl$_3$

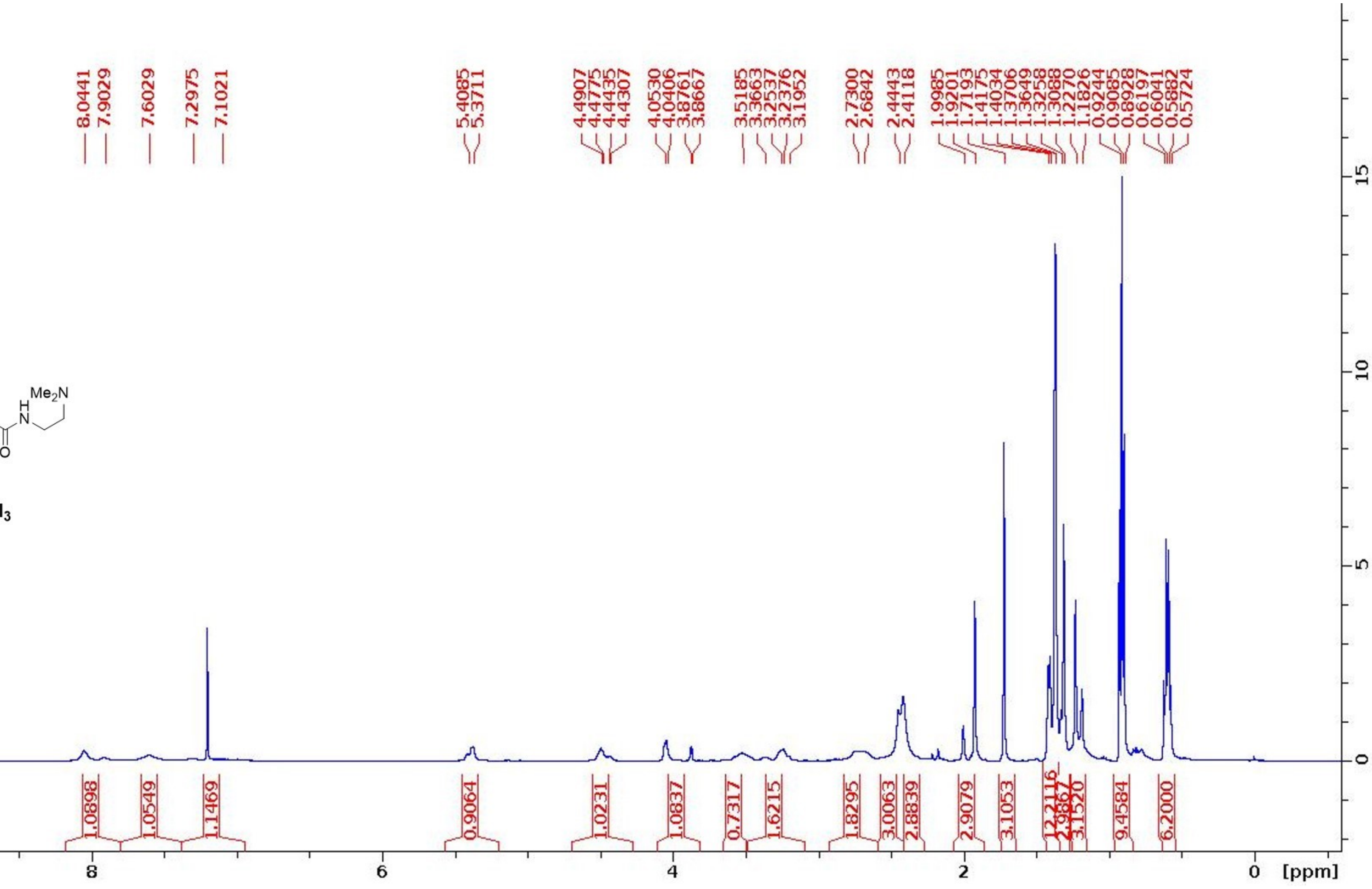

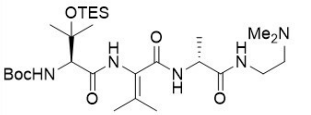

27
125 MHz, $CDCl_3$

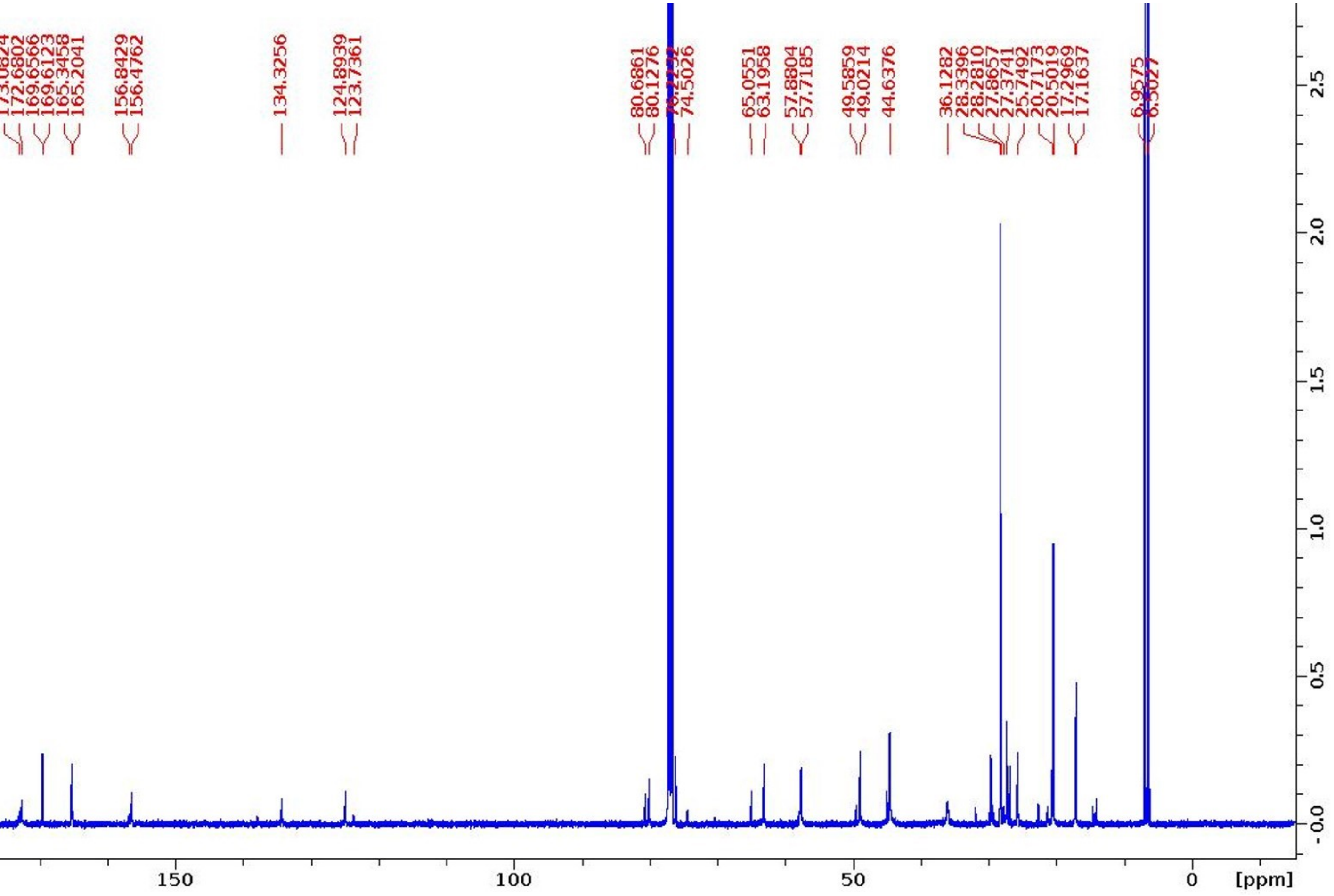

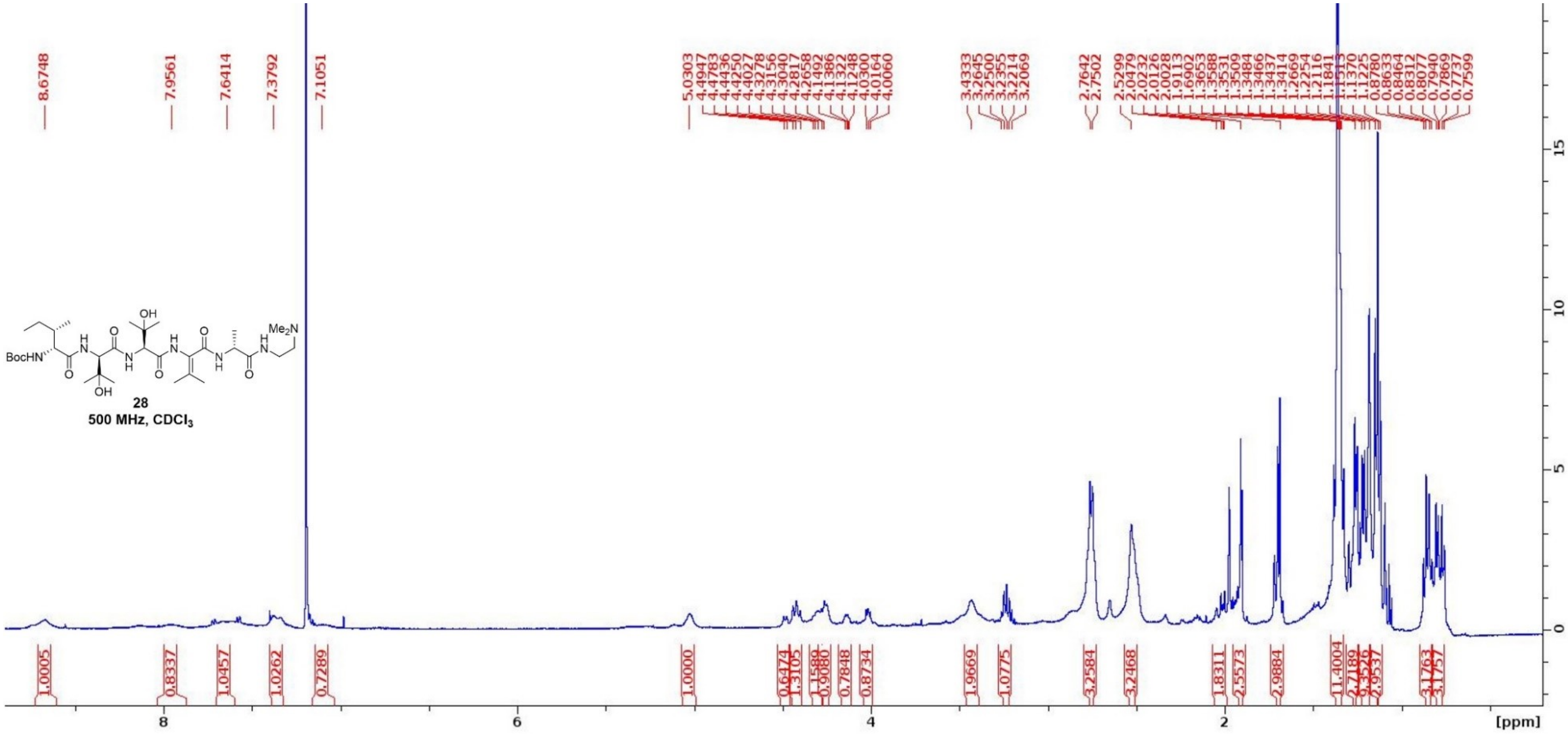

28
500 MHz, $CDCl_3$

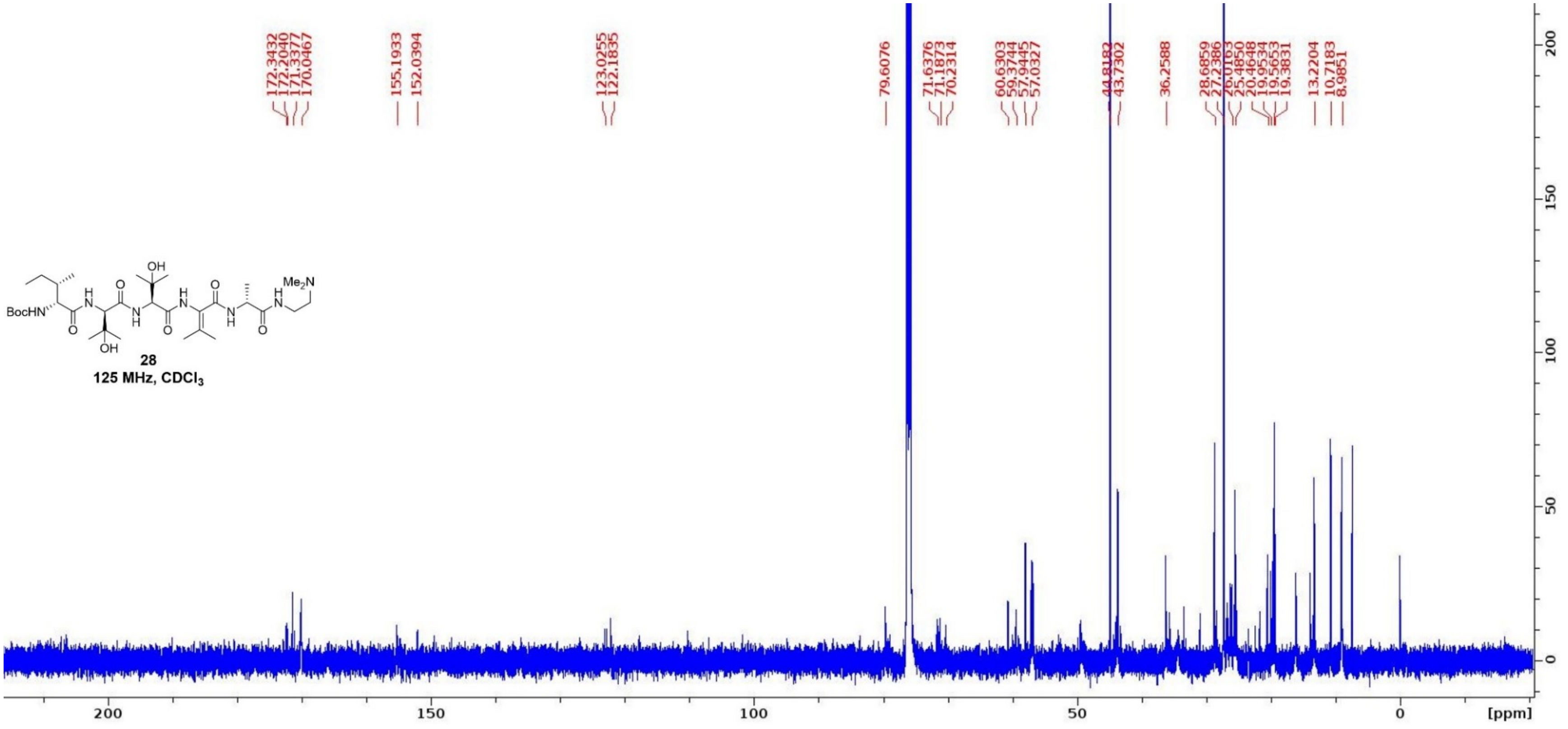
172.3432
172.2040
171.3377
170.0467
155.1933
152.0394
123.0255
122.1835
79.6076
71.6376
71.1873
70.2314
60.6303
59.3744
57.9445
57.0327
44.8182
43.7302
36.2588
28.6859
27.2386
26.0163
25.4850
20.4648
19.9534
19.5653
19.3831
13.2204
10.7183
8.9851
BocHN
OH
Me$_2$N
28
125 MHz, CDCl$_3$
200
150
100
50
0
[ppm]

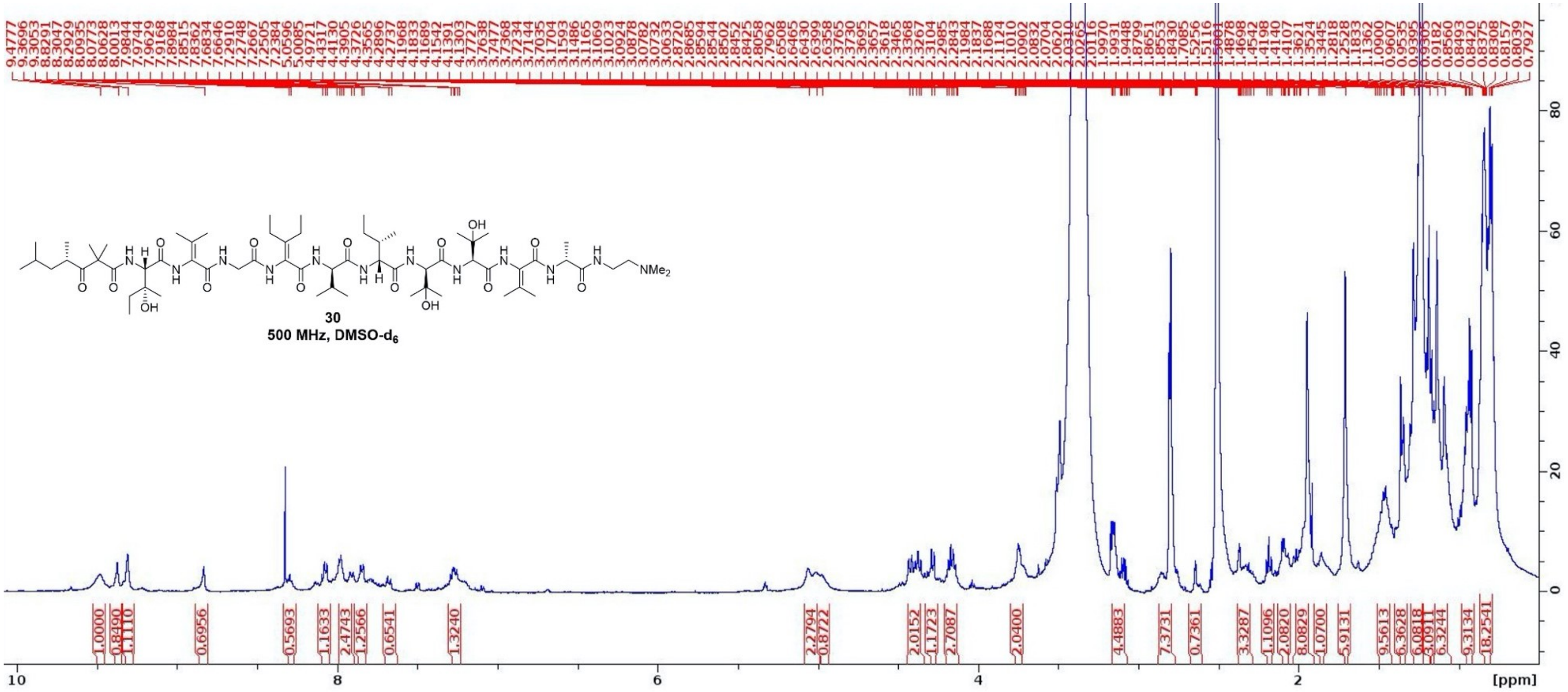

30

500 MHz, DMSO-d_6

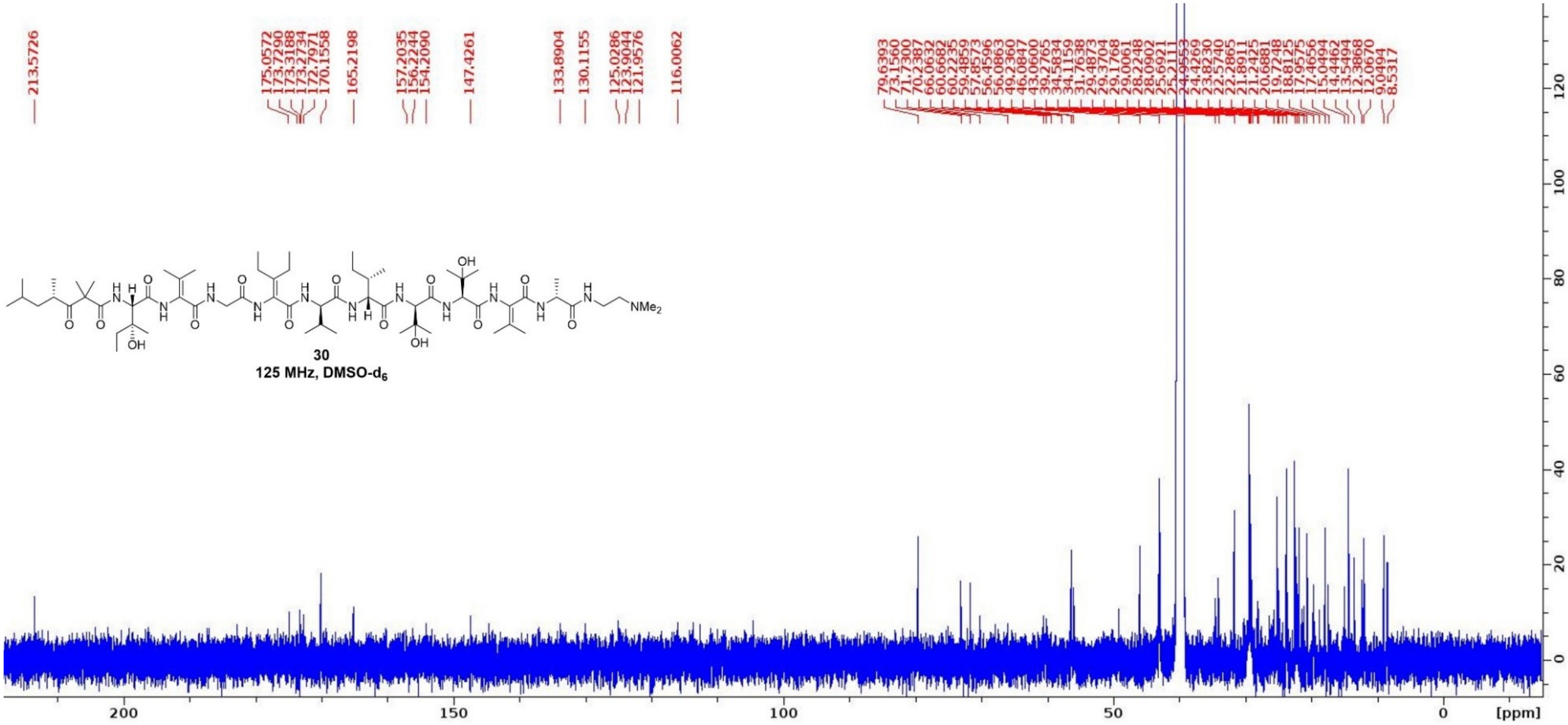
213.5726
175.0572
173.7290
173.3188
173.2734
172.7971
170.1558
165.2198
157.2035
156.2244
154.2090
147.4261
133.8904
130.1155
125.0286
123.9044
121.9576
116.0062
NMe2
30
125 MHz, DMSO-d6
[ppm]
